# Vegetation Dispersion, Interspersion, and Landscape Preference

**DOI:** 10.3389/fpsyg.2022.771543

**Published:** 2022-05-20

**Authors:** Marco Costa

**Affiliations:** Department of Psychology, University of Bologna, Bologna, Italy

**Keywords:** landscape design, vegetation aggregation, plant dispersion, plant interspersion, landscape preference, eye movements

## Abstract

The spatial aggregation/dispersion of the vegetation in a landscape affects landscape texture, with potentially important implications for its perception. The aim of the study was to investigate how plant dispersion and interspersion in small-scale landscapes could affect garden preference. Dispersion referred to the proximity and distance between plants, and interspersion referred to the degree of intermixing between plants of different species. Fifty-six participants evaluated 40 pairs of landscapes that differed in terms of plant dispersion or plant interspersion. Participants were asked to rate their preference for each pair of landscapes. Furthermore, eye movements were recorded during the viewing time, and the number of fixations and fixation time were computed for each landscape image. Overall, plants arranged in a more dispersed and a more interspersed design resulted in a higher landscape preference. Dispersion was more effective than interspersion in affecting landscape preference. The number of fixations and fixation time were higher when viewing landscapes with plants arranged in a high-dispersion and high-interspersion layout.

## Introduction

The law of proximity and the law of similarity are among the main principles of perceptual organization in the visual domain (Wertheimer, 1938). They state that visual elements that are in close proximity and visual elements that are similar tend to be grouped into unitary and distinct patches that are segregated from the background. Considering environmental perception and landscapes, when visual elements are in close proximity, they tend to form dense and homogenous patches, whereas when their distance and variety are increased, they tend to form spaced and heterogeneous patches.

Aggregation refers to the spatial clustering of the patches in a landscape (He et al., 2000). The patches are areas of perceptual uniformity within a landscape. Aggregation strongly affects landscape texture and is mainly operationalized in terms of dispersion and interspersion. Dispersion refers to the spatial distribution of patches belonging to the same category (i.e., how spread out and distant they are), while interspersion refers to the spatial intermixing of patches belonging to different categories. In the context of this study, dispersion refers to the distance between plants of the same species, while interspersion refers to the degree of intermixing between plants of two distinct species. Therefore, a low-aggregation landscape results either from plants displaced farther apart or from plants of different species being intermixed, while a high-aggregation landscape results from a layout in which plants are placed in close proximity and/or segregated by species in distinct homogeneous patches. Previous studies have investigated the effect of vegetation density on landscape preference, considering mainly high-scale landscapes. In these studies, landscapes were segmented into homogeneous patches according to some high-order criterion (e.g., land use), and the patch mosaic was analyzed with specific indexes as patch size, patch size coefficient of variation, total edge, landscape complexity, mean patch fractal dimension, class density, and Shannon's diversity index (Sawalha and Sayed, 2001; Sertel et al., 2018; Di Cristofaro et al., 2020). The interaction of people with green spaces, however, is usually on a much lower scale than gardens and sections of parks, and is therefore important to assess the factors that contribute to landscape preference on this small scale.

Landscapes rich in greenery, when compared to urban landscapes, tend to have smaller patch sizes, higher variation in patch sizes, an increased number of edges, a higher fractal dimension, and higher diversity metrics (class density and Shannon's diversity index) (Di Cristofaro et al., 2020).

Vegetation aggregation can affect specific landscape metrics strongly related to human landscape preference. For example, when plants are aggregated by distance or variety, the landscapes tend to be more compact, with greater patches, lower spatial frequencies, and a reduction of edges and edges density. Fragmentation of patches is therefore decreased, also affecting landscape complexity (Sang et al., 2008).

The main models aimed to explain landscape preference are framed in the evolutionary theory, suggesting a universal human preference toward those environments that maximize survival, protection, and resources. For example, the Preference Matrix by Kaplan and Kaplan (1978, 1982, 1989) is an evolutionary theory that links landscape aesthetics to the appraisal of habitats that could offer an advantage in evolutionary terms. The four predictors of the preference matrix are coherence, complexity, legibility, and mystery. Coherence and legibility contribute to landscape understanding and the ability to abstract a cognitive map. Complexity and mystery assess the level of richness and diversity of a landscape. When vegetation aggregation is decreased (i.e., plants are more spaced and intermixed for variety), the landscape tends to increase in complexity and mystery because the unifying effect of proximity between plants is weakened. On the contrary, a decrease in vegetation dispersion and/or interspersion tends to result in an increase in coherence and legibility because plants tend to be aggregated in larger patches.

Another evolutionary theory related to landscape preference is the savanna hypothesis, which suggests a preference for those environments that have ensured the greatest survival rate during evolution (Orians and Heerwagen, 1992; Falk and Balling, 2010; Townsend and Barton, 2018). Savanna environments are characterized by open spaces, with shrubs and sparse trees, offering both perspective and refuge, in contrast to the denser environment such as forests, rainforests, and habitats of other primates (Orians and Heerwagen, 1992; Appleton, 1996; Summit and Sommer, 1999; Falk and Balling, 2010). This theory, however, has been challenged by some studies that found a preference for landscape images similar to the environmental context in which participants lived (Balling and Falk, 1982; Lyons, 1983; van den Berg et al., 1998; Hartmann and Apaolaza-Ibáñez, 2010; Moura et al., 2018). Balling and Falk (1982) showed that the preference for a specific biome tends to change with age. They found an enhanced preference for the savanna biome in children and a broader preference for savanna, deciduous, and coniferous biomes compared to rainforests and desert biomes in adolescents and adults. Overall, savanna and open forest landscapes, with a low level of vegetation aggregation, tended to be highly preferred, while thick forest, jungle, and desert scenes, with a high level of vegetation aggregation, were clearly disliked.

In two studies, the degree of expertise was demonstrated to be a critical factor in the importance of visual openness for landscape preference. Tveit (2009) found that the degree of open land in a landscape was a predictor of preference for students in landscape professions but not for the general public. Similarly, Hägerhäll et al. (2018) found that the preference for half-open landscapes was valid only for students in landscape architecture.

Regarding the dichotomy between natural vs. built environments, the research has consistently found natural environments to be preferable over man-made environments (Kaplan and Kaplan, 1989). However, the dichotomy is mitigated by the fact that, for most people, the category of natural environments includes not only wilderness but also agricultural landscapes, gardens, and sports facilities that are strongly shaped by humans (Ulrich, 1993). Environments with greater tree cover are preferred over settings with less tree cover (Sommer and Summit, 1995). Jiang et al. (2015) assessed landscape preference as a function of tree canopy density and found that the relation was best fitted by a power function. Planting trees in relatively treeless residential areas offer much greater impact than the same trees planted in an already green area. They found that, to ensure a moderate preference value, tree cover density should not be less than 41%, considering an eye-level panoramic measure, and 20%, considering a top-down aerial measure. A similar result was reached by Suppakittpaisarn et al. (2020), who investigated the role of vegetation density on green stormwater infrastructure preference, and by Yang et al. (2009), assessing the visibility of urban forests in cities. These studies manipulated density as the number of trees, but in this study, the density was kept constant while the spatial arrangement of plants varied.

Previous studies on human perception have shown that the level of stimuli aggregation is a critical factor in determining the preference for visual stimuli. For example, Maisel and Karmel (1978) have studied visual preferences in newborns for contour patterns that varied for contour density. The average duration of fixation was higher in patterns with low contour density than in stimuli with high contour stimuli. This result was valid for all the patterns that were investigated: checkerboard, concentric squares, radial lines, and concentric circles.

This study aimed to explore a specific aspect of landscape complexity: the level of plant aggregation. Aggregation was declined in two ways: dispersion and interspersion. Dispersion was defined as the spacing in which plants are arranged. When spacing is low, plants are arranged to form a compact design. According to the law of proximity, in this case, plants tend to be perceived in highly segregated patches. In the case of high dispersion, plants are arranged more sparsely, with a reduction of their perceptual segregation. Interspersion was defined as the segregation/intermixing of plants of different species within a landscape. In the high-aggregation condition, each species is grouped together and segregated from the others, whereas in the low-aggregation condition, plants of different species are intermixed.

Landscape preference was assessed using two methods: self-report ratings and eye movement recording. For eye movements, two indexes were analyzed: the number of fixations and the total time of fixations to each stimulus. Eye movements were included in the methodology as a more fine-grained and spontaneous measure of visual preference and visual interest that would complement self-report ratings. Previous research has shown that people focus more visual attention on stimuli that are perceived as attractive (e.g., Garza et al., 2016; Jankowski et al., 2020). To allow a more controlled and accurate comparison of stimuli between the different conditions, landscapes were presented in pairs. Within each pair, the landscapes were identical with the exception of the manipulation of vegetation dispersion or interspersion.

The participants in the study were university students. A sample of experts in landscape design or gardening was not selected to increase the generalizability of the results to the general population.

## Method

### Participants

Fifty-six university students (25 men and 31 women) participated voluntarily in the study. The mean age was 23.33 years (*SD* = 4.40). Students wearing glasses were excluded because glasses were incompatible with the eye-movement recording equipment. The number of participants was selected considering *a priori* power analysis using G^*^Power (Faul et al., 2007), setting an effect size of 0.15 and a power level of 0.95.

The experimental protocol was approved by the Ethics Committee of the University of Bologna. All participants provided written informed consent. They were free to abandon or withdraw from the study at any time. To avoid influencing the participants, the study was presented with the general aim to assess landscape preferences. The specific aim of the role of vegetation dispersion and interspersion on landscape preference was not revealed until the end of the study.

### Materials

**Figure 1:**
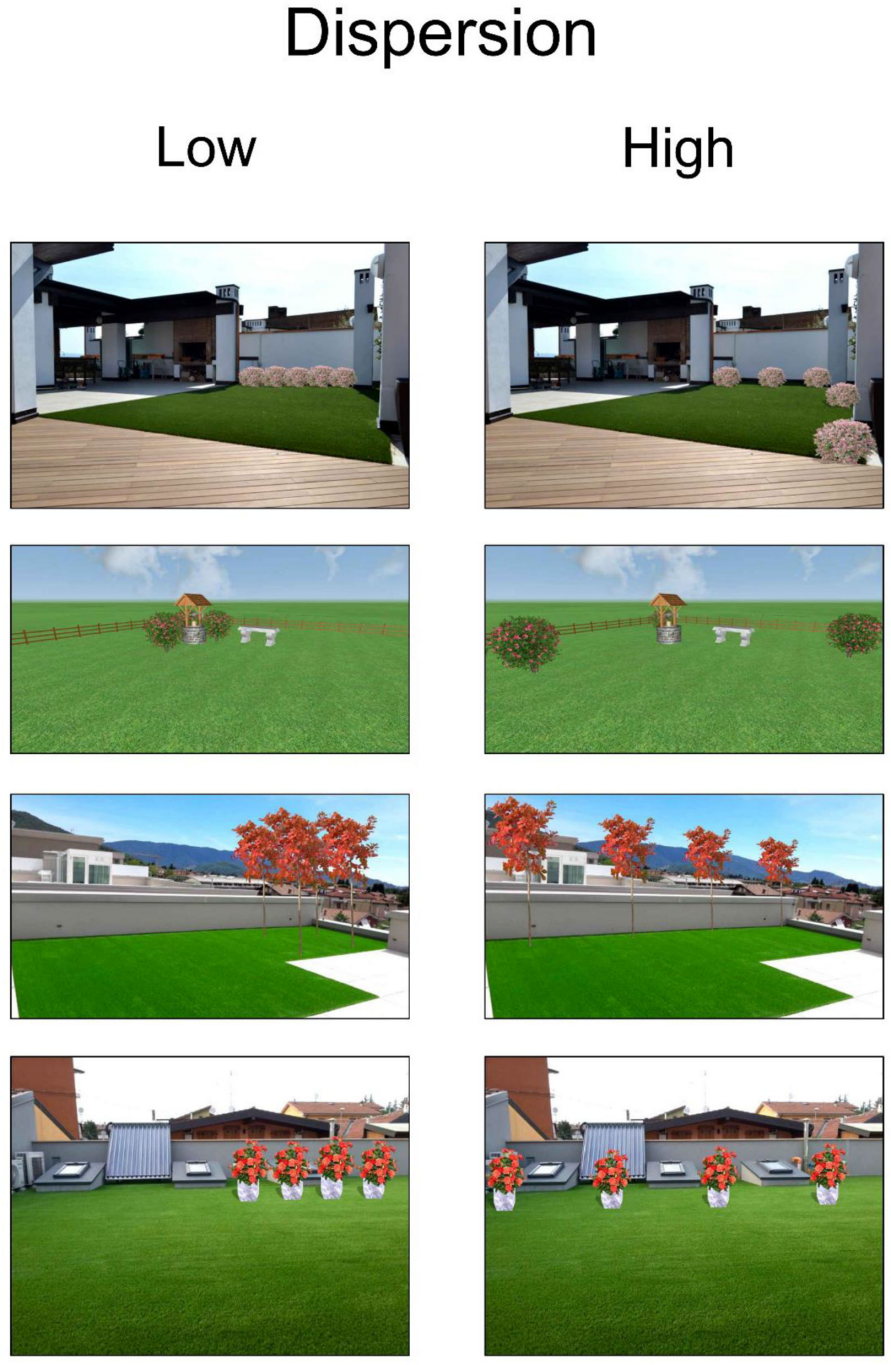
Examples of landscape pairs varying for plant dispersion. Images created with Realtime Landscaping Architect. Reproduced with permission.

**Figure 2:**
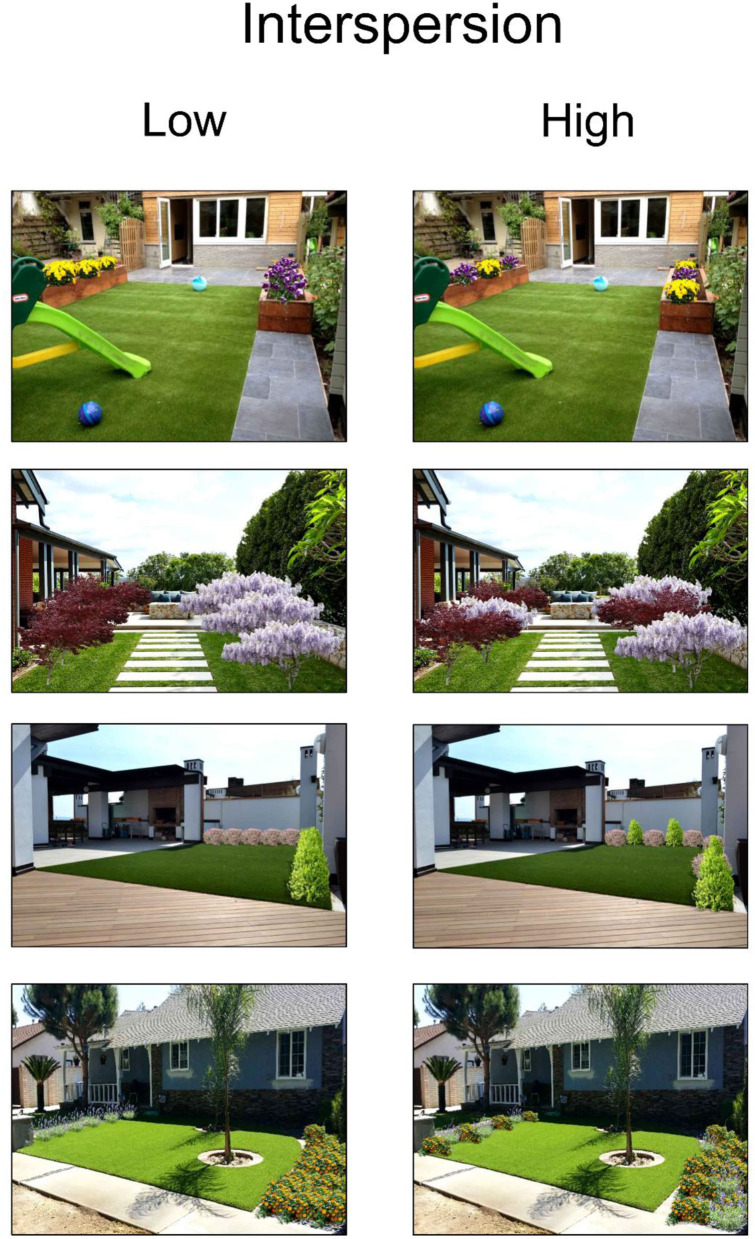
Examples of landscape pairs with low and high varietal interspersion. Images created with Realtime Landscaping Architect. Reproduced with permission.

Forty pairs of landscape renderings, in which vegetation aggregation was selectively manipulated, were created using Realtime Landscaping Architect, a software for 3D landscaping design. In 23 pairs, the spacing between plants was varied while maintaining all other landscape features constant (Figure 1) (“Dispersion” condition), whereas plant interspersion was varied in 17 pairs (“Interspersion” condition). Plants belonging to two distinct species were divided in the low-interspersion condition, whereas in the high-interspersion condition, the plants were intermixed (Figure 2). The 40 pairs of landscapes are available in the Supplementary Material. The experimental sequence was programmed in E-Prime 2.0 Professional on a desktop PC. The video output was connected to a beam projector, and the stimuli were presented in a screen projector 1.25 m x 1.25 m to increase their immersiveness, the subtended angle of presentation (41.11° × 41.11°), and therefore, the precision of eye-movement localization. The participant was seated at a 2 m distance from the screen, with the head positioned on a chin rest and a headrest to avoid large head movements that would have adversely affected the accuracy of the eye-movement recording.

Eye movements were recorded with a Pupil Labs mobile eye-tracker with a sample rate of 30 Hz and a resolution of 1,920 × 1,080 pixels.

### Procedure

After a presentation of the study and the acquisition of the informed consent, the mobile eye-tracker device was applied, tested, and calibrated. In the calibration phase, a 3 × 3 dot matrix was presented on the projection screen, and the participant was instructed to fixate each dot, therefore recording the foveal position on the visual scene.

The study included two testing trials to familiarize the participant with the procedure and 40 experimental trials. The presentation order of the 40 experimental trials was randomized between participants. Between trials, the left-right presentation of the high-aggregation and low-aggregation stimuli was counterbalanced. Each trial consisted of a 10-s presentation of the pair of stimuli. The two stimuli of each pair were aligned horizontally and were of exactly of the same size (subtended angle: width 20.56°, height 15.81°). This phase was aimed to record the participant's eye movement while they scanned the two stimuli; no rating was requested.

After 10 s, the stimuli were reduced by 45% in size and were placed in the upper-middle part of the projection screen. In the lower part of the projection screen, a visual Likert scale was presented with seven options. The scale asked the participants to indicate which of the two landscape images within the pair was preferred. Three options were positioned under the left image, three options under the right image, and one option was centered horizontally between the two images. The participant was instructed that the center option expressed the equivalence of preference between the two landscapes and that the options progressively to the left or to the right were for expressing a progressive preference for the left or right landscape. The rating was not time limited. After the rating was complete, a fixation point at the center of the projection screen was presented for 3 s, followed by the beginning of a new trial. All the ratings were automatically recorded by E-Prime software.

### Data Analysis

#### Fixations

Two regions of interest (ROI) were defined, the areas of the left stimulus and that of the right stimulus. The number of fixations and cumulative duration of all fixations to each ROI were computed with the Pupil Player software. A fixation was defined as the permanence of the pupil center in an area of 3° for at least 200 ms (van Gompel et al., 2007).

Frequency of fixations and cumulative fixation durations were analyzed using the linear mixed-effects model (Laird and Ware, 1982; Pinheiro and Bates, 2000). The aggregation level (high/low) and aggregation type (dispersion/interspersion) were included as fixed-effect predictor, and the participant was included as a random factor. Each fixed effect was inserted sequentially in the model to test if it contributed significantly in increasing the model's validity. Each model was fit by maximizing the log-likelihood and assessed using the Akaike information criterion (AIC). All statistical computations were performed using R (version 4.0.3).

#### Ratings

**Figure 3:**
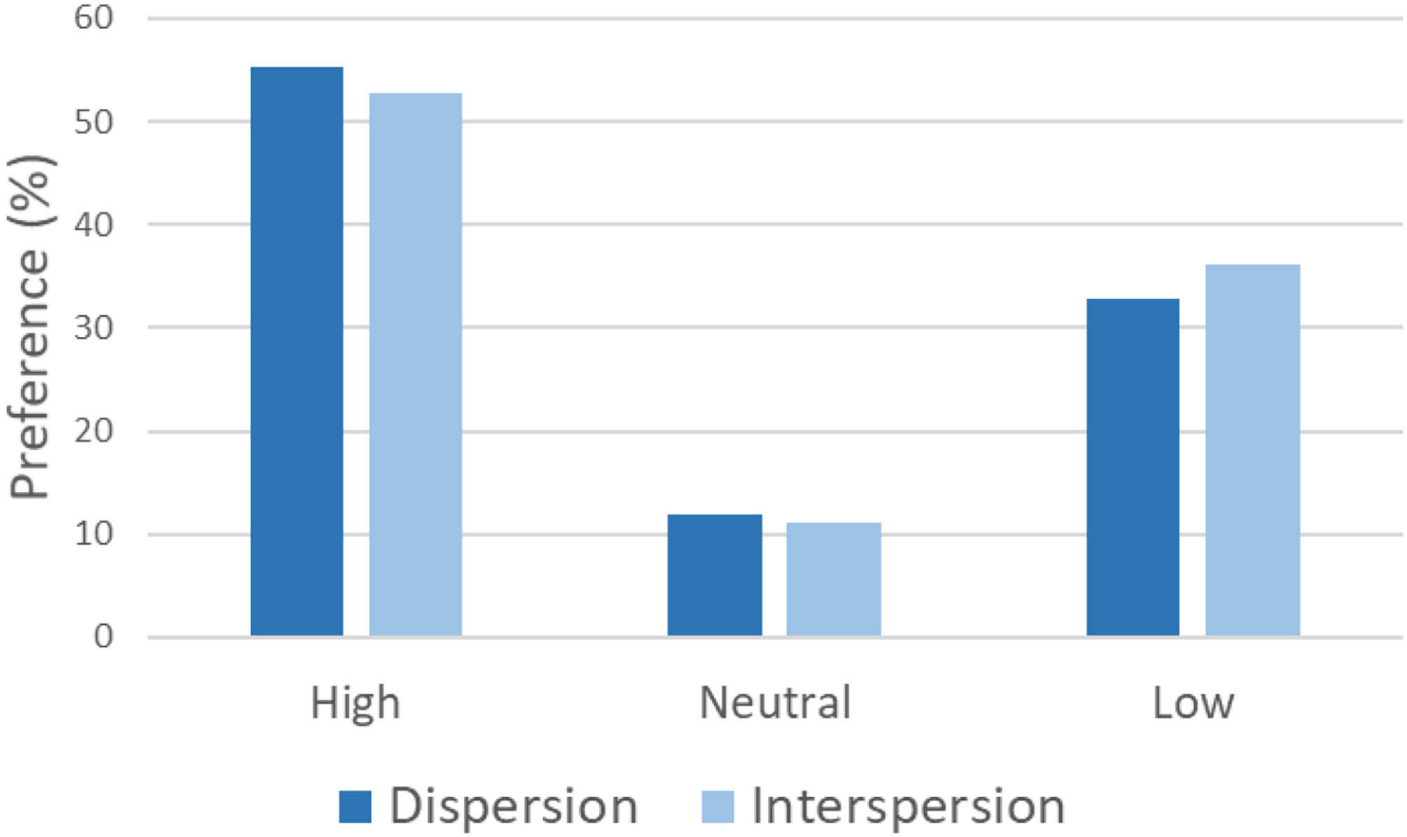
Distribution of preferences for landscapes differing in dispersion and interspersion levels. Neutral showed the cases in which participants expressed an intermediate preference between the “high” and “low” specimens.

The ratings for preference were coded from −3 to 3, where negative values indicate a preference for the stimulus presented to the left and positive values indicate a preference for the stimulus presented to the right. A zero value was assigned when the participant expressed neutrality between the two stimuli (Neutral condition in Figure 3).

The analyses for ratings were performed in two steps. First, we converted the Likert values to a categorical variable with three levels: preference for the low-aggregation landscape, preference for the high-aggregation landscape, and neutral (equal preference for both landscapes). The frequencies were then compared statistically with the Chi-square test. The frequency analysis resulted in a global preference index as a function of the vegetation aggregation level. In the second analysis, the numerical values from the seven-degree Likert scale were compared with a one-sample *t*-test to the control value of 0 (a null hypothesis that low-aggregation and high-aggregation landscapes did not differ in preference). This second analysis allowed a precise quantification of landscape preference as a function of the vegetation aggregation level. In the third analysis, the numerical ratings were analyzed by applying a linear mixed-effect model, inserting aggregation type (dispersion, interspersion), and aggregation level (high, low) as fixed-effect predictors and participant as a random factor. This last analysis assessed using a unique linear model the role of individual differences (random factor), aggregation type (predictor), and aggregation level (predictor) on landscape preference ratings.

Since the side of the low-aggregation stimulus was counterbalanced between trials, the side associated with negative values in the Likert scale was standardized during data analysis by reversing all the cases in which the low-aggregation stimulus was presented to the right. As a result of this standardization, negative values indicated a preference for the low-aggregation stimulus, and positive values indicated a preference for the high-aggregation stimulus.

## Results

### Categorical Data—Dispersion

The distribution of choices was 55.38% for the high-dispersion landscape (*N* = 704), 32.80% for the low-dispersion landscape (*N* = 417), and 11.80% for no preference (Figure 3). The Chi-square test was significant when comparing the low- and high-dispersion preferences: χ^2^ = 130.53, *p* < 0.001 (−0.26, −0.18), ϕ = 0.34.

### Categorical Data—Interspersion

The distribution of choices was 52.71% for the high-interspersion landscape (*N* = 495), 36.20% for the low-interspersion landscape (*N* = 340), and 11.07% for no preference (Figure 3). The Chi-square test was significant when comparing the low- and high-interspersion preferences: χ^2^ = 51.14, *p* < 0.001 (0.11, 0.21), ϕ = 0.25.

### Ratings

A one-sample *t*-test compared the preference ratings with a control value of 0 (neutrality in preference). For dispersion, the *t-*test was significant: *t*_(1, 268)_ = 9.07, *p* < 0.001, 95% CI (0.36, 0.57), Cohen's d = 0.20. The mean rating was 0.46 (*SD* = 1.83). A positive value indicated a preference for the high-dispersion garden design. For interspersion, the *t-*test was also significant: *t*_(938)_ = 4.48, *p* < 0.001, 95% CI (0.15, 0.40), Cohen's d = 0.15. The mean rating was 0.28 (*SD* = 1.88).

A linear mixed model examined the effect of the participant as a random factor and the effect of the type of aggregation (dispersion and interspersion) as a fixed factor. Table 1 shows that aggregation type significantly affected garden preference. The parameter estimates of the linear mixed model are reported in Table 2.

**Table 1:** Linear mixed model results for landscape preference as a function of aggregation type (dispersion vs. interspersion).

**Model**	**df**	**AIC**	**χ^2^**	***P***
1. Intercept	3	8,928.86		
2.1 + Aggregation type	4	8,924.75	6.10	0.01
3.2—Random effect (Participants)	3	9,000.51	77.75	<0.001

*The participant was included as a random factor*.

**Table 2:** Parameter estimates in the linear mixed model for landscape preference rating as a function of aggregation type.

**Predictor**	***B***	***SE***	***T***	***P***
(Intercept)	0.46	0.08	5.71	0.001
Aggregation type	−0.19	0.07	−2.47	0.01

**Figure 4:**
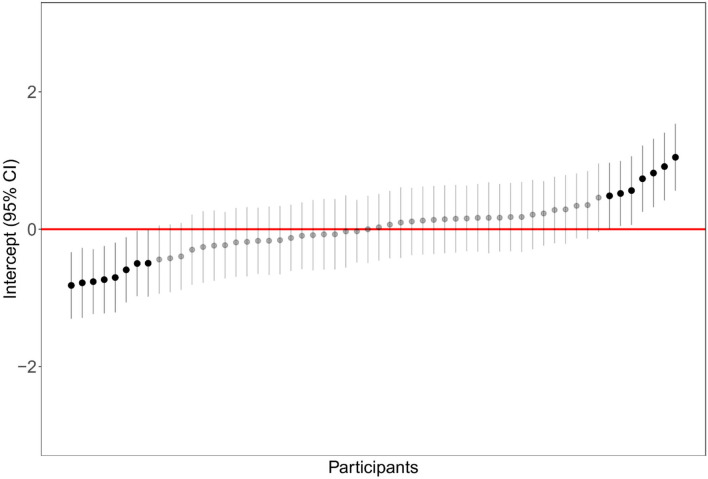
Distribution of participants' intercept (random effect) in the linear mixed model examining landscape preference as a function of aggregation type.

The preference for low-aggregation landscapes was higher when considering dispersion [*M* = 0.46, *SE* = 0.08, 95% CI (0.30, 0.63)] than when considering interspersion [*M* = 0.27, *SE* = 0.08, 95% CI (0.10, 0.45)]. The random effect referred to participants was highly significant, and the distribution of intercepts is shown in Figure 4.

### Fixation Frequency

**Figure 5:**
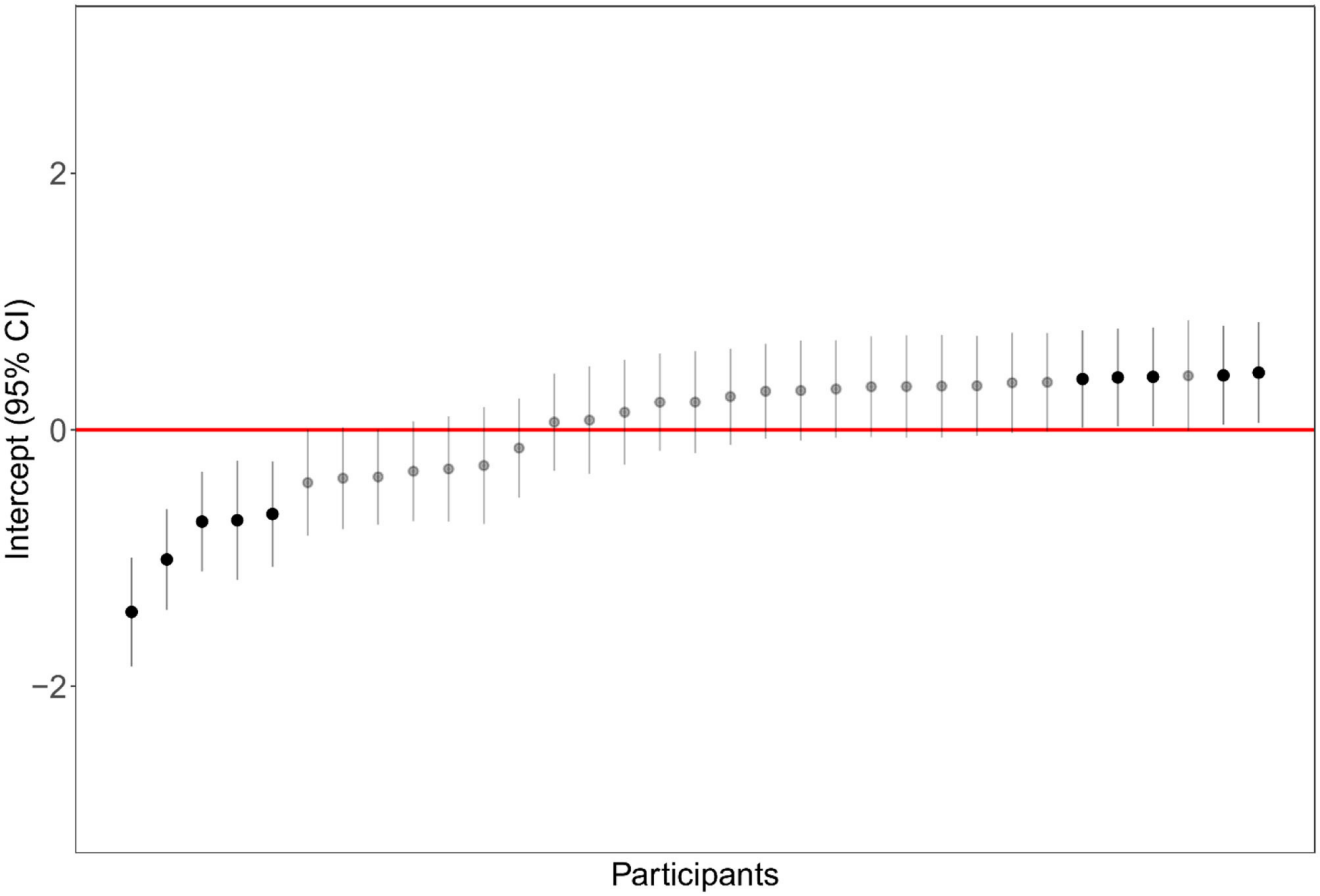
Distribution of participants' intercept (random effect) in the linear mixed model testing number of fixations as a function of aggregation level and aggregation type.

A linear mixed model evaluated the effect of dispersion and interspersion on the number of fixations. The participant was chosen as a random factor. The results of the model are reported in Table 3, and the parameter estimates of the linear model are shown in Table 4. The fixation number was significantly influenced by the level of aggregation (high, low) and by the random factor participants. The aggregation type (dispersion vs. interspersion) was not significant. The mean number of fixations was 4.74 (*SE* = 0.01), 95% CI (4.53, 4.95) in the low-aggregation conditions and 4.15 (*SE* = 0.10), 95% CI (3.94, 4.35) in the high-aggregation condition. The distribution of intercepts for the random effect participants is shown in Figure 5.

**Table 3:** Linear mixed model results for fixation number as a function of the level of aggregation (low-high) and aggregation type (dispersion-interspersion).

**Model**	**df**	**AIC**	**χ^2^**	***p***
1. Intercept	3	10,027.69		
2.1 + Aggregation level	4	9,954.12	75.57	<0.001
3.2 + Aggregation type	5	9,956.06	0.05	0.81
4.3—Random factor (Participants)	4	10,094.57	140.50	<0.001

*The participant was included as a random factor*.

**Table 4:** Parameter estimates in the linear mixed model for landscape preference rating as a function of aggregation type.

**Predictor**	***B***	***SE***	***T***	***P***
(Intercept)	4.15	0.10	39.57	<0.001
Aggregation level (low)	0.59	0.06	8.75	<0.001
Aggregation type (Interspersion)	−0.02	0.07	−0.24	0.80

### Fixation Time

**Figure 6:**
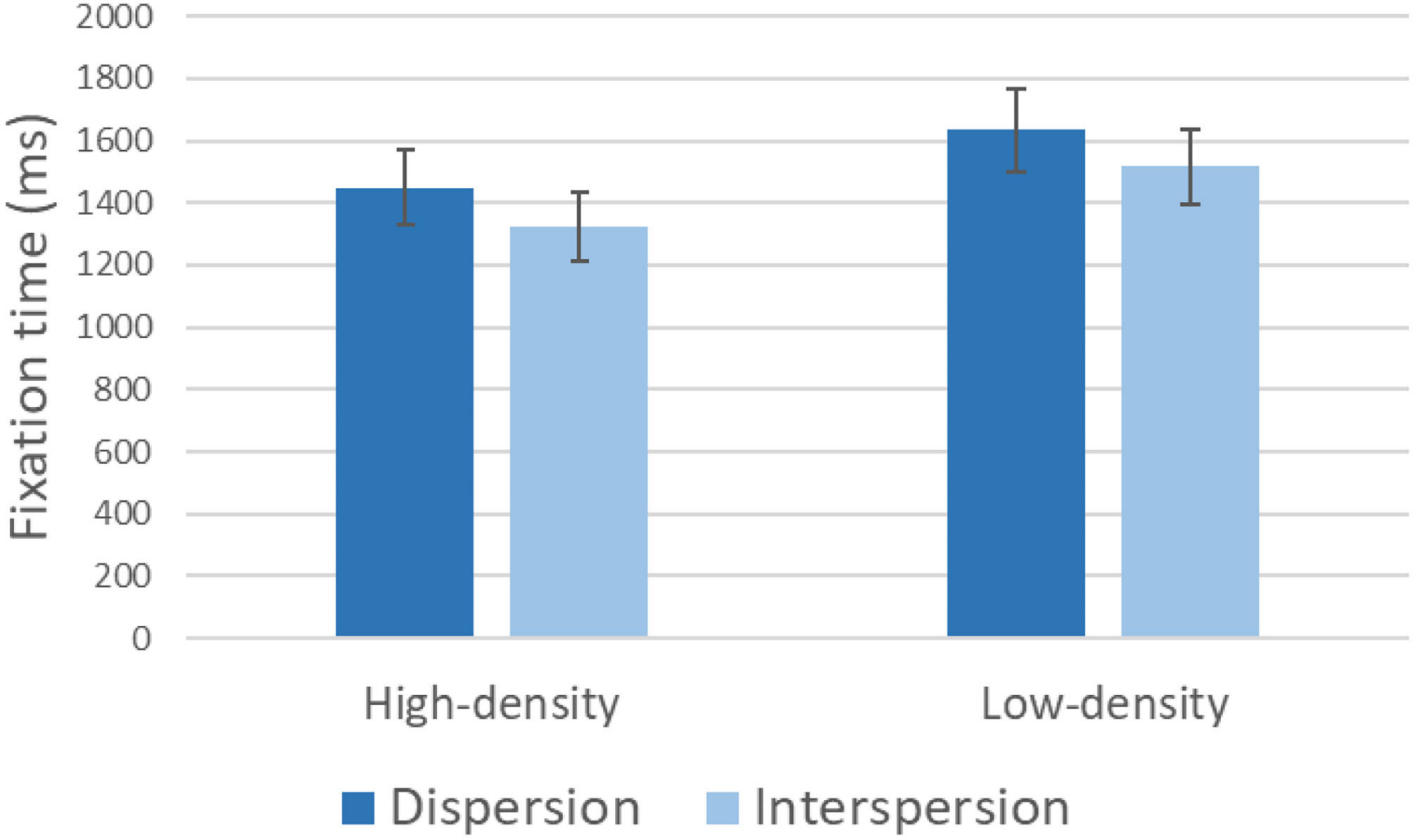
Cumulative fixation time as a function of aggregation level and aggregation type.

**Figure 7:**
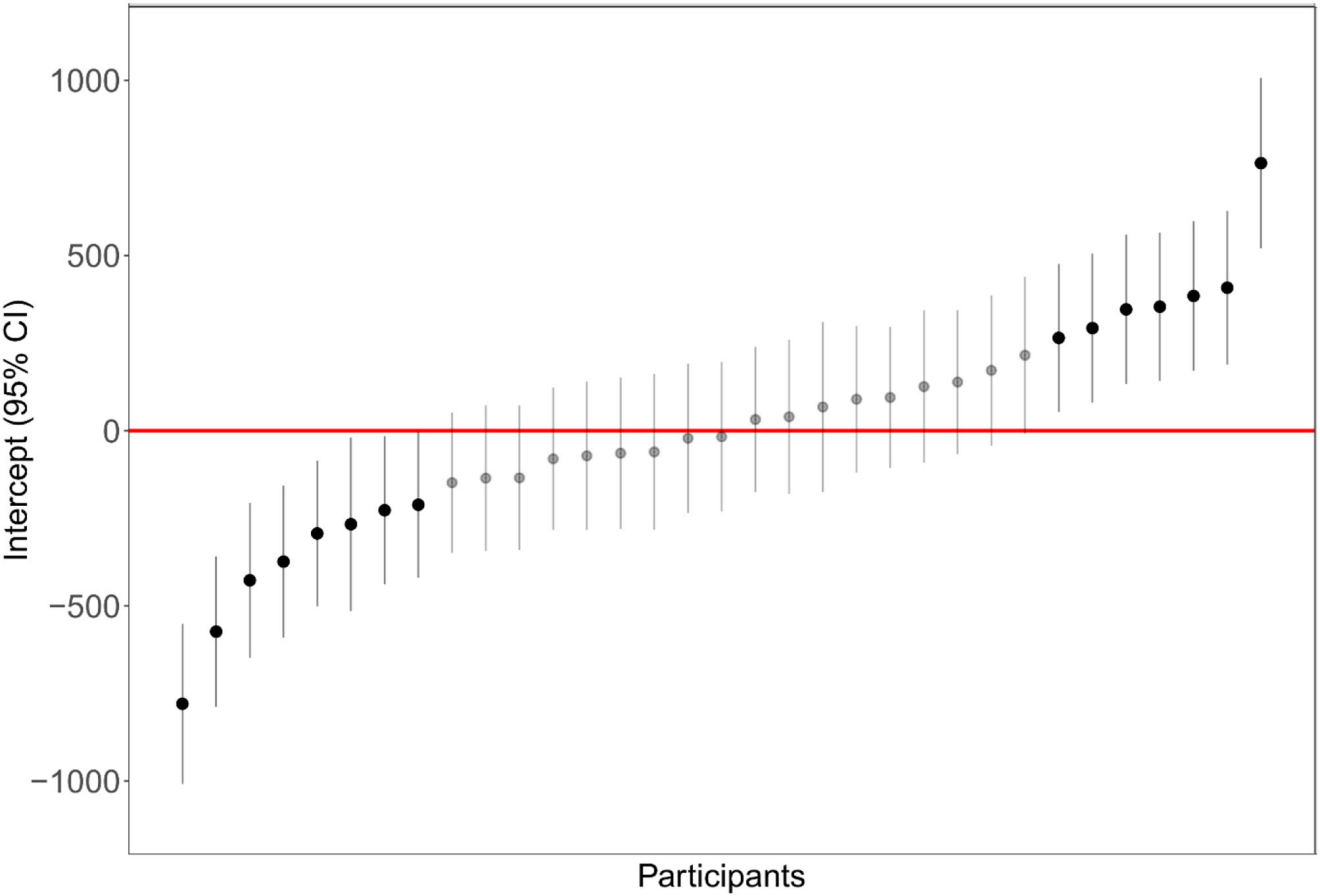
Distribution of participants' intercept (random effect) in the linear mixed model testing cumulative fixation time as a function of aggregation level and aggregation type.

A linear mixed model evaluated the effect of aggregation level and aggregation type (dispersion vs. interspersion) on the total fixation time to the stimulus. The participant was included as a random factor. The results of the model are reported in Table 5, and Table 6 reported the parameter estimates of the linear model. The total fixation time was significantly influenced by both levels of aggregation level and aggregation type. The random factor (participants) was also significant. The total fixation time was 1,577 ms (*SE* = 60.3), 95% CI (1,263, 1,509) in the low-aggregation conditions and 1,383 ms (*SE* = 60.3), 95% CI (1,454, 1,700) in the high-aggregation condition (Figure 6). In the dispersion condition, the mean fixation time was 1,540 ms (*SE* = 59.6), 95% CI (1,418, 1,661), whereas in the interspersion condition, the mean fixation time was 1,424 ms (*SE* = 61.1), 95% CI (1,299, 1,548) (Figure 6). The distribution of intercepts for the random effect participants is shown in Figure 7.

**Table 5:** Linear mixed model results for total fixation time as a function of aggregation level (low-high) and aggregation type (dispersion-interspersion).

**Model**	**df**	**AIC**	**χ^2^**	***p***
1. Intercept	3	41,481.36		
2.1 + Aggregation level	4	41,453.12	30.23	<0.001
3.2 + Aggregation type	5	41,444.12	11	<0.001
4.3—Random factor (Participants)	4	41,658.10	215.98	<0.001

*The participant was included as random factor*.

**Table 6:** Parameter estimates in the linear mixed model for landscape preference rating as a function of aggregation level and aggregation type.

**Predictor**	***B***	***SE***	***T***	***P***
(Intercept)	1,444.19	61.10	23.33	<0.001
Aggregation level (low)	191.04	34.59	5.52	<0.001
Aggregation type (interspersion)	−116.21	35.01	−3.31	<0.001

### First Fixation

The first fixation was on the high-aggregation stimulus in 624 cases and on the low-aggregation stimulus in 696 cases. The proportion test without continuity correction was significant: χ^2^ = 3.92, *p* = 0.04. Table 7 shows first fixation frequencies as a function of aggregation level and aggregation type.

**Table 7:** Frequencies of first fixation target as a function of aggregation type and aggregation level.

**Aggregation**	**High-aggregation**	**Low-aggregation**
Dispersion	353 (46.5%)	406 (53.5%)
Interspersion	271 (48.3%)	290 (51.7%)

## Discussion

The study aimed to test the effect of vegetation aggregation on landscape preference. Aggregation varied according to two criteria: dispersion and interspersion. In the first case, the distance between plants was manipulated, while in the second case, the degree of intermixing/separation between plants of two different species varied systematically. The results showed a preference for landscapes with a lower level of vegetation aggregation, considering either dispersion or interspersion. This result emerged coherently across the self-report and behavioral (i.e., eye movements) measures considered in the study.

The preference for landscapes with plants arranged in a high dispersion layout could be explained by the fact that this layout increase the perception of landscape naturalness and lack of formality. According to this perspective, the level of plant dispersion could affect the perception of naturalness, which is usually associated with a higher degree of preference in landscapes (Kaplan and Kaplan, 1989; Purcell et al., 1994). Also, the effect of preference for a high level of vegetation interspersion could be explained by the higher naturalness perception of this layout in comparison to a layout in which plants of different species are separated in macro-patches. In the case of high interspersion, plants of different species/variety were more intermixed than they were in the case of low-interspersion layout, where the different plants tended to be more segregated. Interspersion also affected the mean patch size at which the landscape could be perceptually segregated, and a small mean patch size is associated with a higher landscape preference (Di Cristofaro et al., 2020).

The study confirms the results of van Zanten et al. (2016), who found a preference for diversification in rural landscapes. A decrease in plant intermixing tends to result in the homogenization of the agricultural landscape and a negative impact on its aesthetics and recreational value. The results are also in agreement with those of Qiu et al. (2013), who found that half-open green areas tend to be preferred over areas with more complex and aggregated vegetation.

An alternative explanation, especially referred to the preference for a high-dispersion layout, could be that this design results in increased visual balance, with plants arranged in the available space with a better level of uniformity. Indeed many studies on experimental aesthetics have highlighted a clear preference for visual balance (Arnheim, 1974; Banich et al., 1989; McManus et al., 1993; Locher, 1996; Palmer et al., 2013). Furthermore, the aggregation has a direct consequence on the spatial frequency of a landscape, a factor that was shown to have a significant role in determining the attractiveness of perceptual stimuli (e.g., Ogawa and Motoyoshi, 2020). Specifically, aggregation tends to reduce the spatial frequency, creating larger patches and reducing intermixing.

It is essential to consider that a level of aggregation is always referred to as a specific scale of the territory (Forman and Wilson, 1995). In this study, the landscape scale was related to that of residential gardens, as this is a scale of frequent interaction of individuals with green spaces. Focusing on gardens was an additional element of the originality of the study because the majority of previous studies were focused on large-scale landscapes (e.g., Sertel et al., 2018; Di Cristofaro et al., 2020). The scientific question of whether landscape scale plays a critical role in altering the factors that contribute to a participant's preference is open, a question that has never been addressed systematically in previous research. For example, it can be suggested that the larger the landscape scale, the easier the perceptual segmentation in homogenous patches, with a detrimental effect on the perception of distinctive, fine-grained features.

The parameters of fixation count and fixation cumulative time had a substantial concordance with self-reported judgments of preference, showing the same pattern of results. This is in agreement with the studies of Shimojo et al. (2003), Simion and Shimojo (2006, 2007), and Schweikert et al. (2016), who used the same paradigm of presenting dual images, specifically faces, and comparing self-reported preference with looking behavior indexes. They found that gaze was shown to be biased toward the face that was evaluated as more attractive. Similarly, Glaholt and Reingold (2009) demonstrated a robust bias toward a preferred art image in both a two-alternative free-viewing condition and an eight-alternative free-viewing condition, considering the gaze duration and gaze frequency. Interestingly, this study found that the target of the first fixation was biased toward the landscape that was lately evaluated as more attractive also if the power and effect size of this result was rather small.

Dispersion and interspersion were manipulated in this study only on one level. Additional research is needed to model landscape preference as a function of a broader range of plant distances and plant intermixing. All the linear mixed models highlighted the importance of the random factor “participant” in explaining the variance (Figures 4, 5, 7). This is of relevance because the general preference for non-aggregated plant layouts was mitigated by an important factor of individual differences. Considering preference ratings, a percentage of 14.28% of participants, for example, always preferred the low-aggregation display, and a percentage of 12.5% of participants always preferred the high-aggregation display. Rather one-quarter of participants showed a clear polarization of judgment, which was confirmed considering the number of fixations, and the total time of fixations. Further research is needed to clarify the underlying factors that modulate these individual differences.

The results of this study are of importance not only for a general theory of scenic beauty perception in landscapes but also for garden and landscape designers who can apply the results of this study, considering greenery dispersion and interspersion as important factors for the aesthetical value of their projects.

The participants in the study were university students, and future studies should test if the results are generalizable to a population of experts in landscape design. Previous research (e.g., Tveit, 2009; Hägerhäll et al., 2018) has shown that expertise could play a significant role in landscape assessment. It would also be interesting to study if the degree of familiarity and fruition with gardens and parks could affect the preferences for green dispersion and interspersion, as well as whether age could play a significant role, using a sample of older participants.

## Data Availability Statement

The raw data supporting the conclusions of this article will be made available by the authors, without undue reservation.

## Ethics Statement

The studies involving human participants were reviewed and approved by Bioethics Committee Alma Mater Studiorum University of Bologna. The patients/participants provided their written informed consent to participate in this study.

## Author Contributions

MC has conceived the study, conducted the data collection and analysis, and written the manuscript.

## Conflict of Interest

The author declares that the research was conducted in the absence of any commercial or financial relationships that could be construed as a potential conflict of interest.

## Publisher's Note

All claims expressed in this article are solely those of the authors and do not necessarily represent those of their affiliated organizations, or those of the publisher, the editors and the reviewers. Any product that may be evaluated in this article, or claim that may be made by its manufacturer, is not guaranteed or endorsed by the publisher.

## Supplementary Material

Supplementary Images created with Realtime Landscaping Architect. Reproduced with permission.
