# Supplementary figures and images for: Vegetation Dispersion, Interspersion, and Landscape Preference
Source: Front Psychol. 2022 May 20;13:771543. doi: 10.3389/fpsyg.2022.771543 (PMC9165701; doi:10.3389/fpsyg.2022.771543)

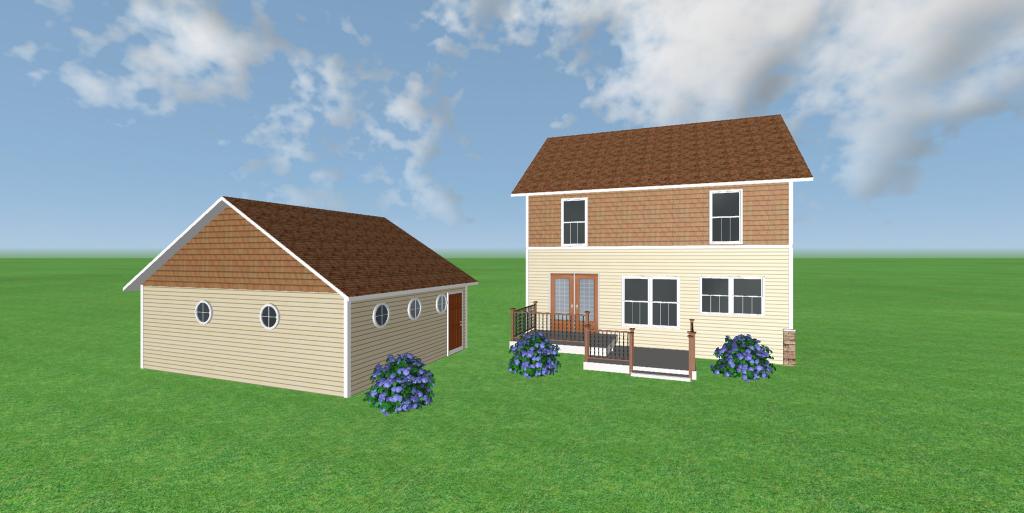

Supplement: Supplementary file 1 [file Data_Sheet_1.ZIP › dispersion high 1.png]

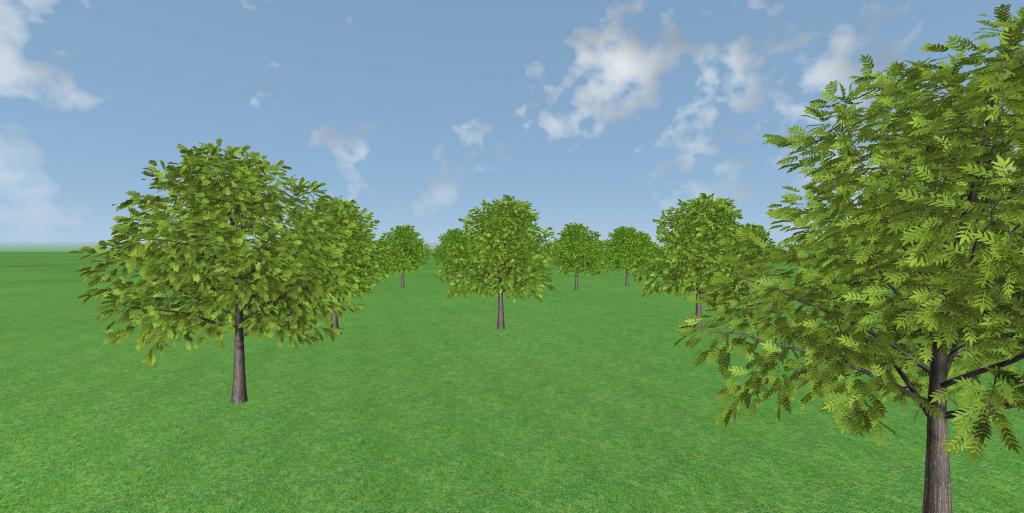

Supplement: Supplementary file 1 [file Data_Sheet_1.ZIP › dispersion high 11.png]

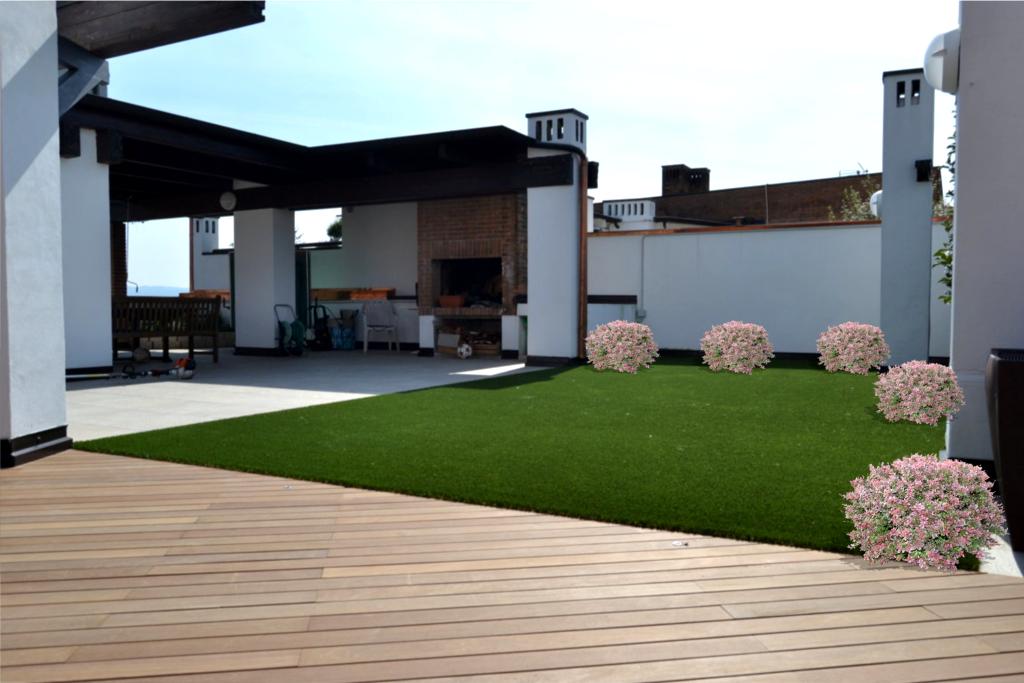

Supplement: Supplementary file 1 [file Data_Sheet_1.ZIP › dispersion high 21.png]

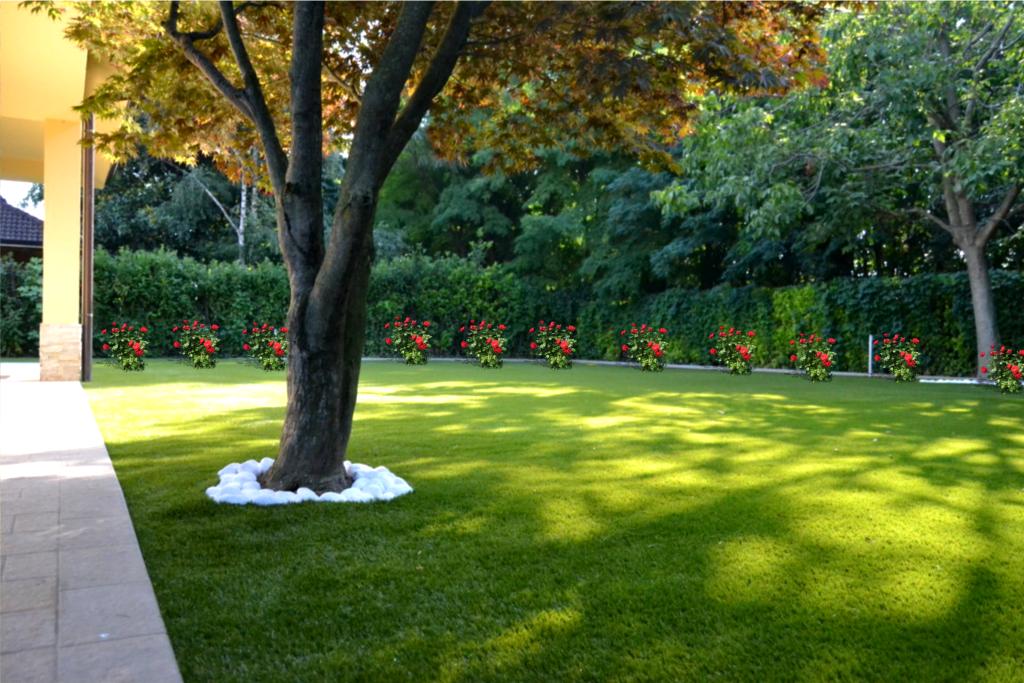

Supplement: Supplementary file 1 [file Data_Sheet_1.ZIP › dispersion high 22.png]

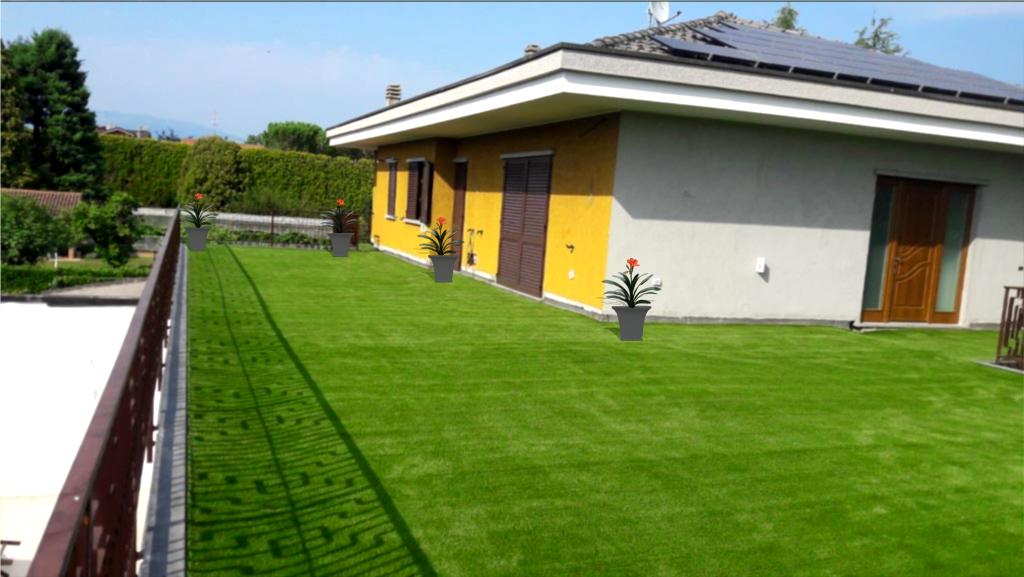

Supplement: Supplementary file 1 [file Data_Sheet_1.ZIP › dispersion high 23.png]

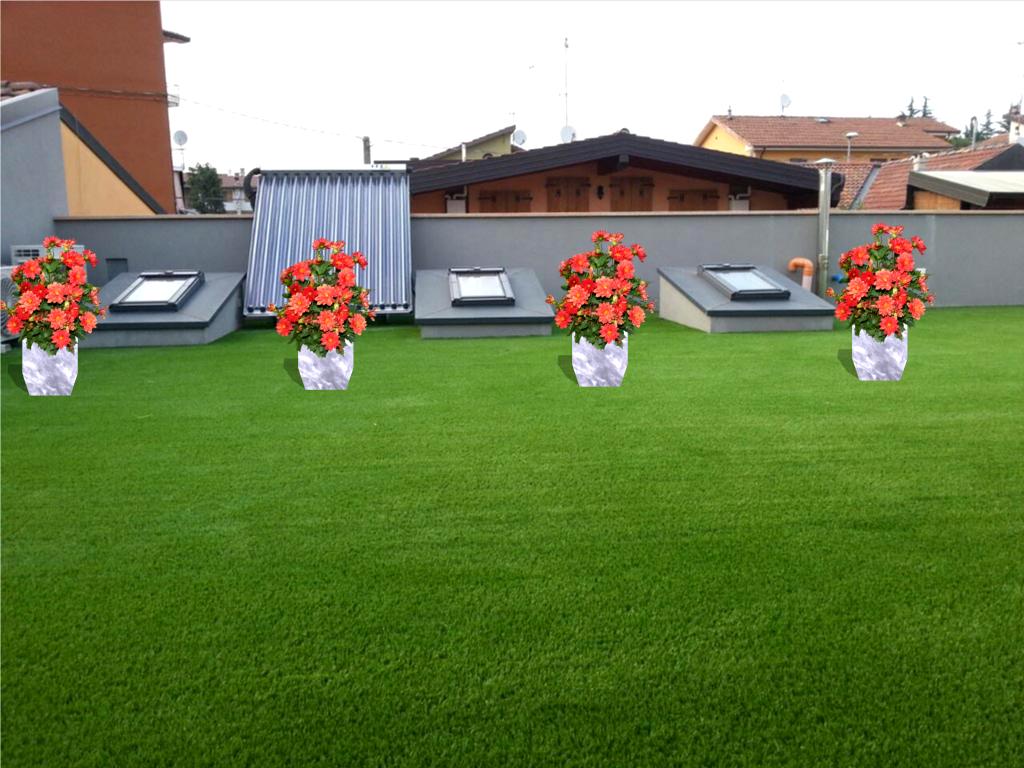

Supplement: Supplementary file 1 [file Data_Sheet_1.ZIP › dispersion high 24.png]

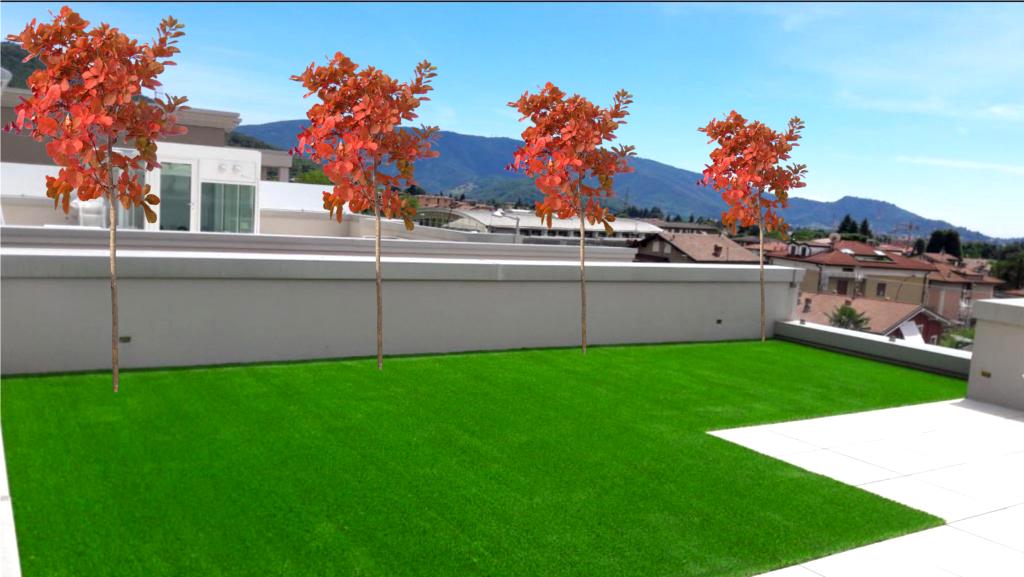

Supplement: Supplementary file 1 [file Data_Sheet_1.ZIP › dispersion high 25.png]

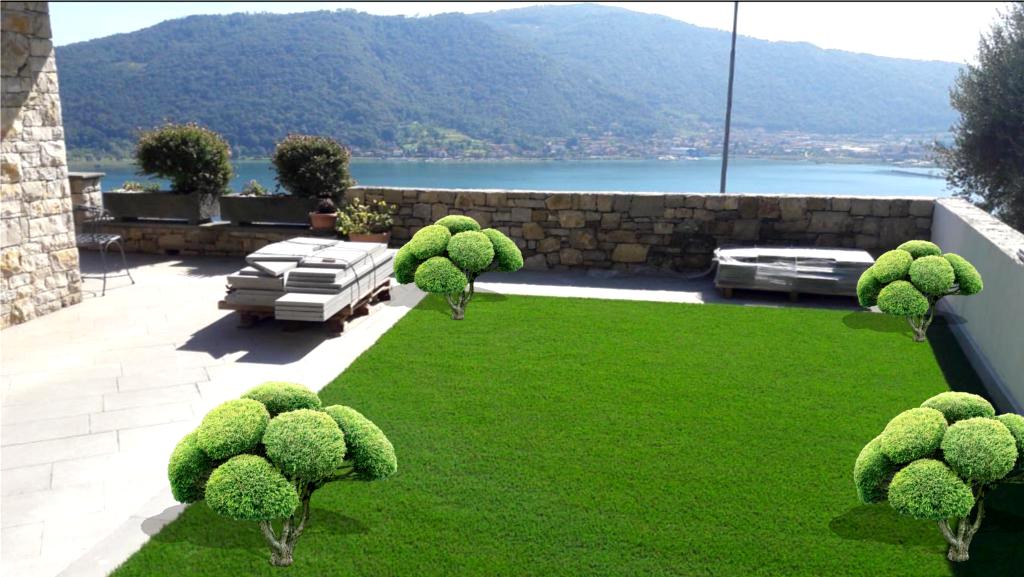

Supplement: Supplementary file 1 [file Data_Sheet_1.ZIP › dispersion high 26.png]

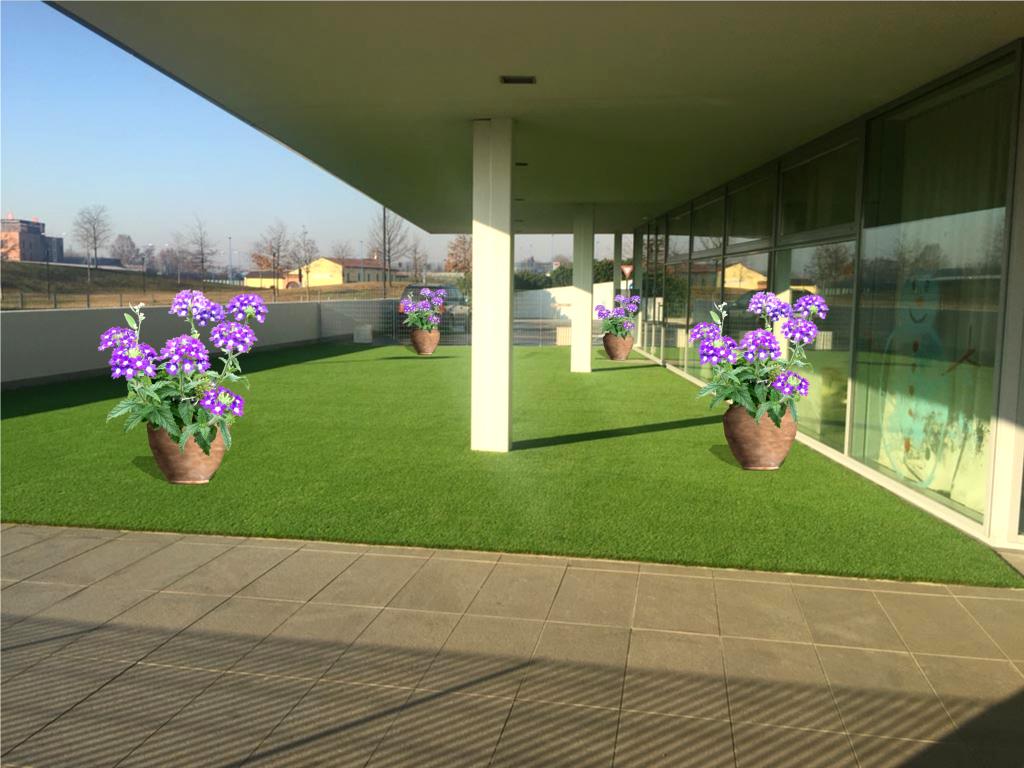

Supplement: Supplementary file 1 [file Data_Sheet_1.ZIP › dispersion high 27.png]

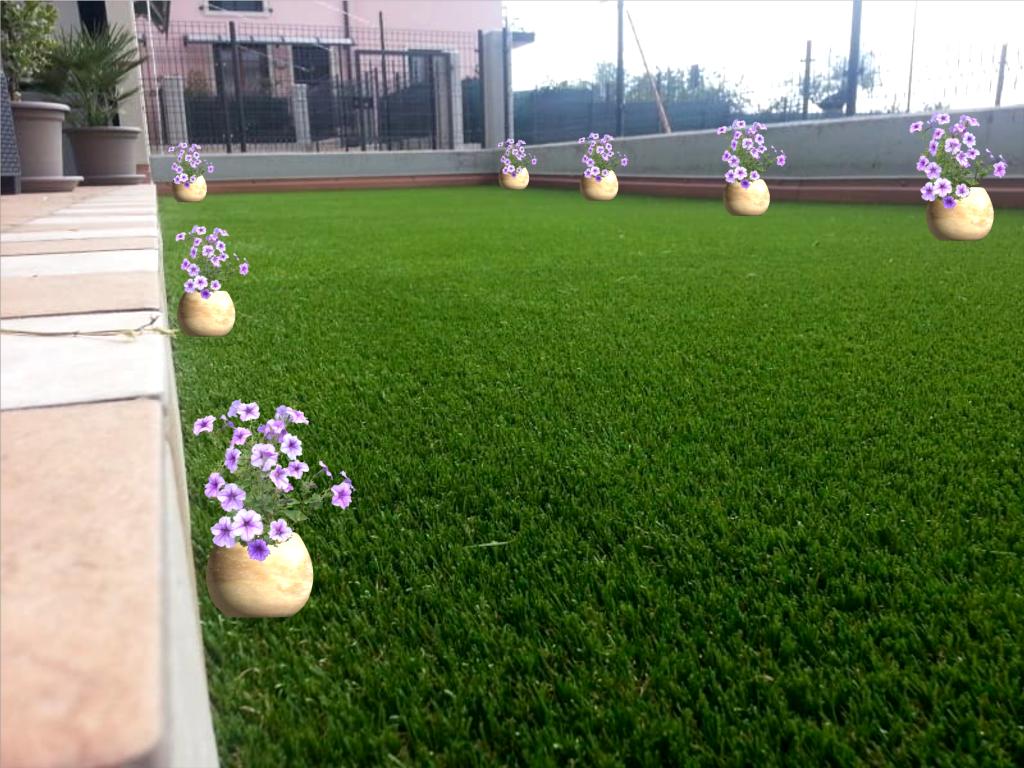

Supplement: Supplementary file 1 [file Data_Sheet_1.ZIP › dispersion high 28.png]

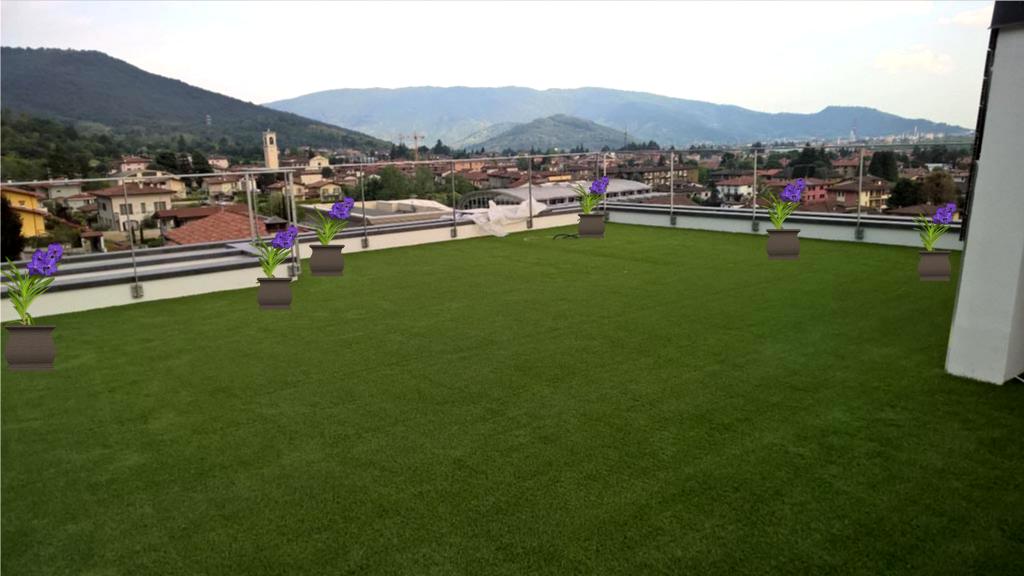

Supplement: Supplementary file 1 [file Data_Sheet_1.ZIP › dispersion high 29.png]

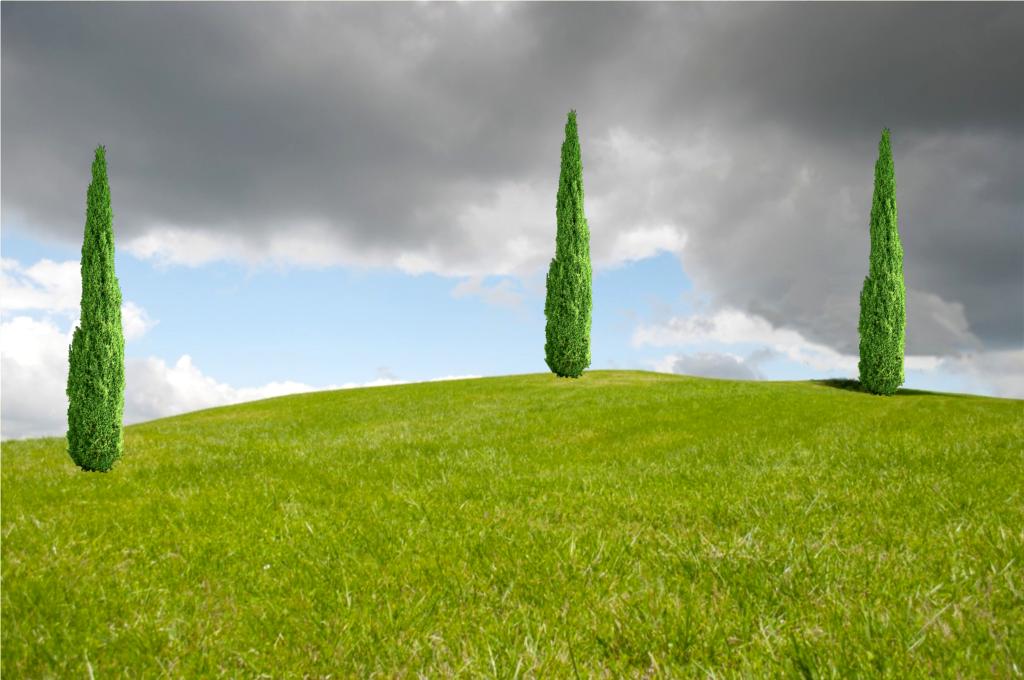

Supplement: Supplementary file 1 [file Data_Sheet_1.ZIP › dispersion high 3.png]

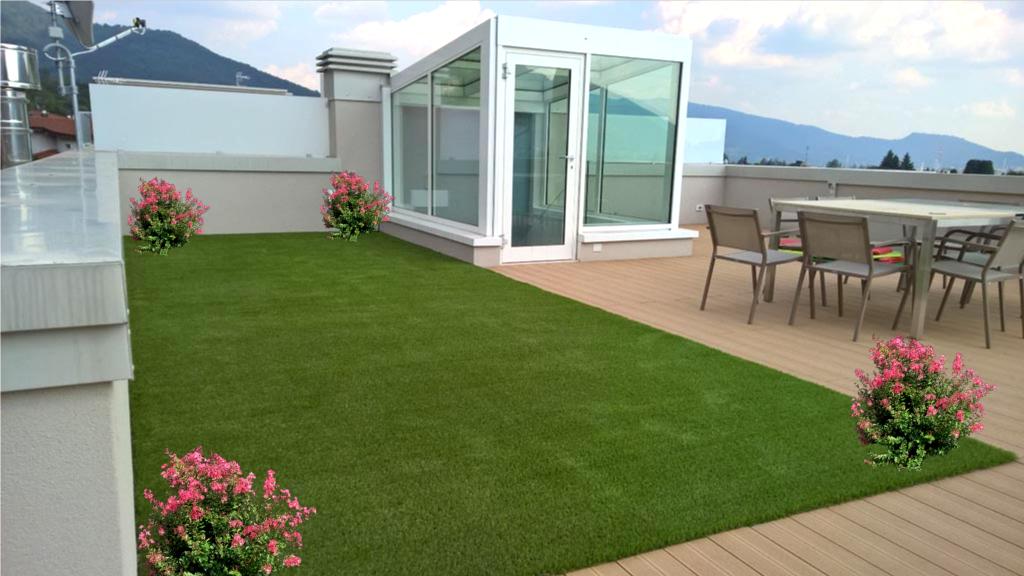

Supplement: Supplementary file 1 [file Data_Sheet_1.ZIP › dispersion high 30.png]

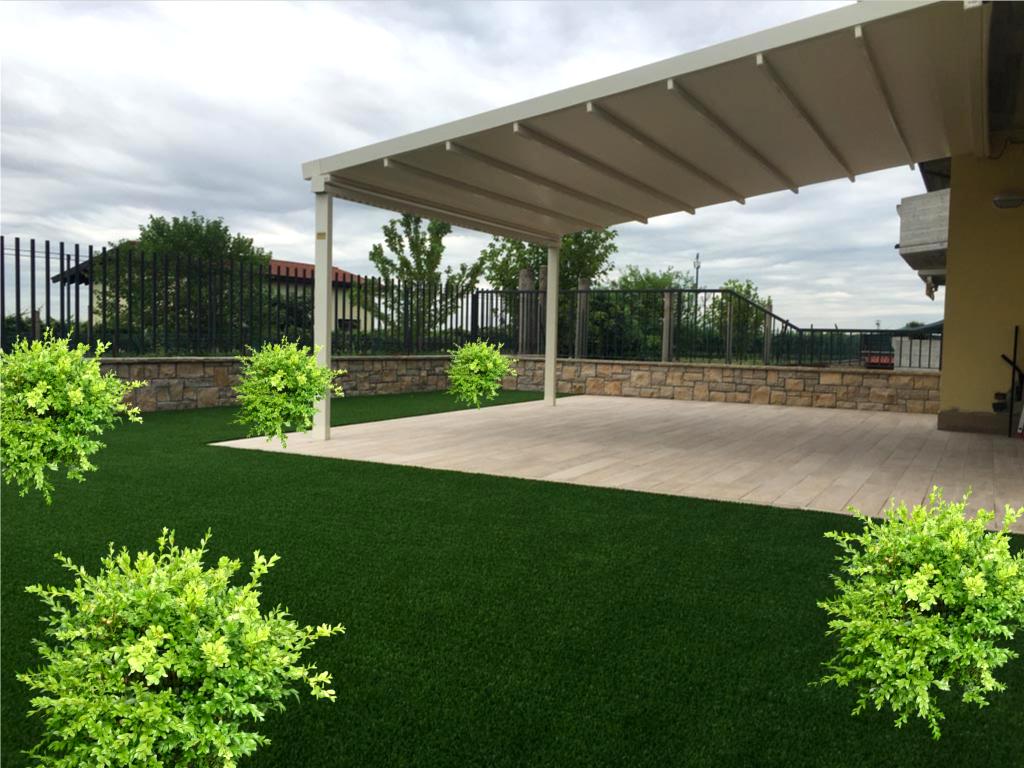

Supplement: Supplementary file 1 [file Data_Sheet_1.ZIP › dispersion high 31.png]

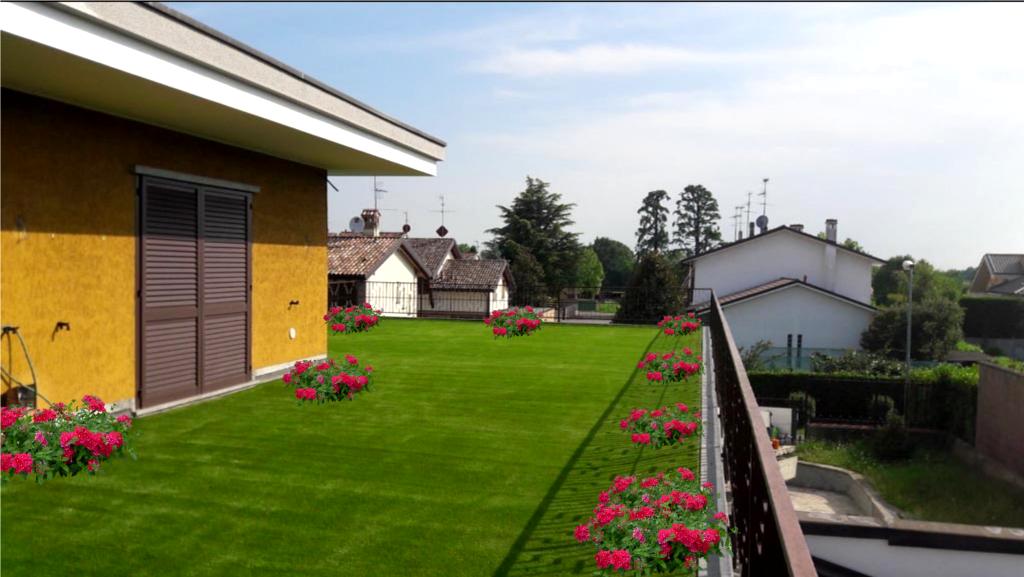

Supplement: Supplementary file 1 [file Data_Sheet_1.ZIP › dispersion high 32.png]

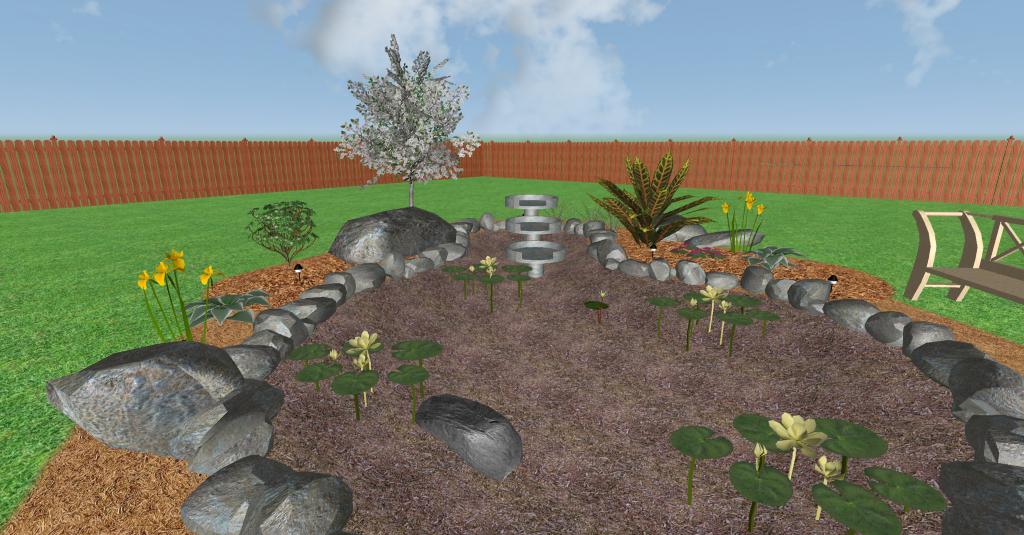

Supplement: Supplementary file 1 [file Data_Sheet_1.ZIP › dispersion high 33.png]

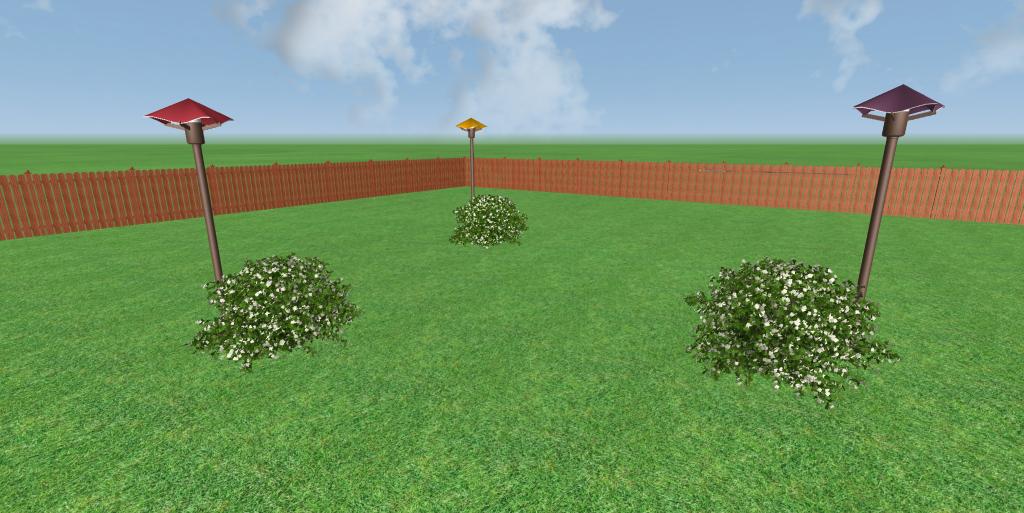

Supplement: Supplementary file 1 [file Data_Sheet_1.ZIP › dispersion high 34.png]

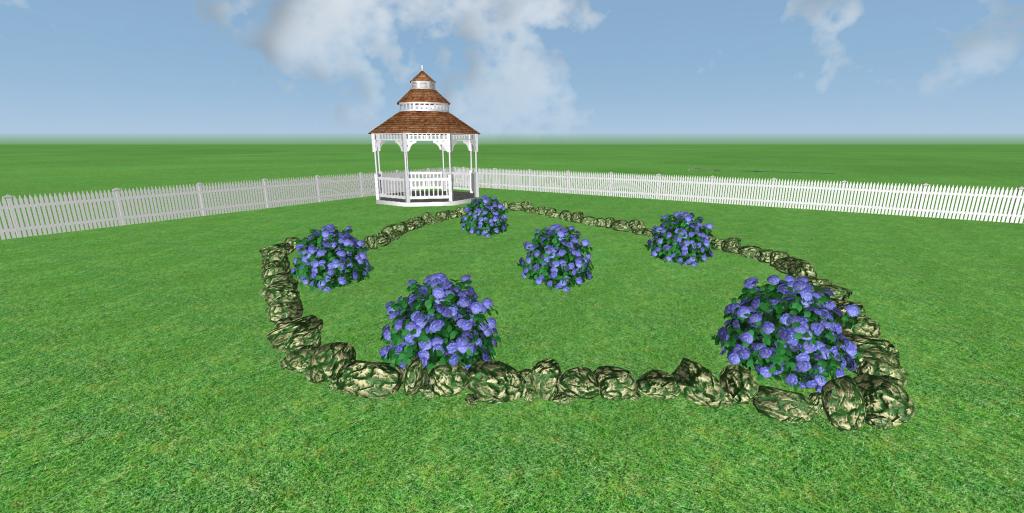

Supplement: Supplementary file 1 [file Data_Sheet_1.ZIP › dispersion high 35.png]

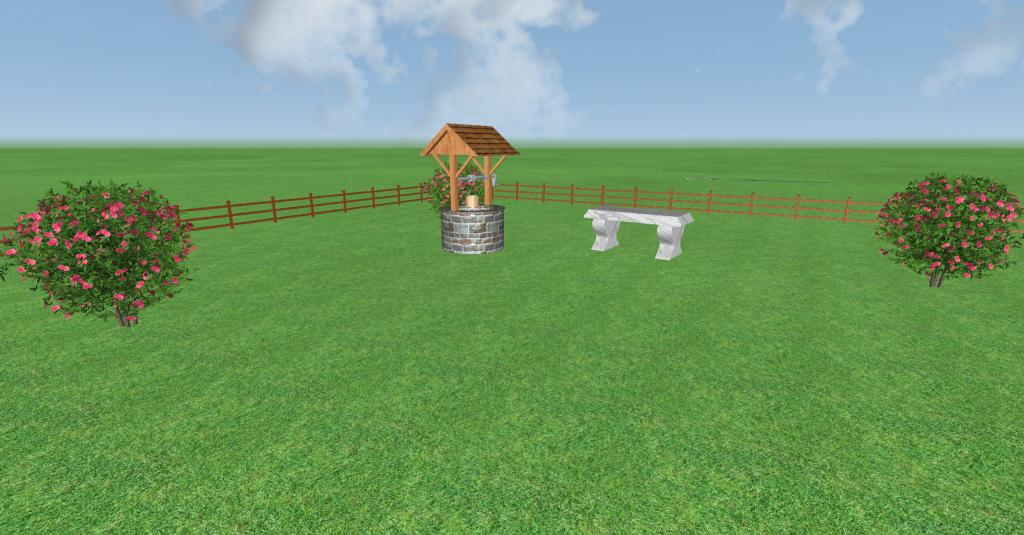

Supplement: Supplementary file 1 [file Data_Sheet_1.ZIP › dispersion high 36.png]

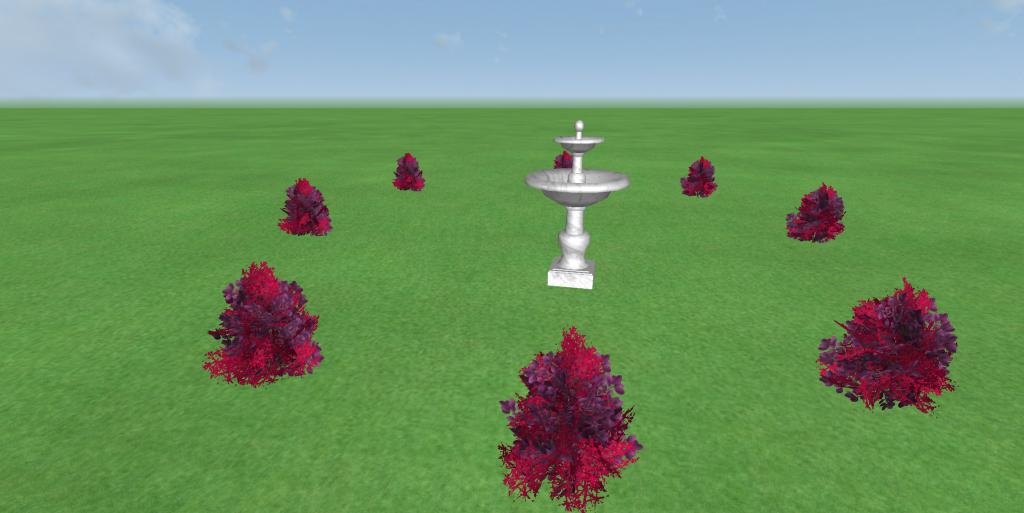

Supplement: Supplementary file 1 [file Data_Sheet_1.ZIP › dispersion high 37.png]

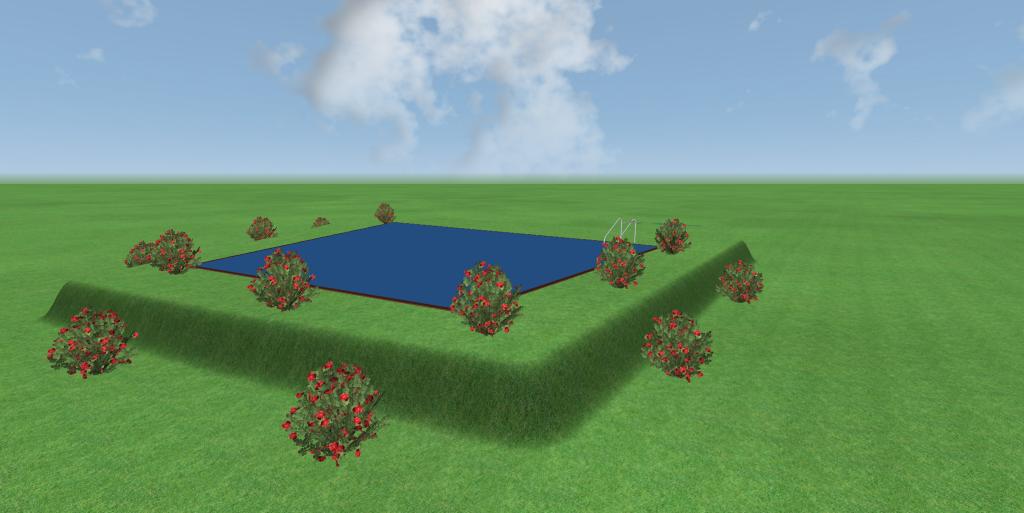

Supplement: Supplementary file 1 [file Data_Sheet_1.ZIP › dispersion high 38.png]

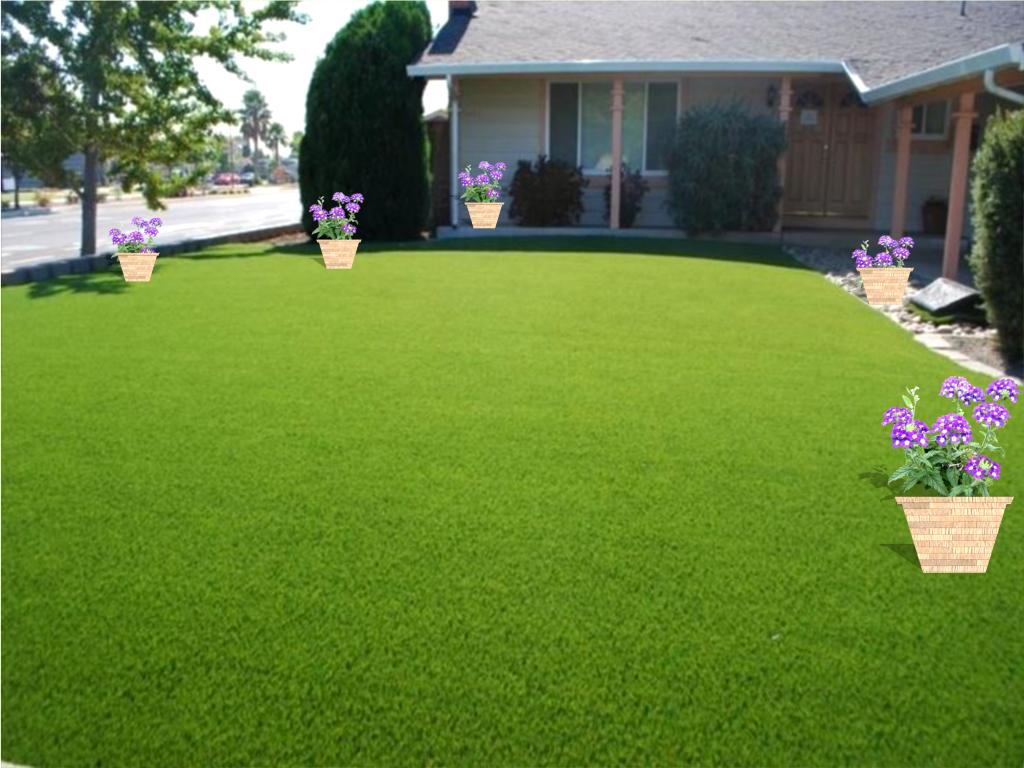

Supplement: Supplementary file 1 [file Data_Sheet_1.ZIP › dispersion high 39.png]

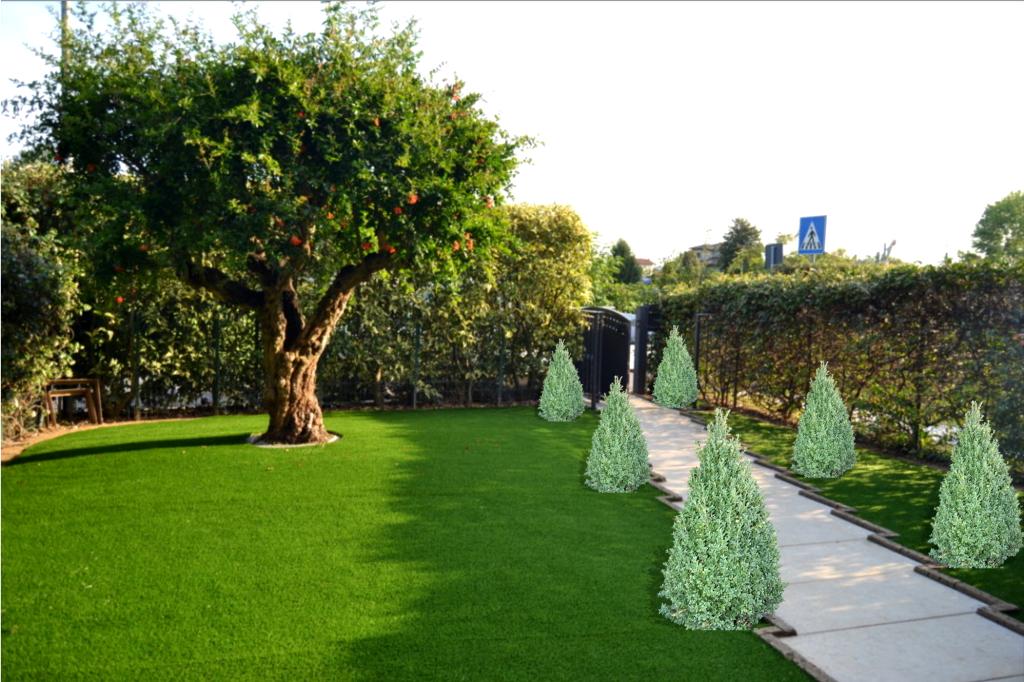

Supplement: Supplementary file 1 [file Data_Sheet_1.ZIP › dispersion high 40.png]

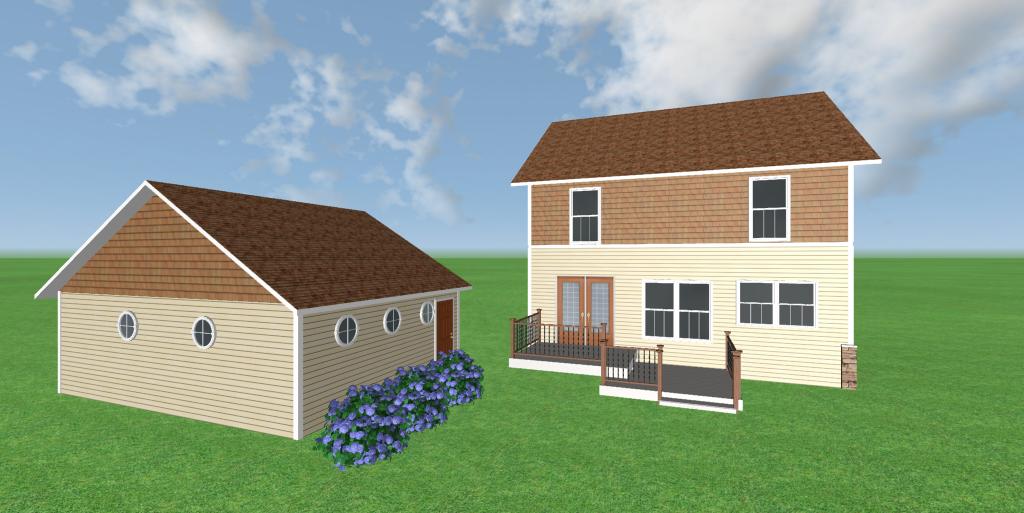

Supplement: Supplementary file 1 [file Data_Sheet_1.ZIP › dispersion low 1.png]

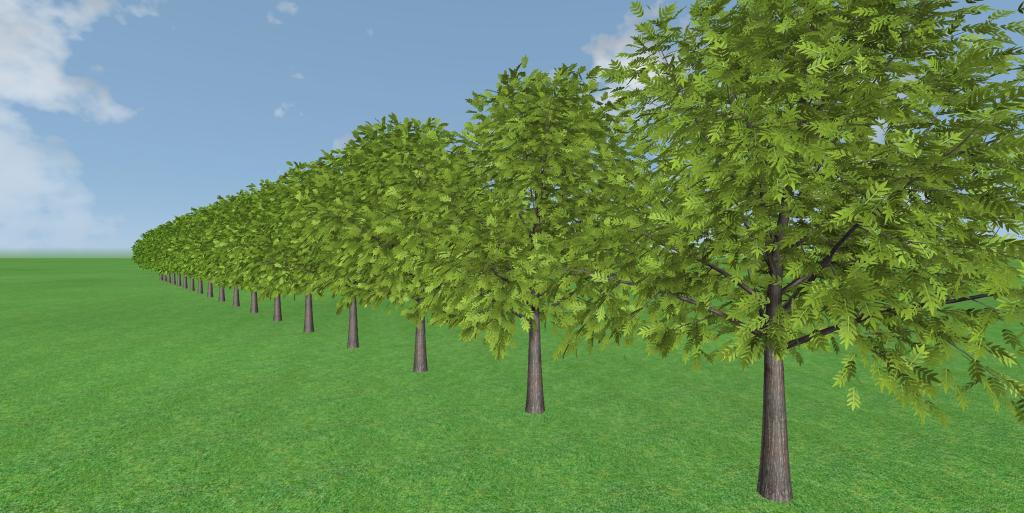

Supplement: Supplementary file 1 [file Data_Sheet_1.ZIP › dispersion low 11.png]

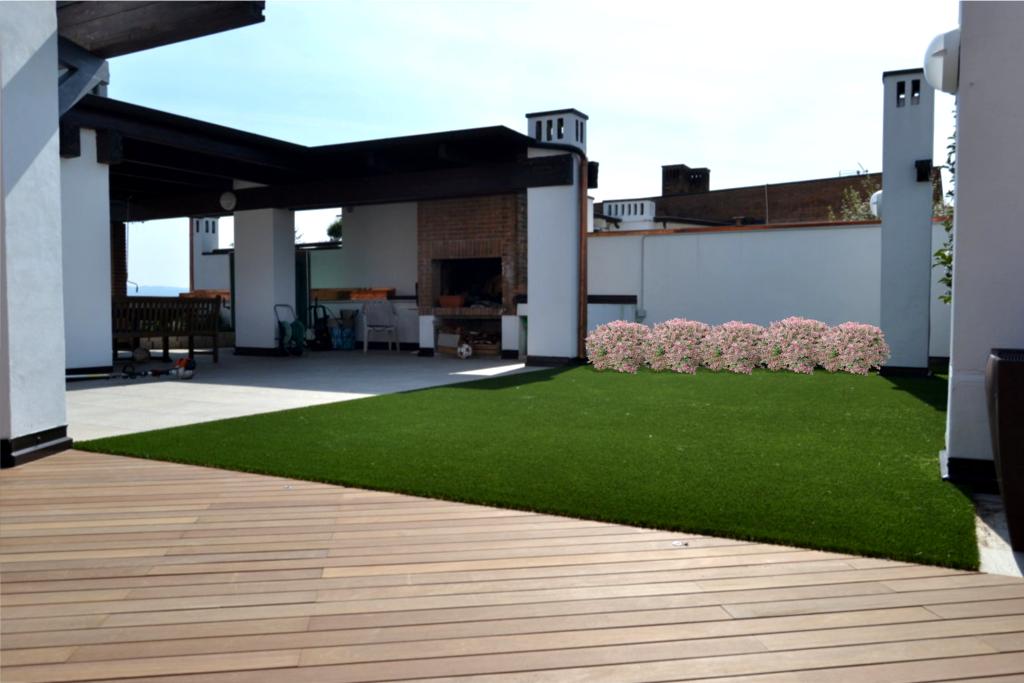

Supplement: Supplementary file 1 [file Data_Sheet_1.ZIP › dispersion low 21.png]

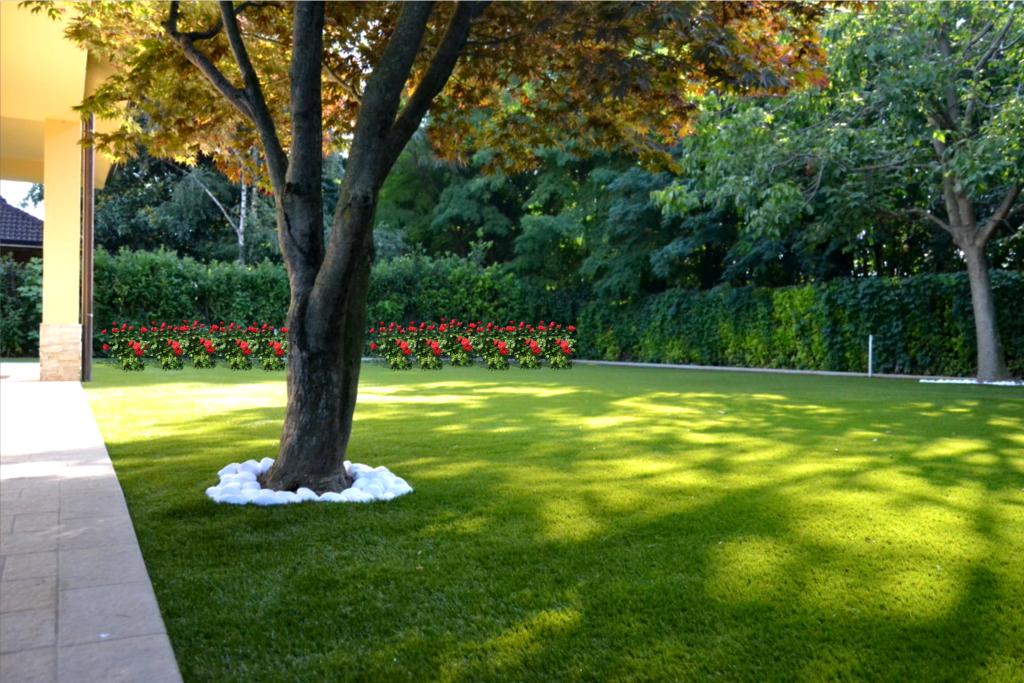

Supplement: Supplementary file 1 [file Data_Sheet_1.ZIP › dispersion low 22.png]

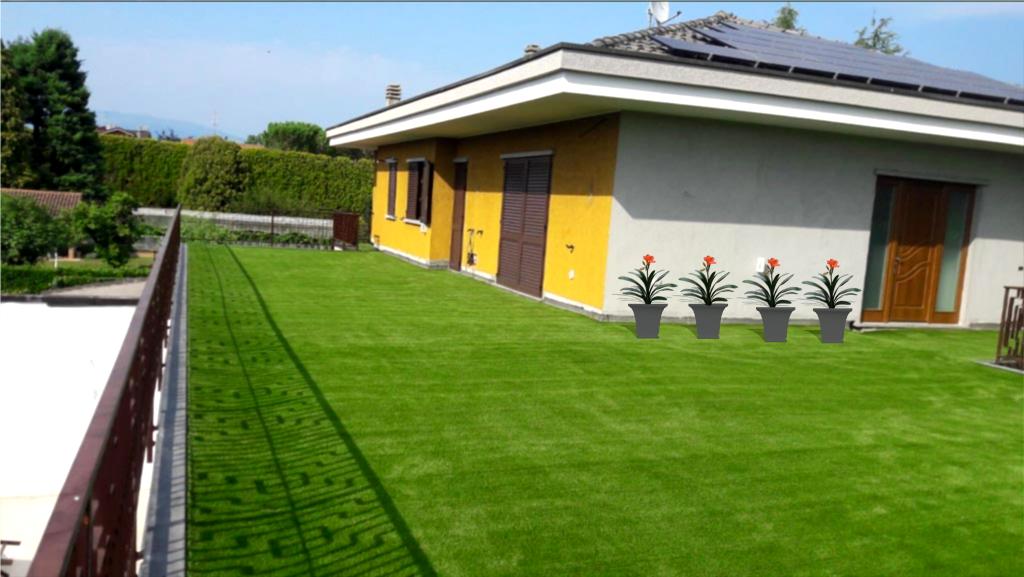

Supplement: Supplementary file 1 [file Data_Sheet_1.ZIP › dispersion low 23.png]

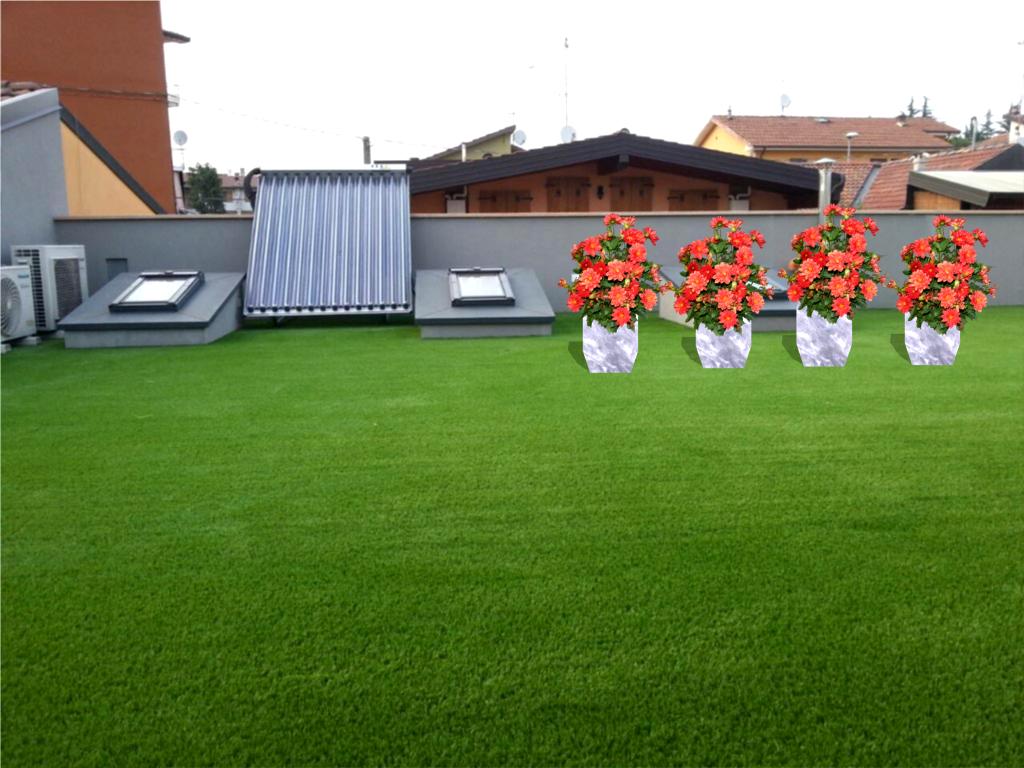

Supplement: Supplementary file 1 [file Data_Sheet_1.ZIP › dispersion low 24.png]

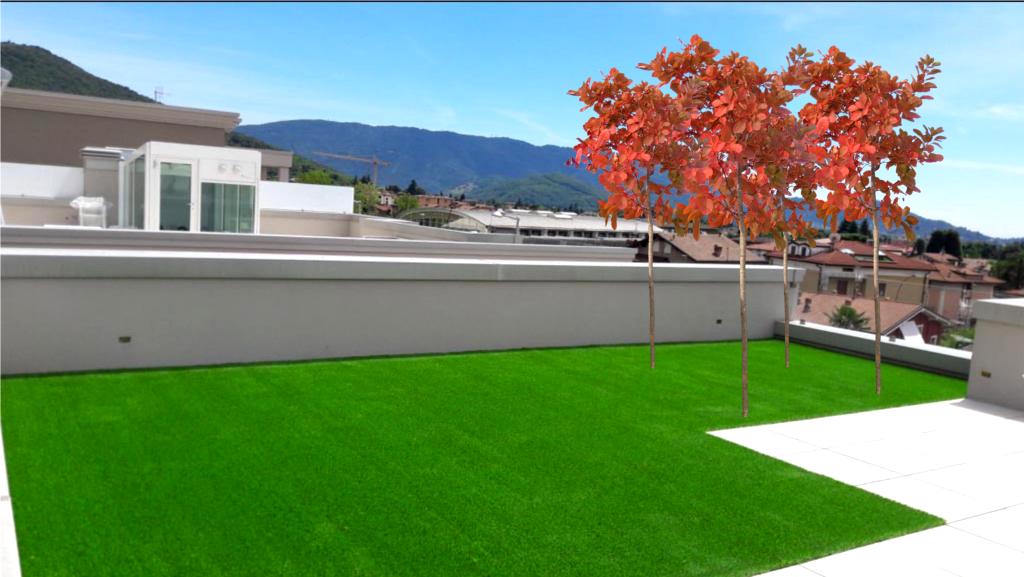

Supplement: Supplementary file 1 [file Data_Sheet_1.ZIP › dispersion low 25.png]

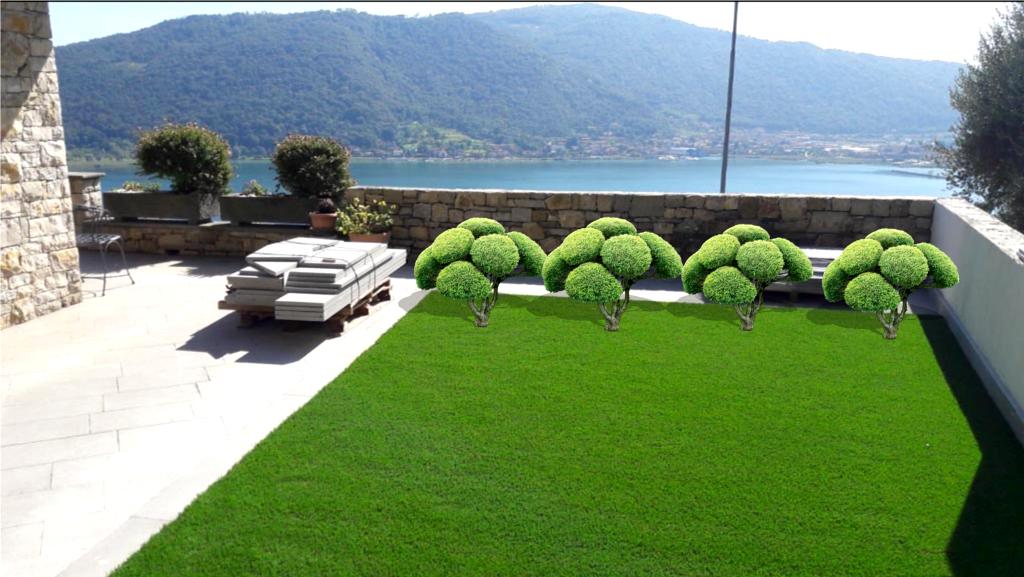

Supplement: Supplementary file 1 [file Data_Sheet_1.ZIP › dispersion low 26.png]

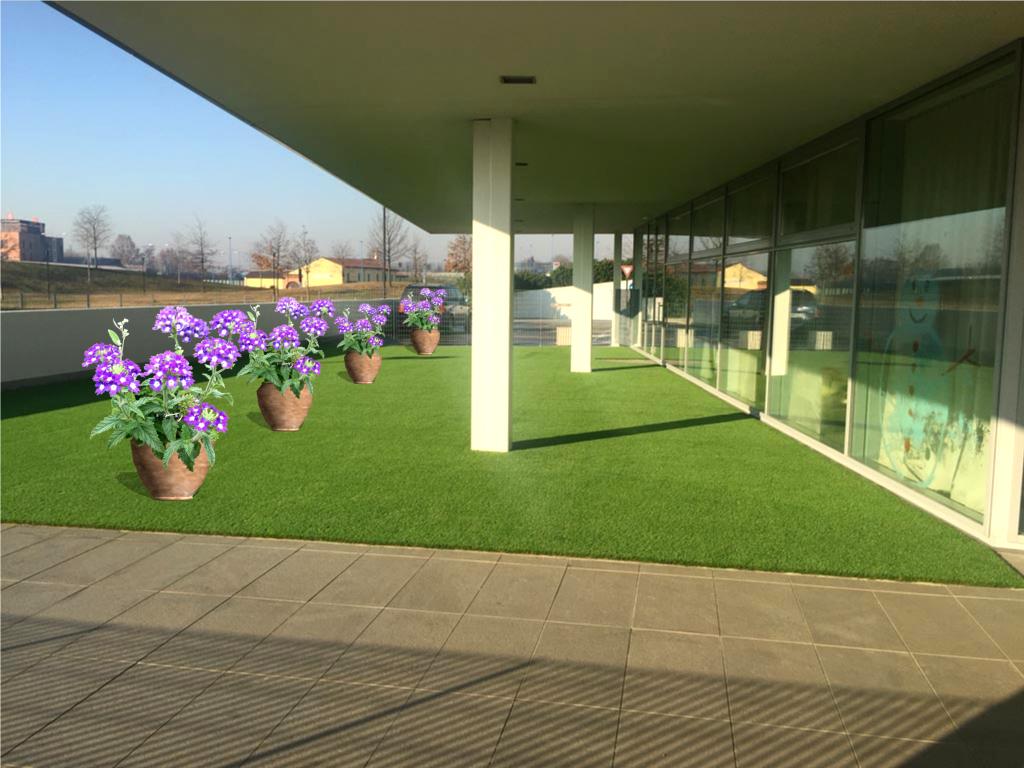

Supplement: Supplementary file 1 [file Data_Sheet_1.ZIP › dispersion low 27.png]

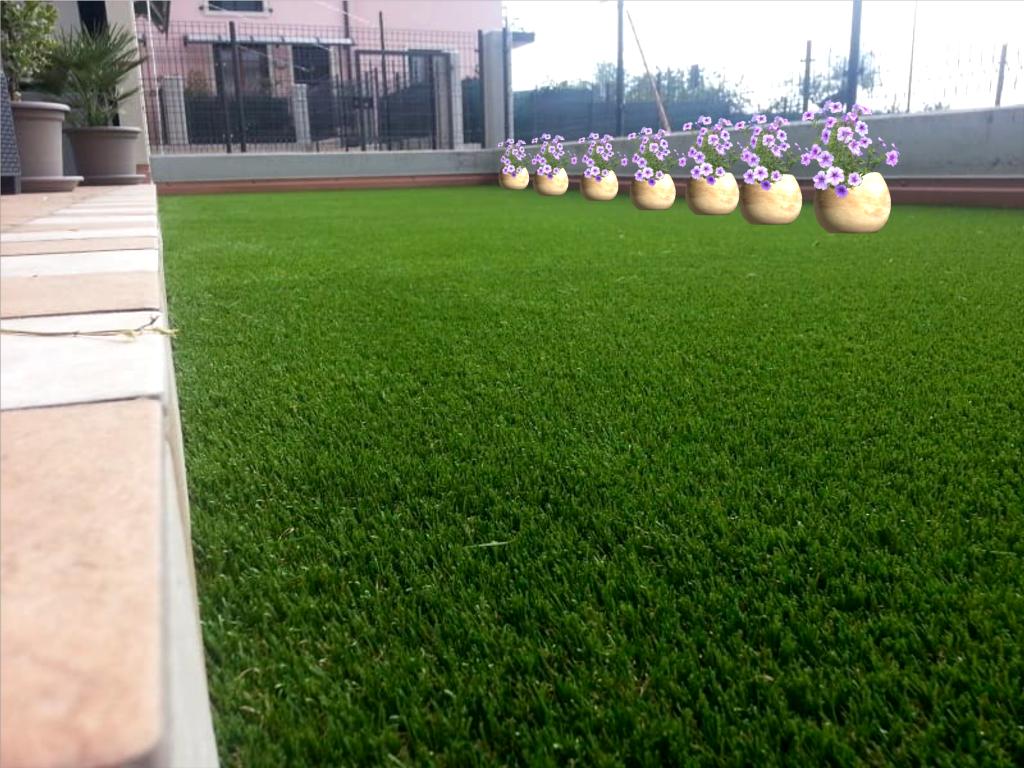

Supplement: Supplementary file 1 [file Data_Sheet_1.ZIP › dispersion low 28.png]

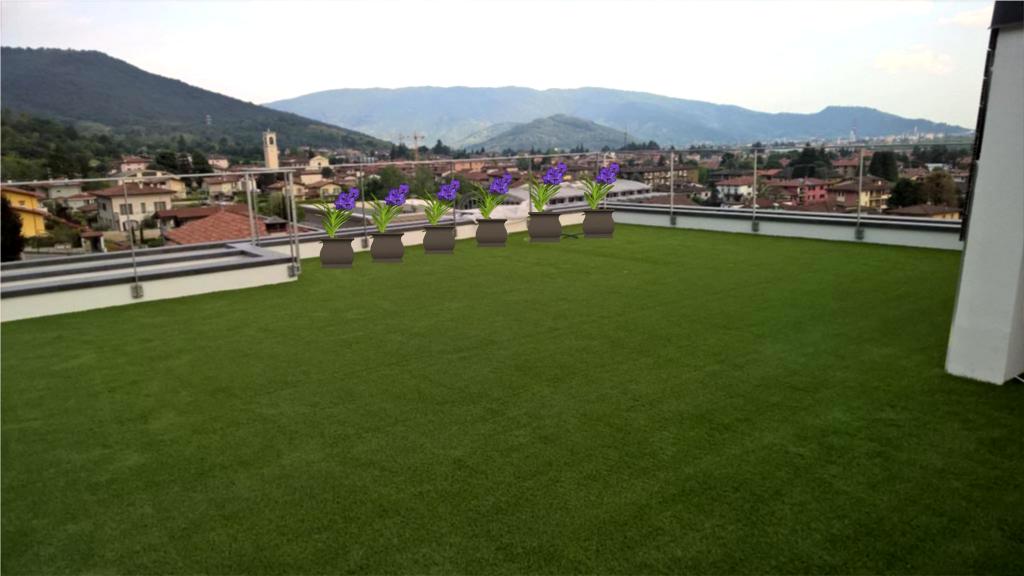

Supplement: Supplementary file 1 [file Data_Sheet_1.ZIP › dispersion low 29.png]

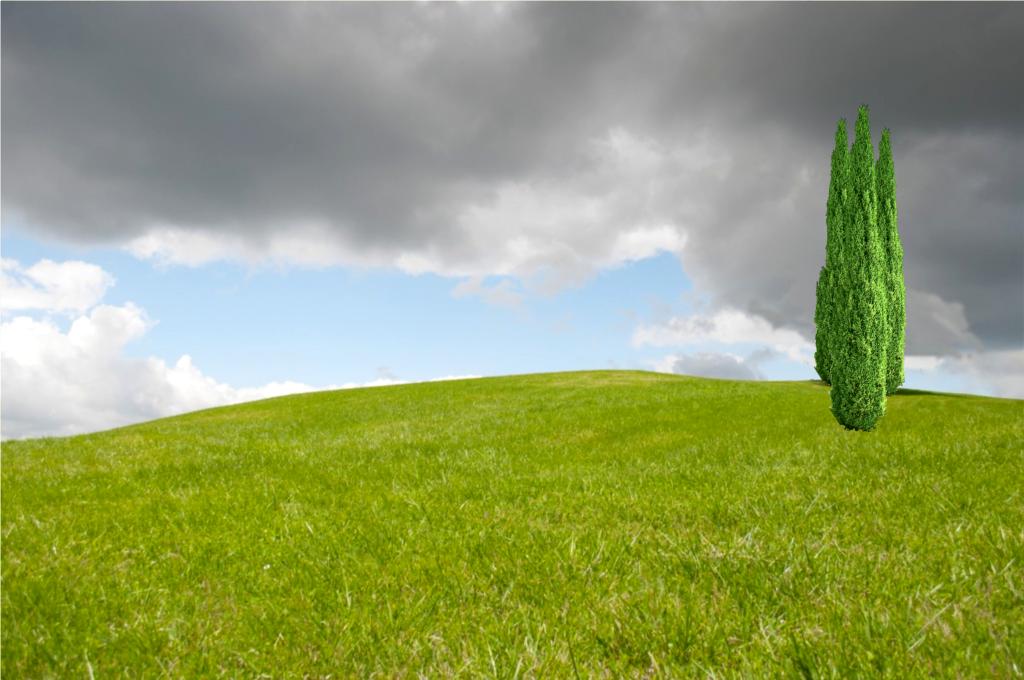

Supplement: Supplementary file 1 [file Data_Sheet_1.ZIP › dispersion low 3.png]

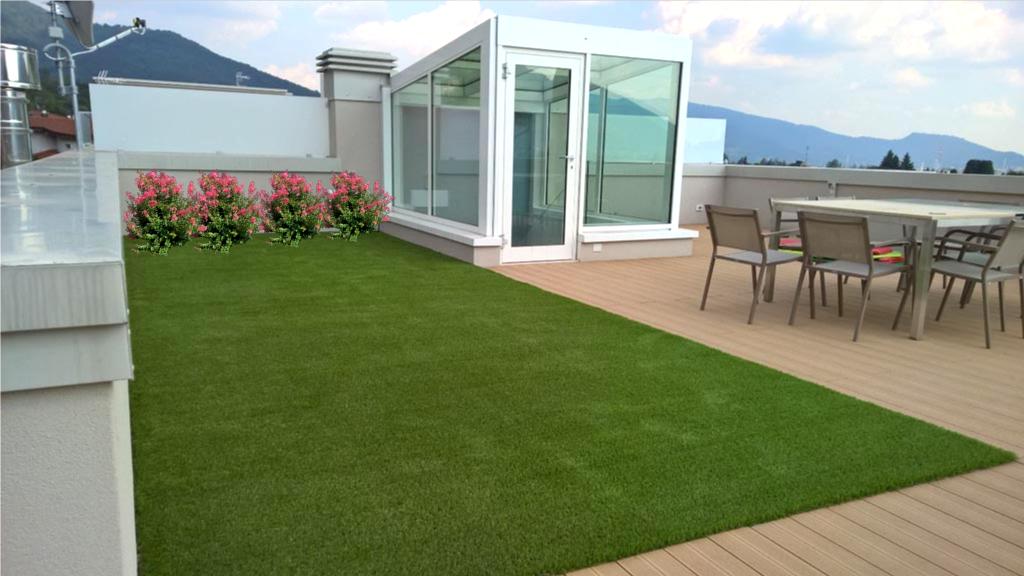

Supplement: Supplementary file 1 [file Data_Sheet_1.ZIP › dispersion low 30.png]

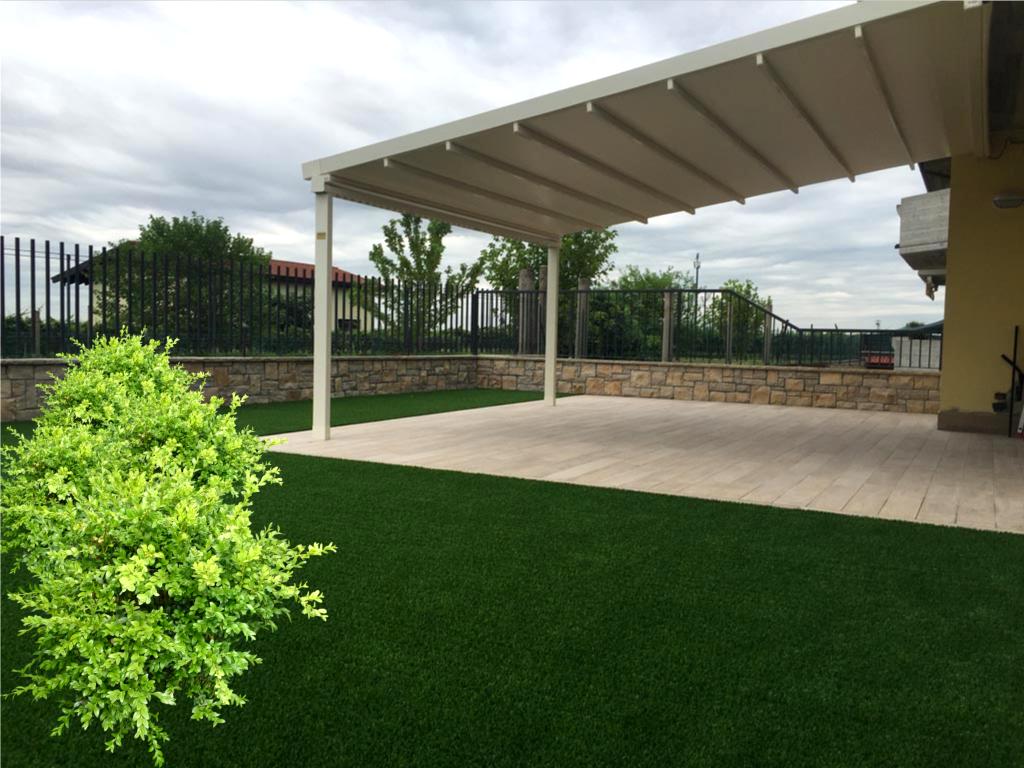

Supplement: Supplementary file 1 [file Data_Sheet_1.ZIP › dispersion low 31.png]

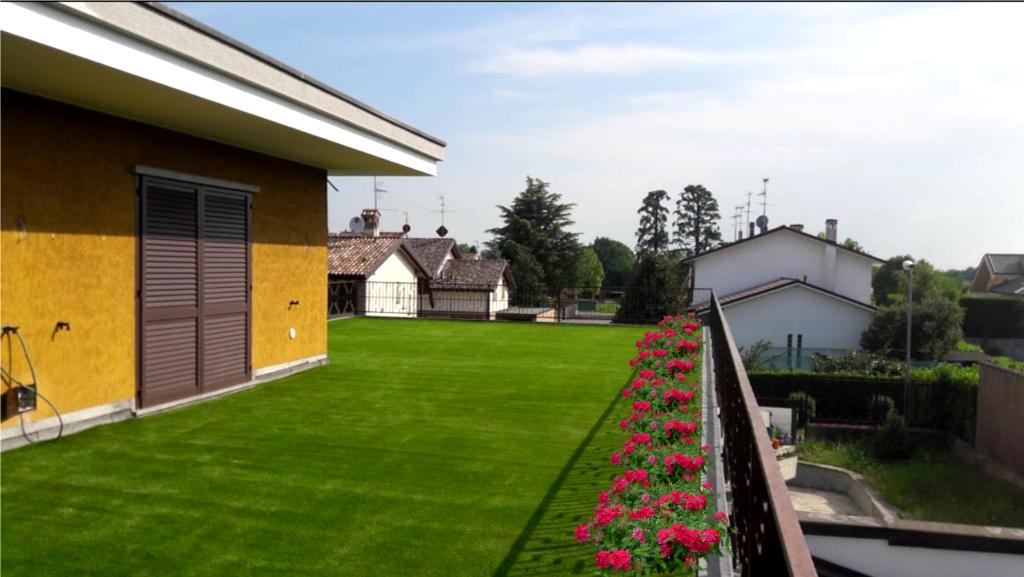

Supplement: Supplementary file 1 [file Data_Sheet_1.ZIP › dispersion low 32.png]

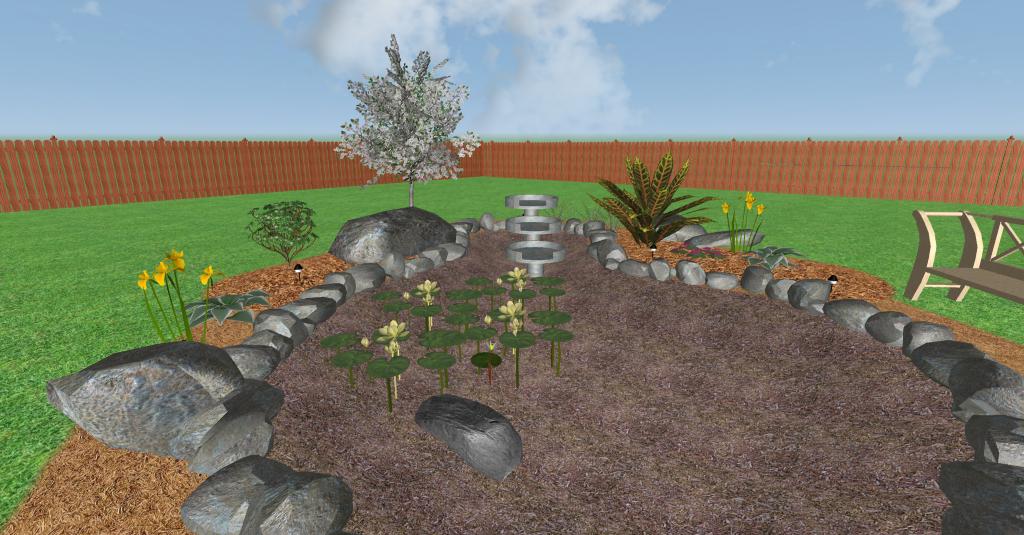

Supplement: Supplementary file 1 [file Data_Sheet_1.ZIP › dispersion low 33.png]

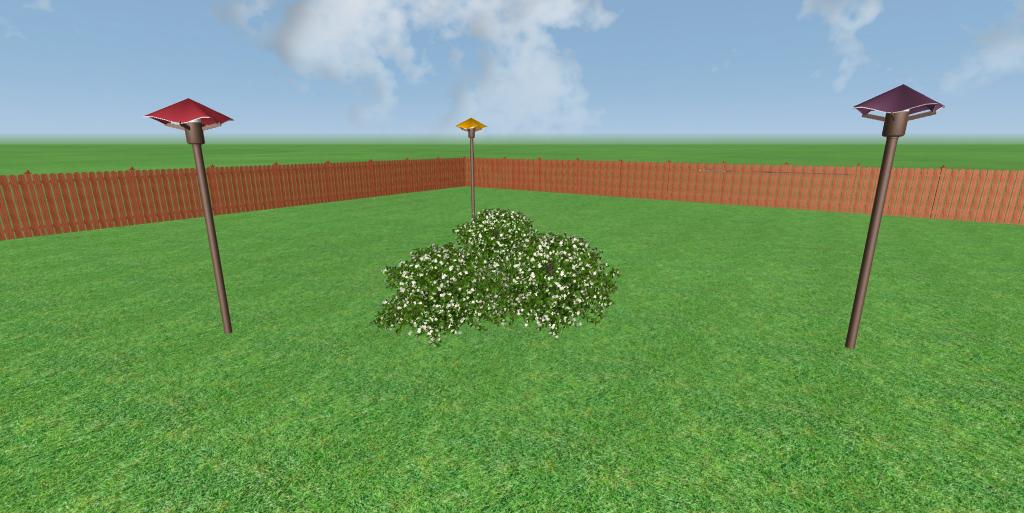

Supplement: Supplementary file 1 [file Data_Sheet_1.ZIP › dispersion low 34.png]

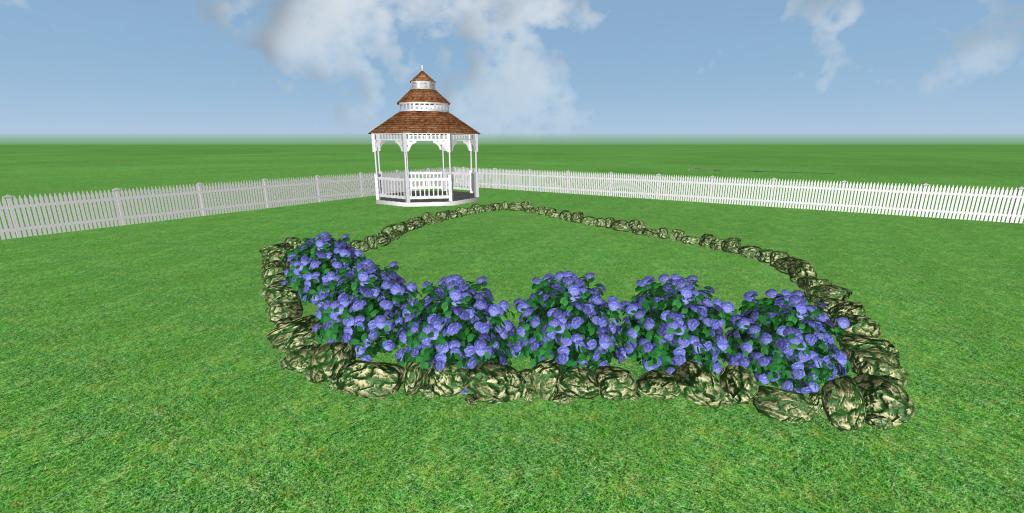

Supplement: Supplementary file 1 [file Data_Sheet_1.ZIP › dispersion low 35.png]

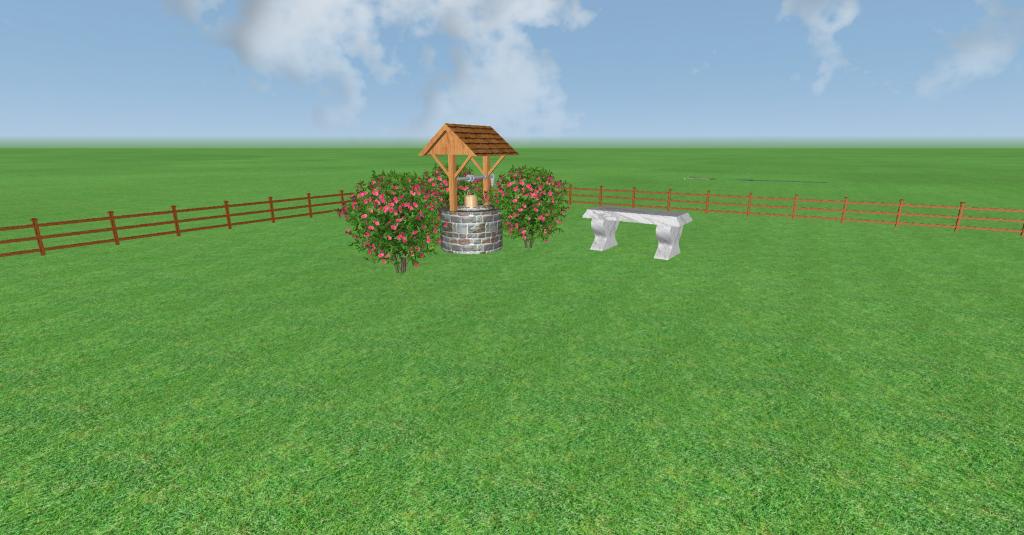

Supplement: Supplementary file 1 [file Data_Sheet_1.ZIP › dispersion low 36.png]

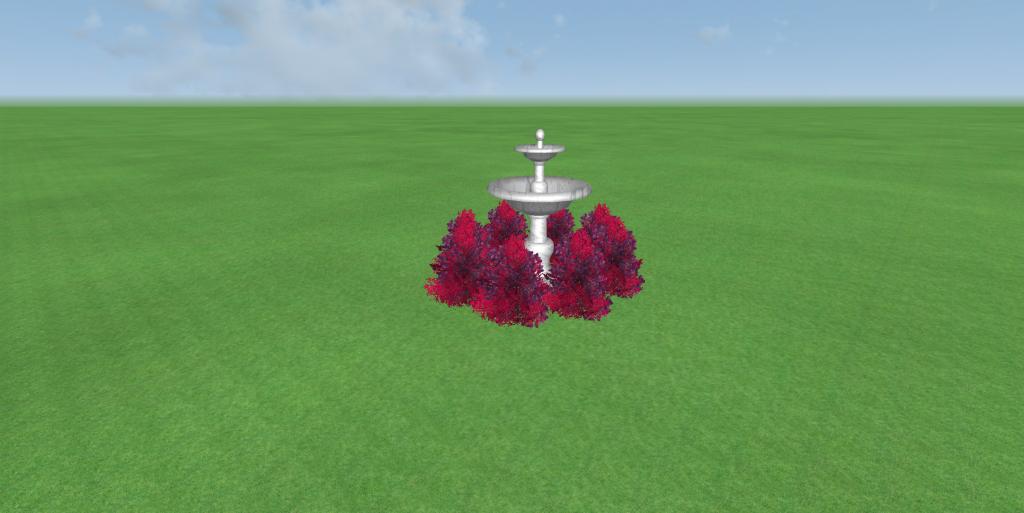

Supplement: Supplementary file 1 [file Data_Sheet_1.ZIP › dispersion low 37.png]

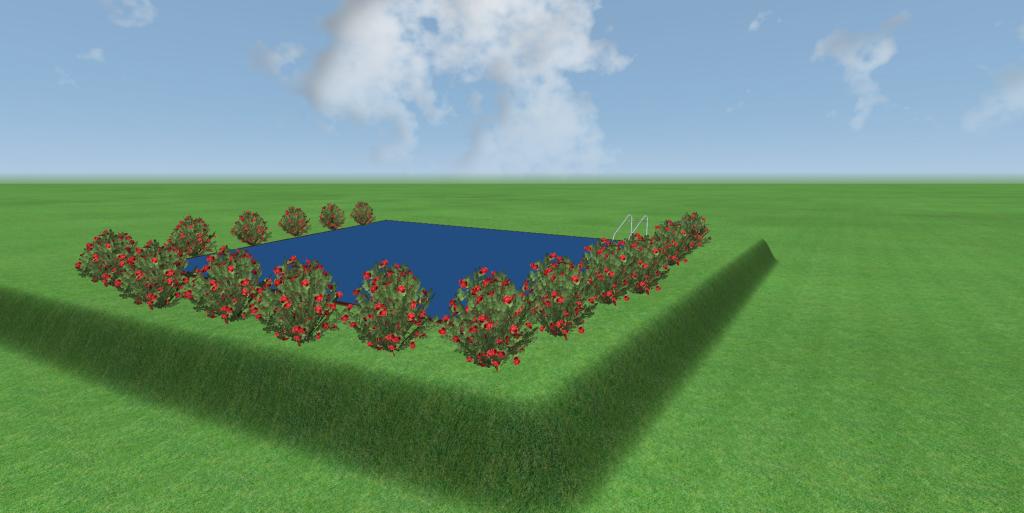

Supplement: Supplementary file 1 [file Data_Sheet_1.ZIP › dispersion low 38.png]

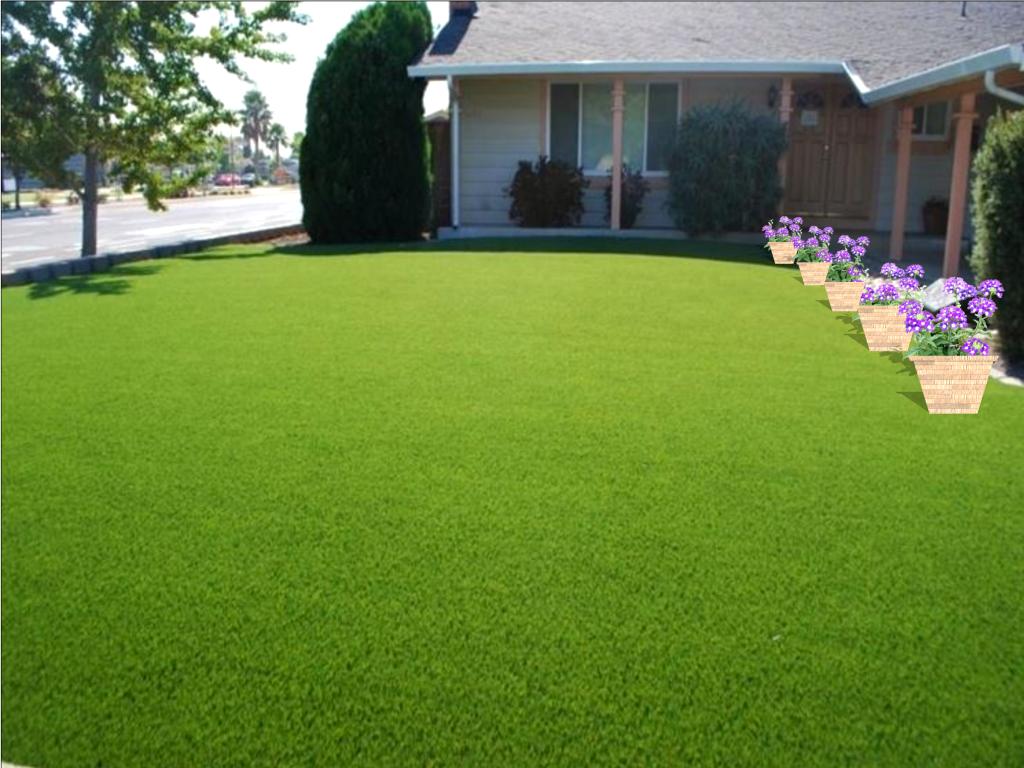

Supplement: Supplementary file 1 [file Data_Sheet_1.ZIP › dispersion low 39.png]

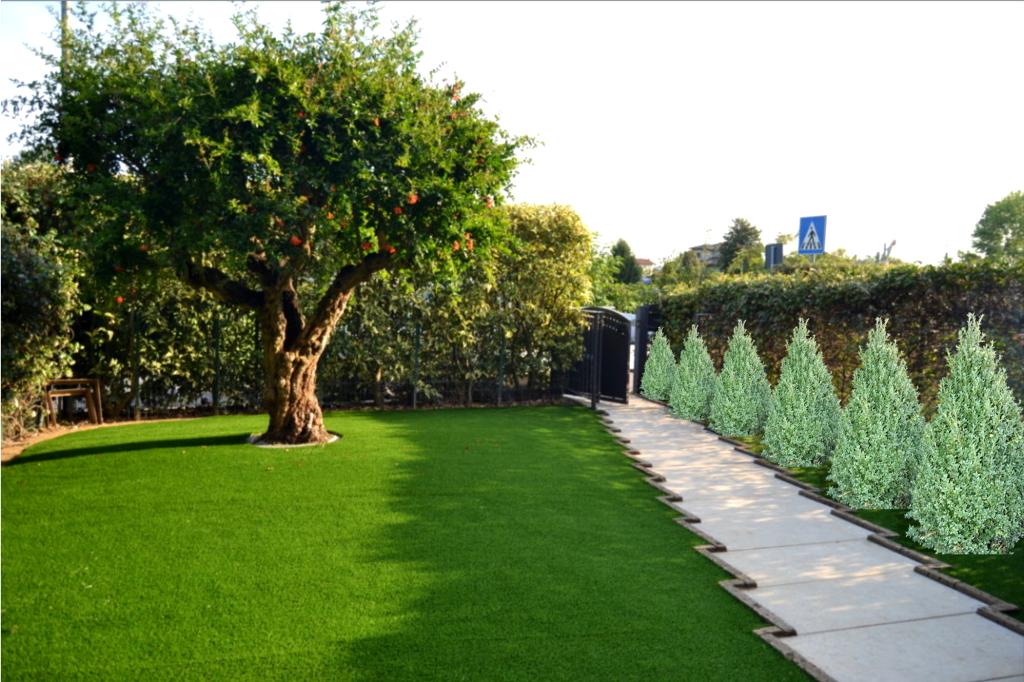

Supplement: Supplementary file 1 [file Data_Sheet_1.ZIP › dispersion low 40.png]

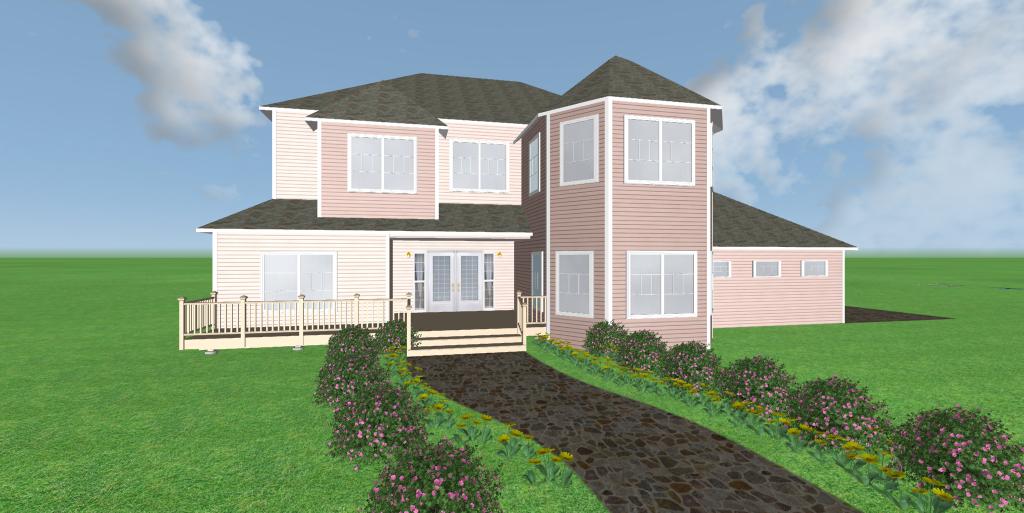

Supplement: Supplementary file 1 [file Data_Sheet_1.ZIP › interpsersion low 4.png]

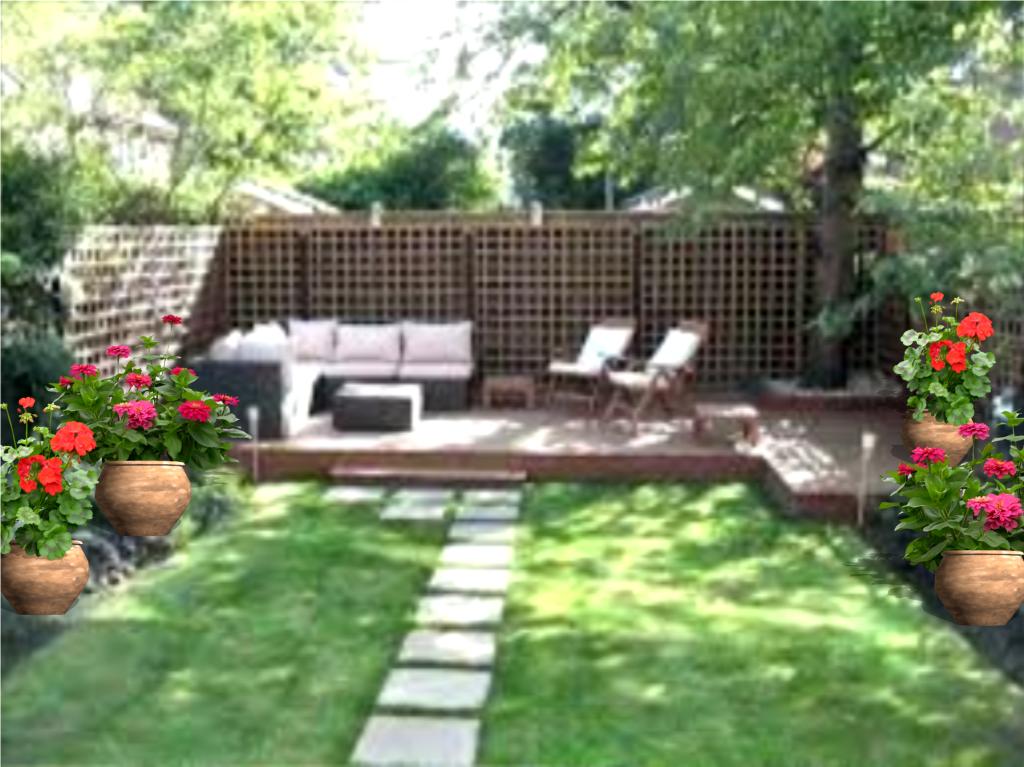

Supplement: Supplementary file 1 [file Data_Sheet_1.ZIP › interspersion high 10.png]

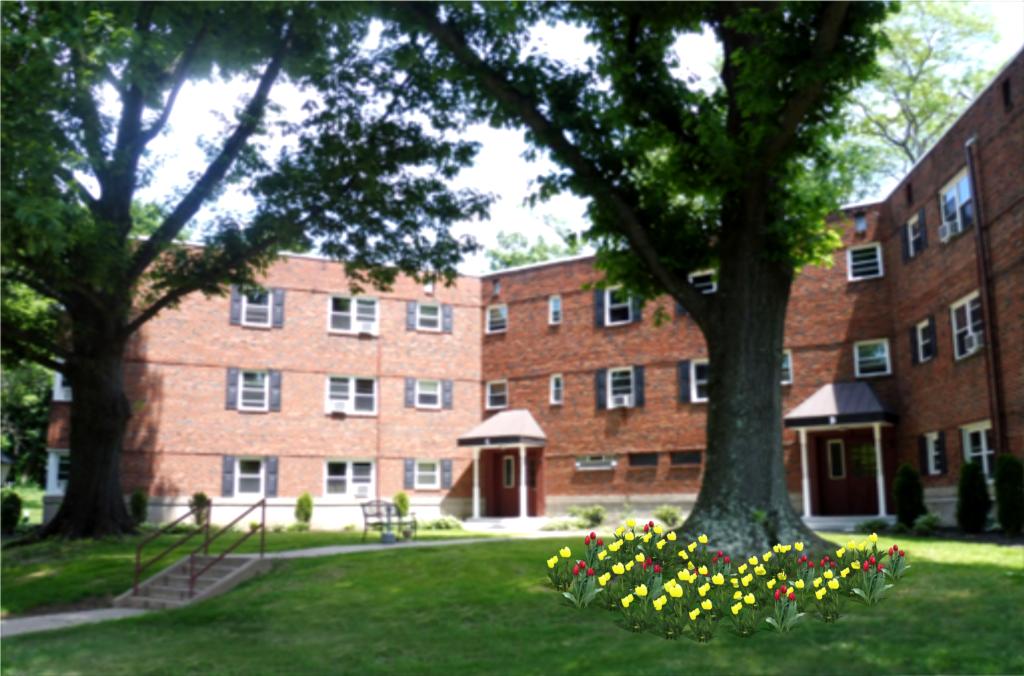

Supplement: Supplementary file 1 [file Data_Sheet_1.ZIP › interspersion high 12.png]

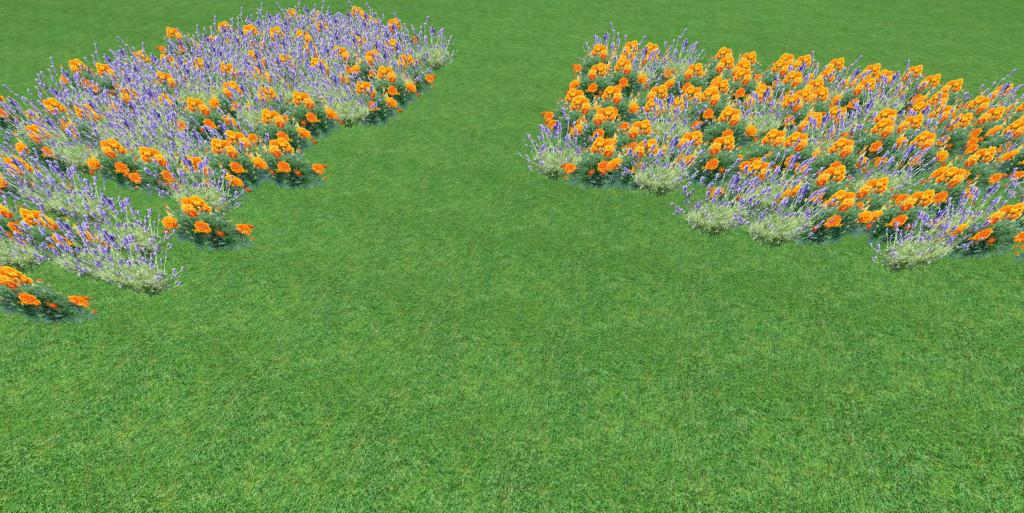

Supplement: Supplementary file 1 [file Data_Sheet_1.ZIP › interspersion high 13.png]

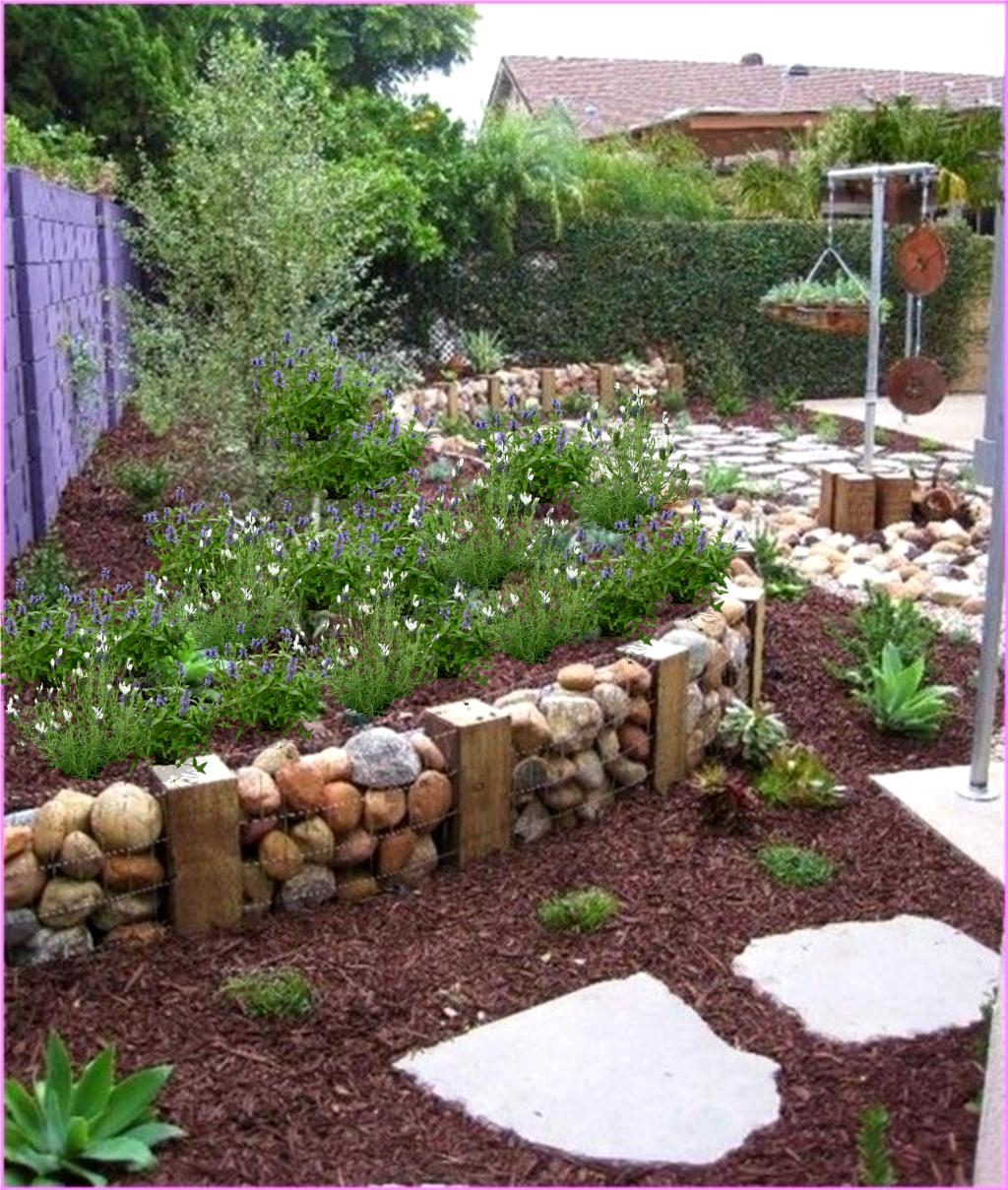

Supplement: Supplementary file 1 [file Data_Sheet_1.ZIP › interspersion high 14.png]

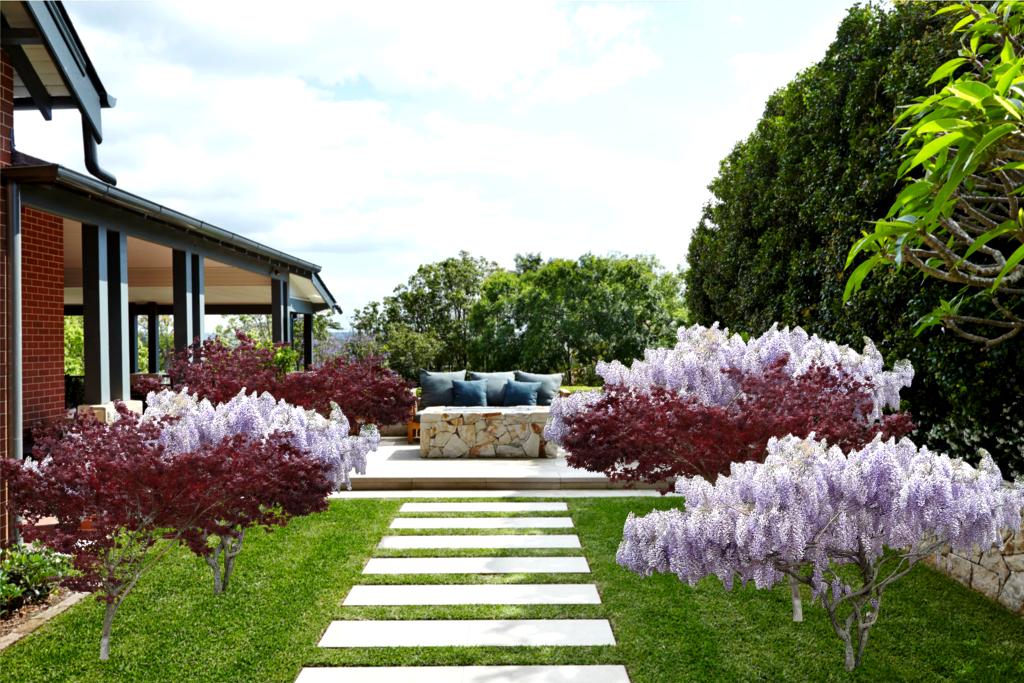

Supplement: Supplementary file 1 [file Data_Sheet_1.ZIP › interspersion high 15.png]

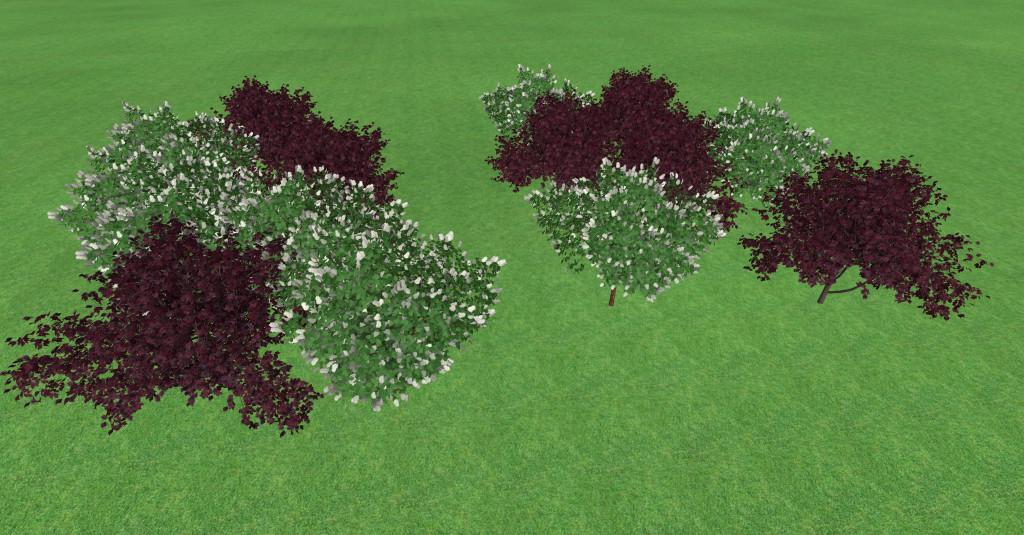

Supplement: Supplementary file 1 [file Data_Sheet_1.ZIP › interspersion high 16.png]

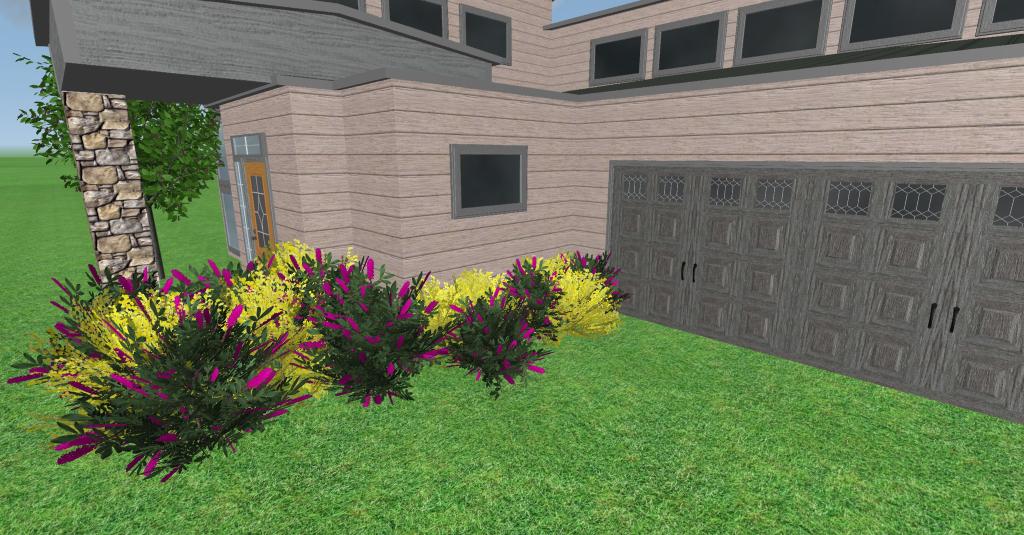

Supplement: Supplementary file 1 [file Data_Sheet_1.ZIP › interspersion high 17.png]

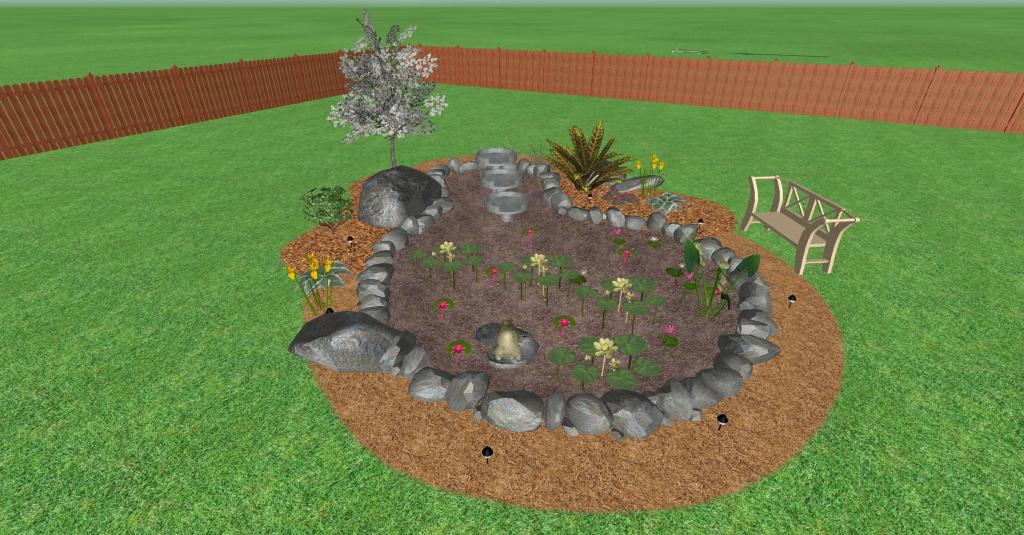

Supplement: Supplementary file 1 [file Data_Sheet_1.ZIP › interspersion high 18.png]

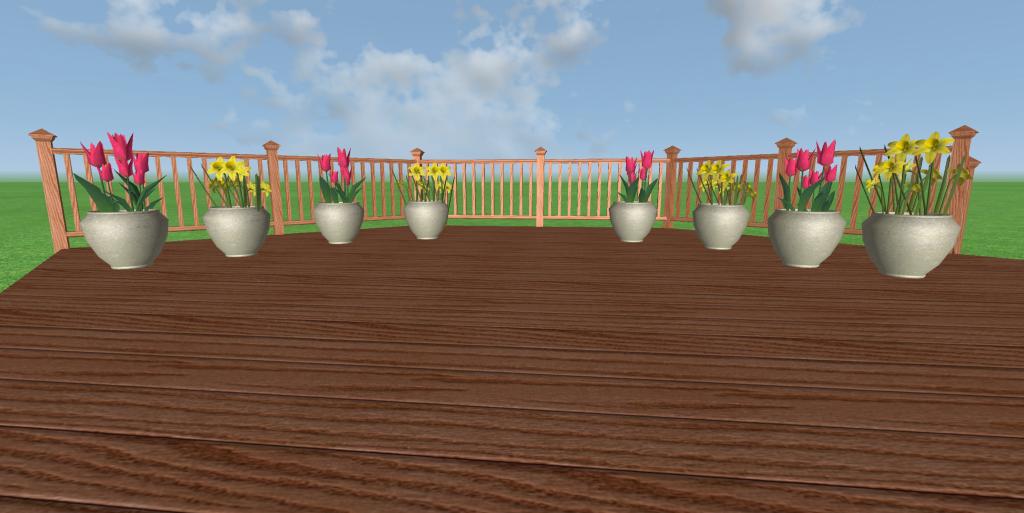

Supplement: Supplementary file 1 [file Data_Sheet_1.ZIP › interspersion high 19.png]

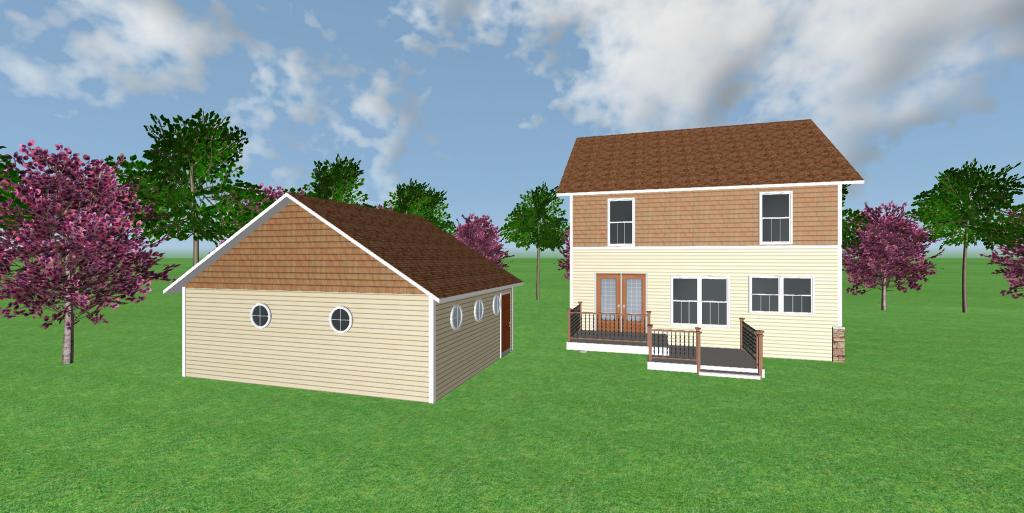

Supplement: Supplementary file 1 [file Data_Sheet_1.ZIP › interspersion high 2.png]

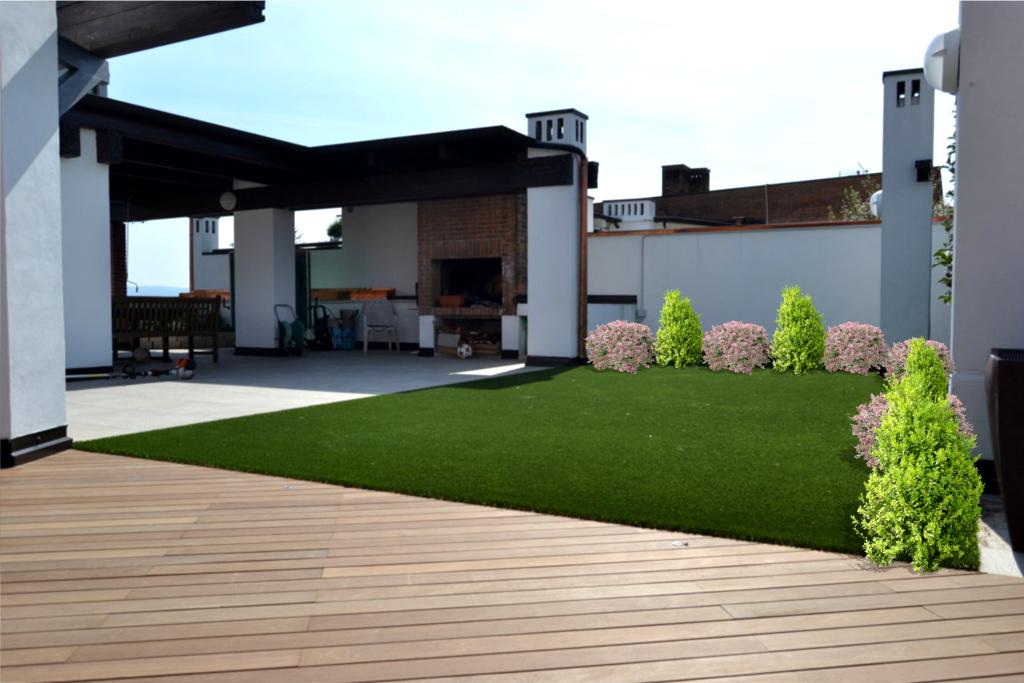

Supplement: Supplementary file 1 [file Data_Sheet_1.ZIP › interspersion high 20.png]

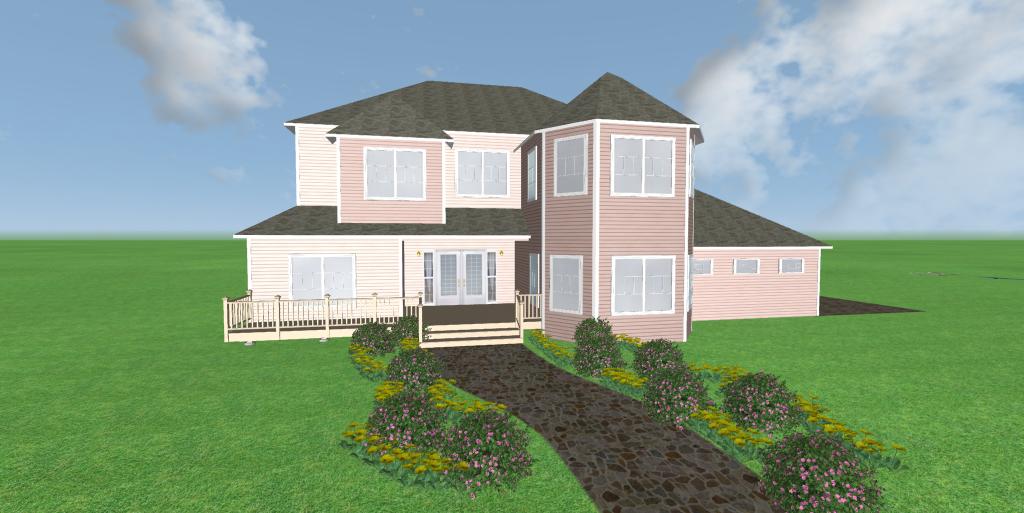

Supplement: Supplementary file 1 [file Data_Sheet_1.ZIP › interspersion high 4.png]

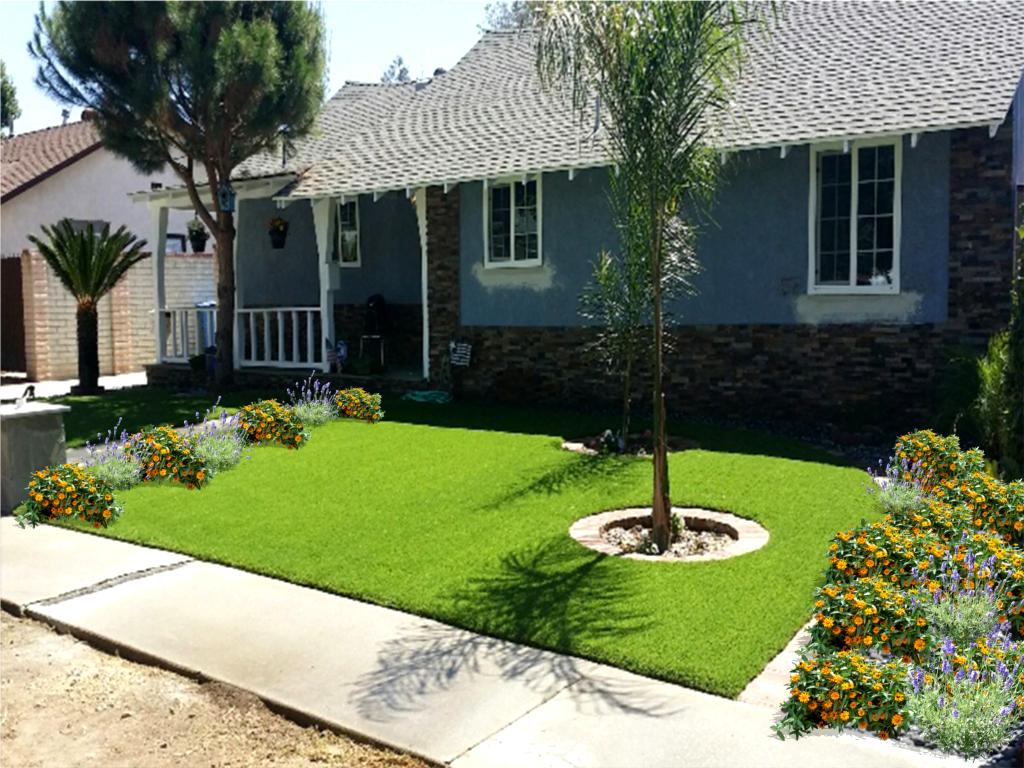

Supplement: Supplementary file 1 [file Data_Sheet_1.ZIP › interspersion high 5.png]

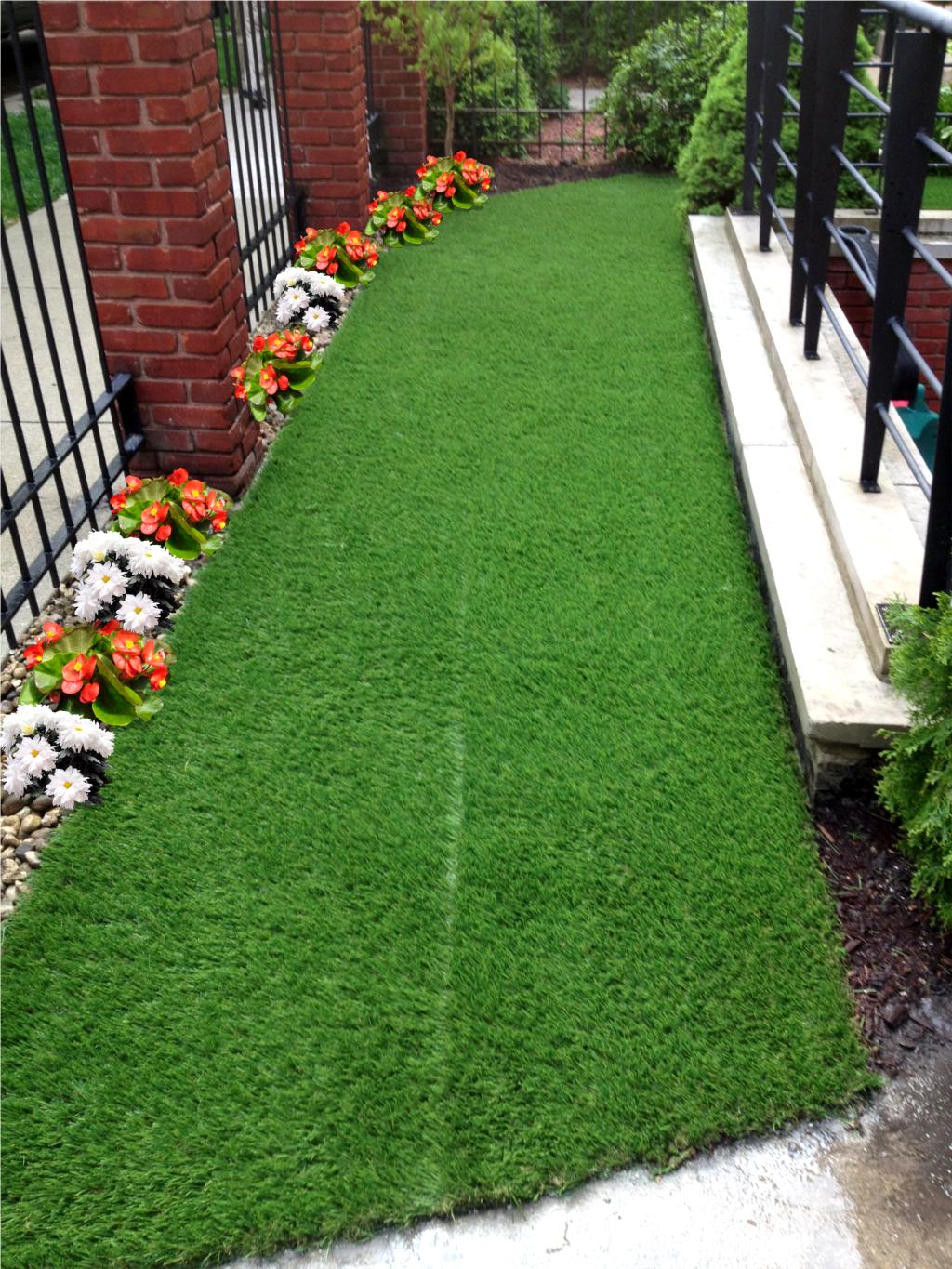

Supplement: Supplementary file 1 [file Data_Sheet_1.ZIP › interspersion high 6.png]

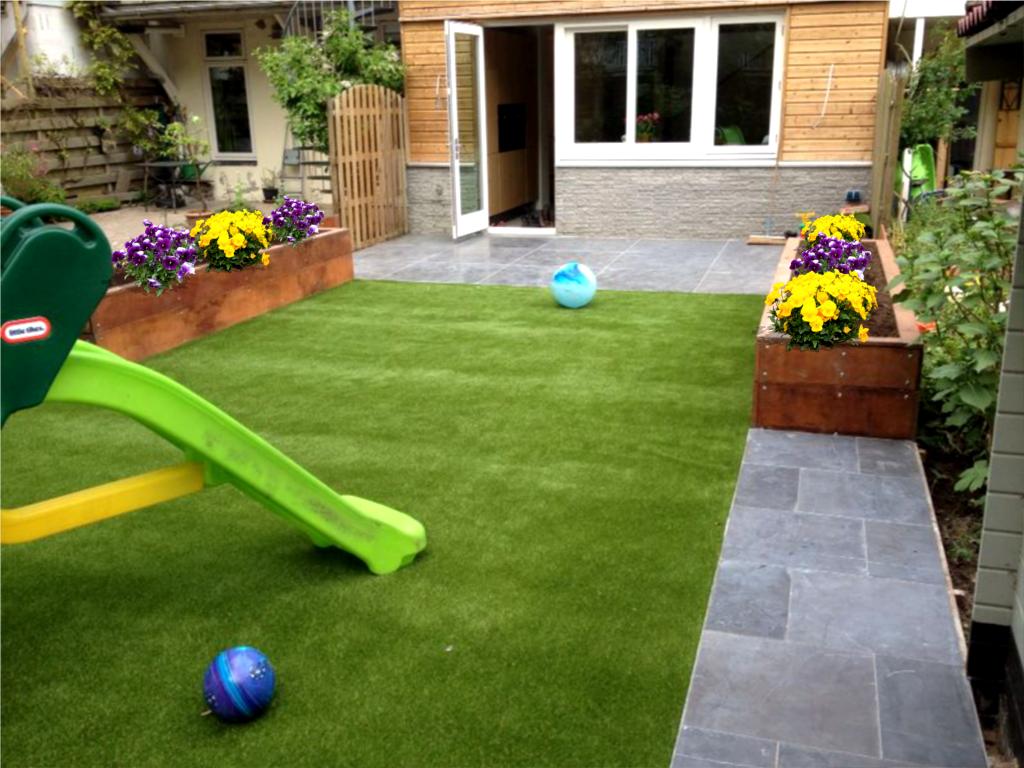

Supplement: Supplementary file 1 [file Data_Sheet_1.ZIP › interspersion high 7.png]

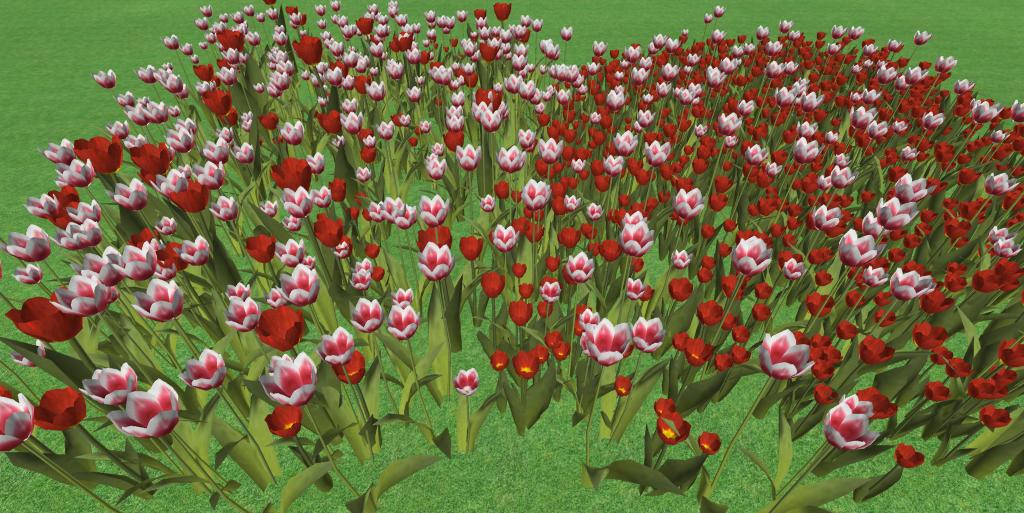

Supplement: Supplementary file 1 [file Data_Sheet_1.ZIP › interspersion high 8.png]

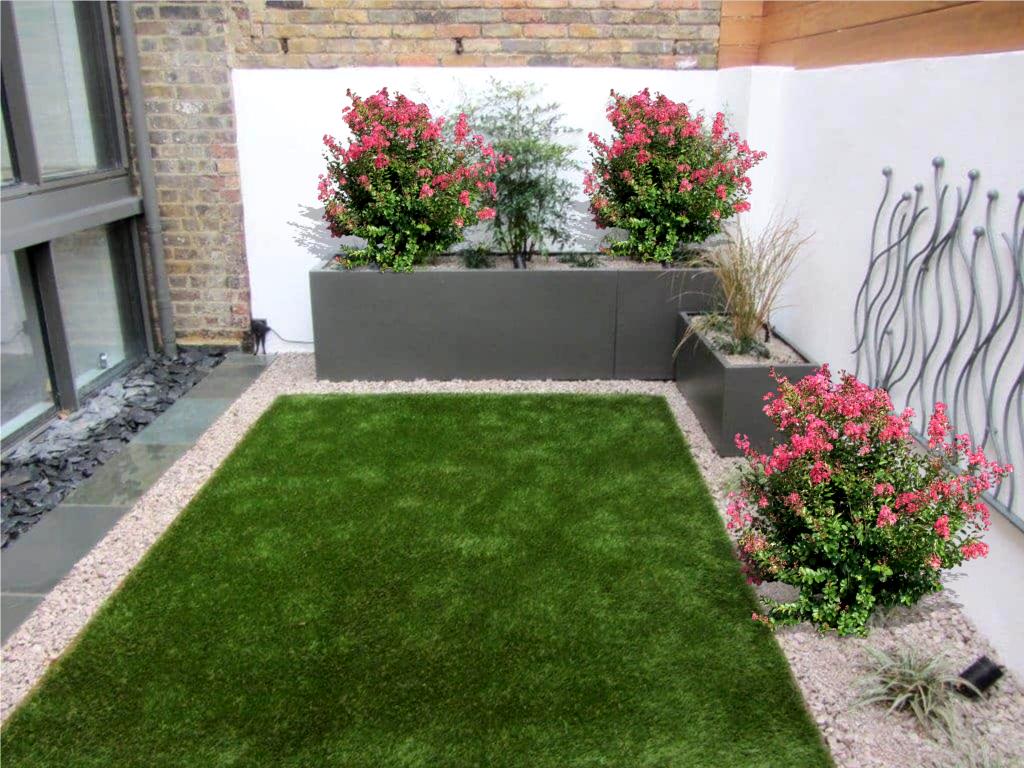

Supplement: Supplementary file 1 [file Data_Sheet_1.ZIP › interspersion high 9.png]

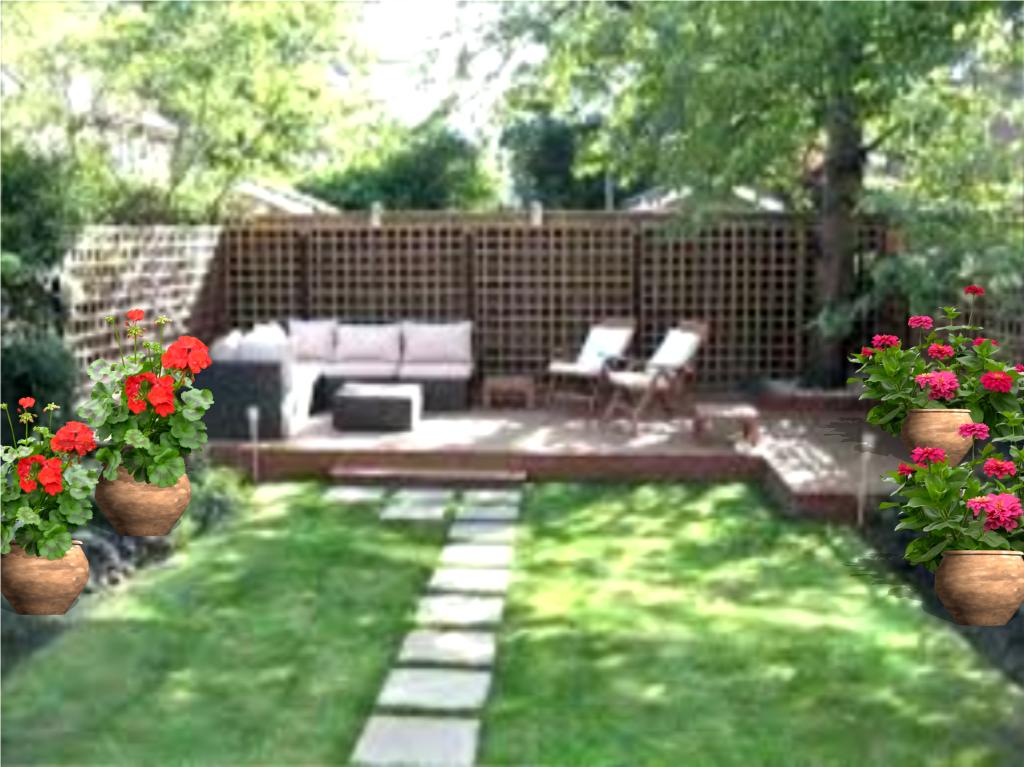

Supplement: Supplementary file 1 [file Data_Sheet_1.ZIP › interspersion low 10.png]

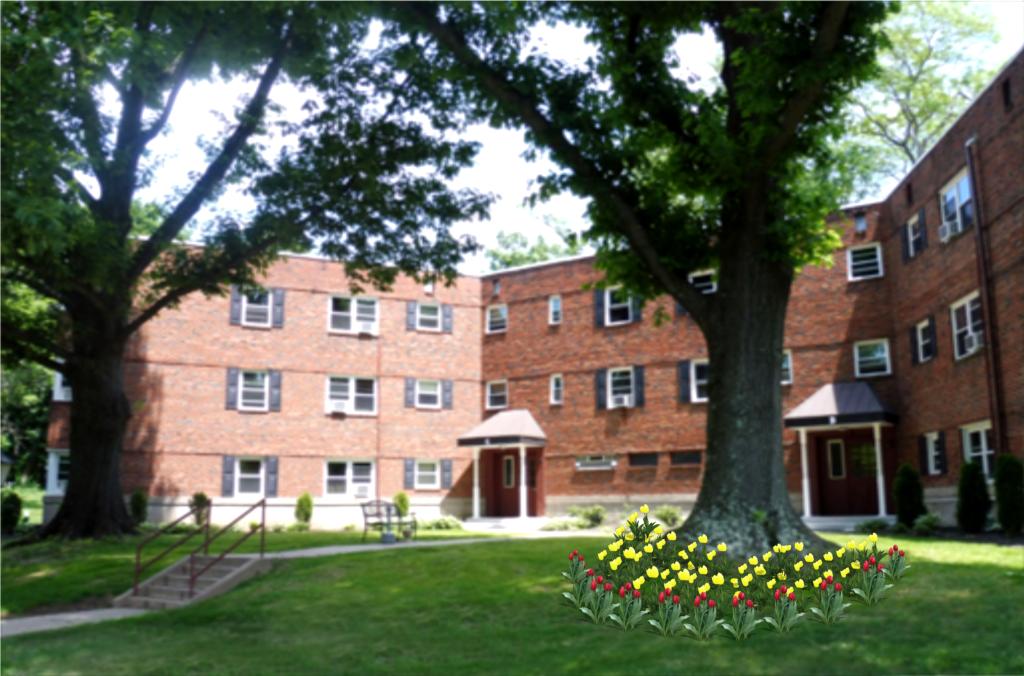

Supplement: Supplementary file 1 [file Data_Sheet_1.ZIP › interspersion low 12.png]

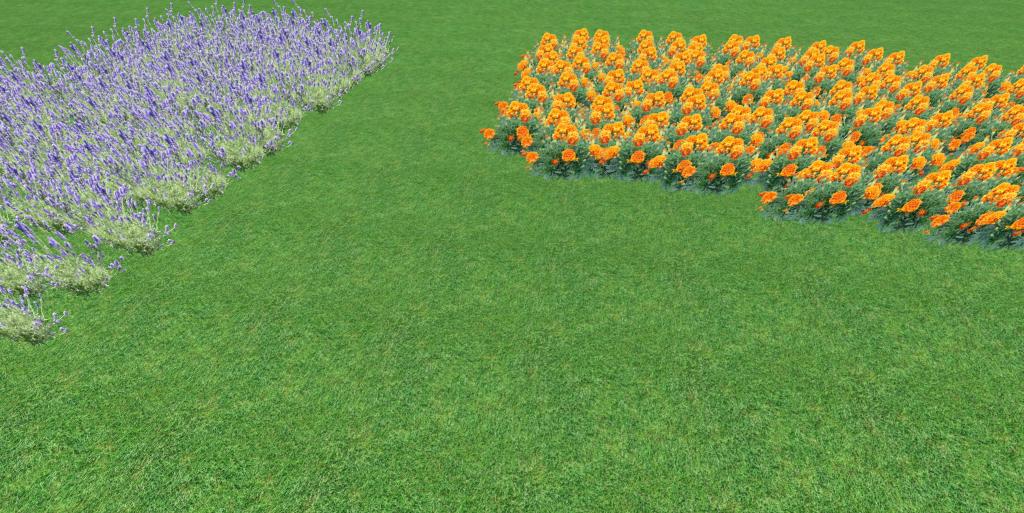

Supplement: Supplementary file 1 [file Data_Sheet_1.ZIP › interspersion low 13.png]

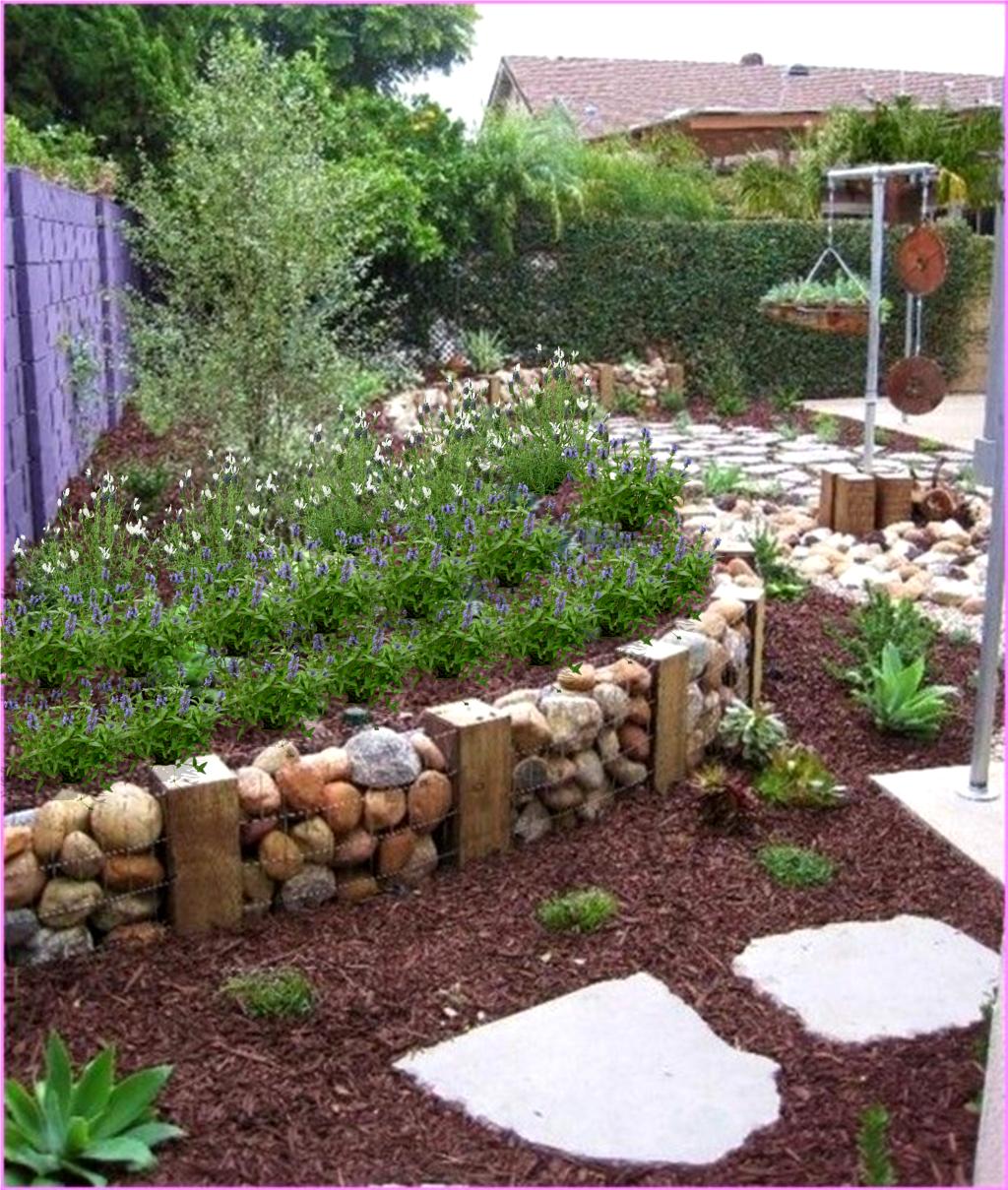

Supplement: Supplementary file 1 [file Data_Sheet_1.ZIP › interspersion low 14.png]

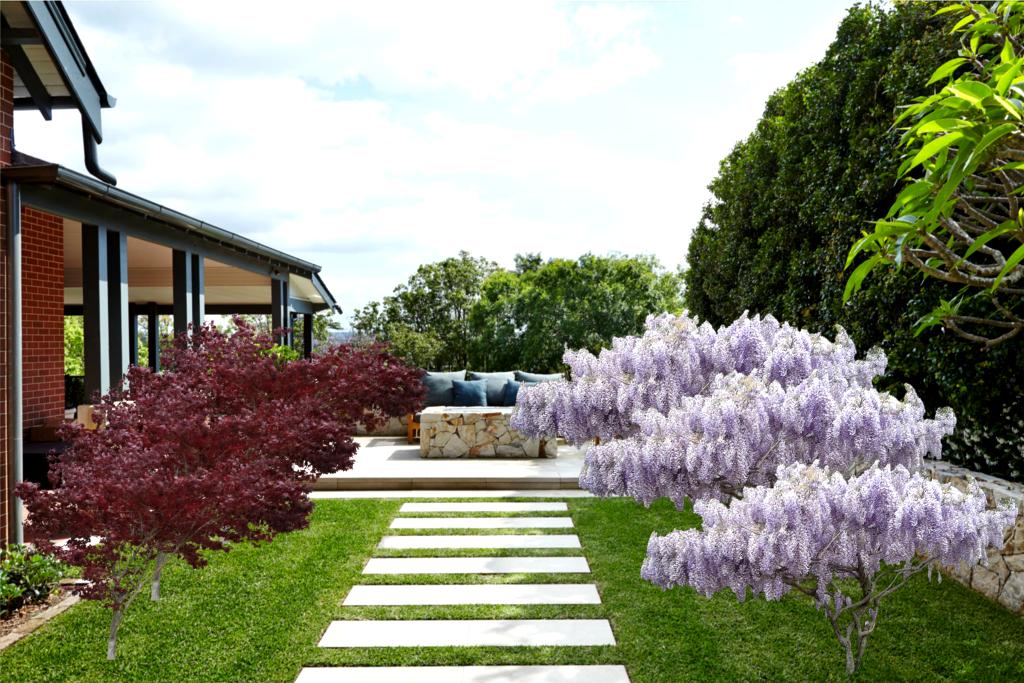

Supplement: Supplementary file 1 [file Data_Sheet_1.ZIP › interspersion low 15.png]

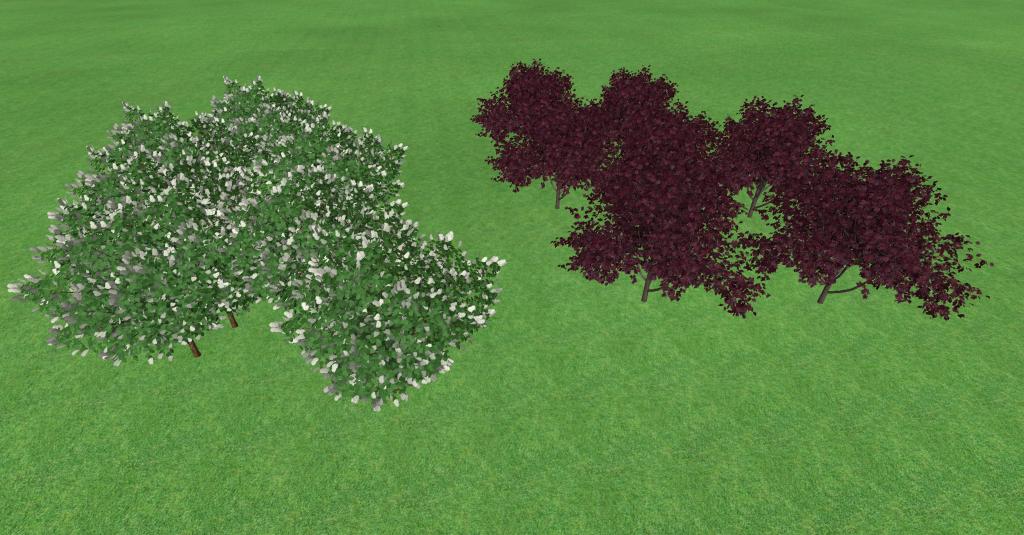

Supplement: Supplementary file 1 [file Data_Sheet_1.ZIP › interspersion low 16.png]

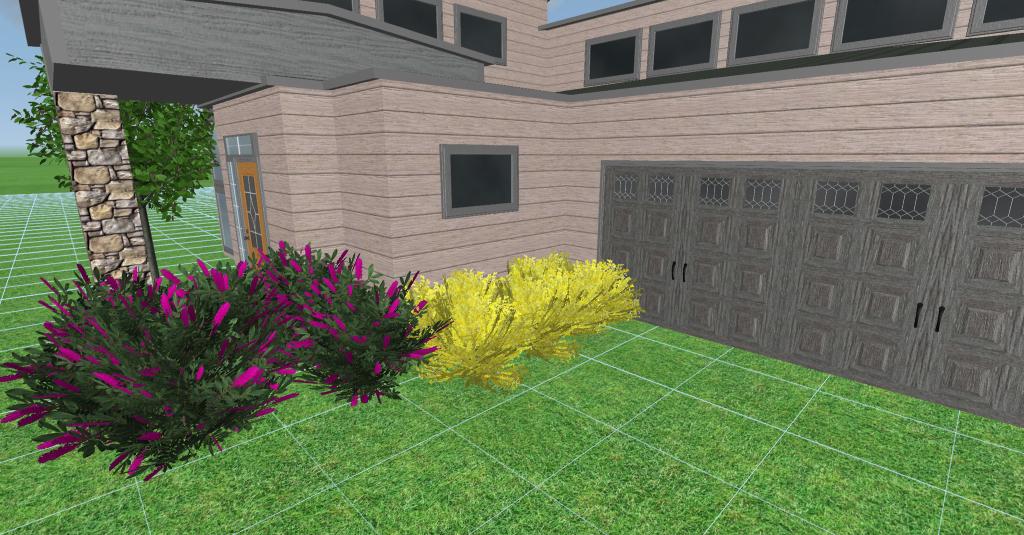

Supplement: Supplementary file 1 [file Data_Sheet_1.ZIP › interspersion low 17.png]

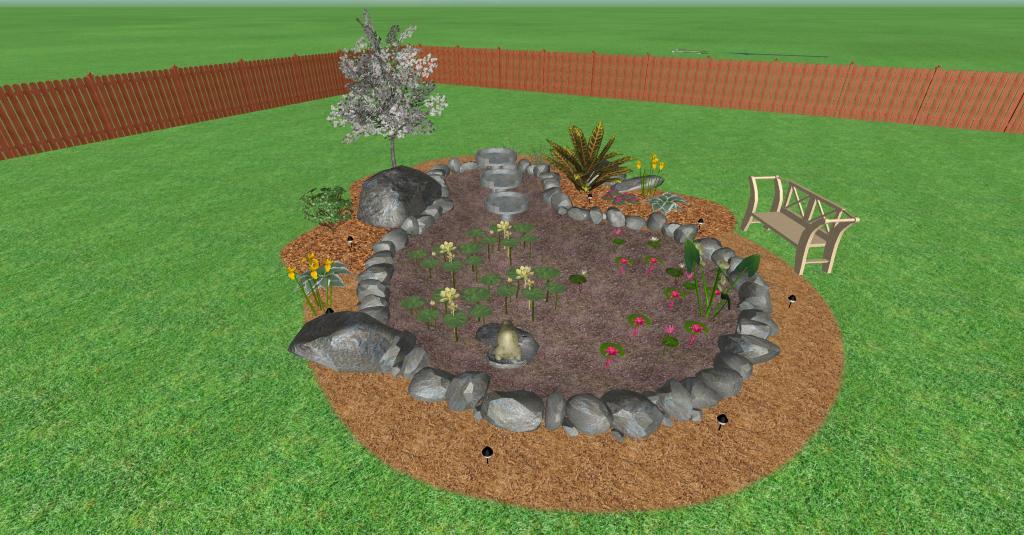

Supplement: Supplementary file 1 [file Data_Sheet_1.ZIP › interspersion low 18.png]

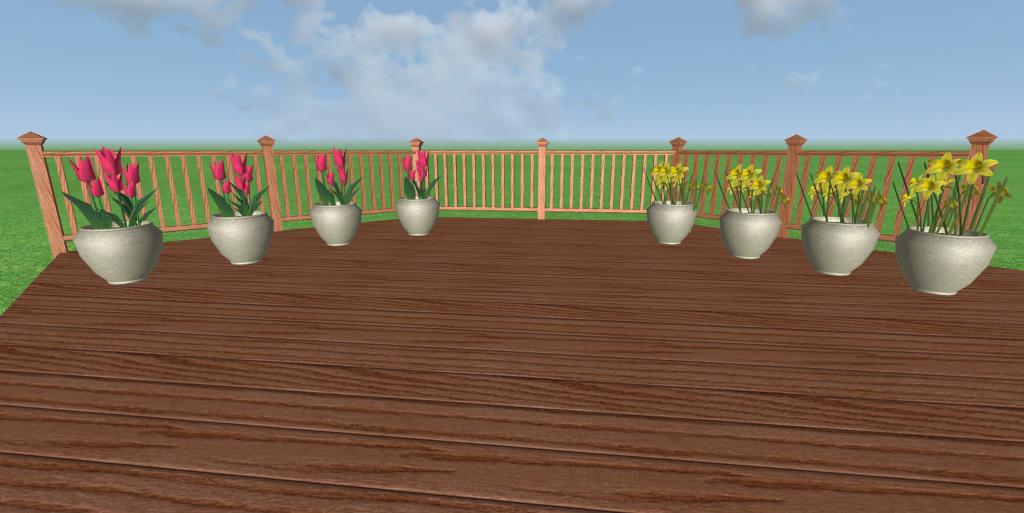

Supplement: Supplementary file 1 [file Data_Sheet_1.ZIP › interspersion low 19.png]

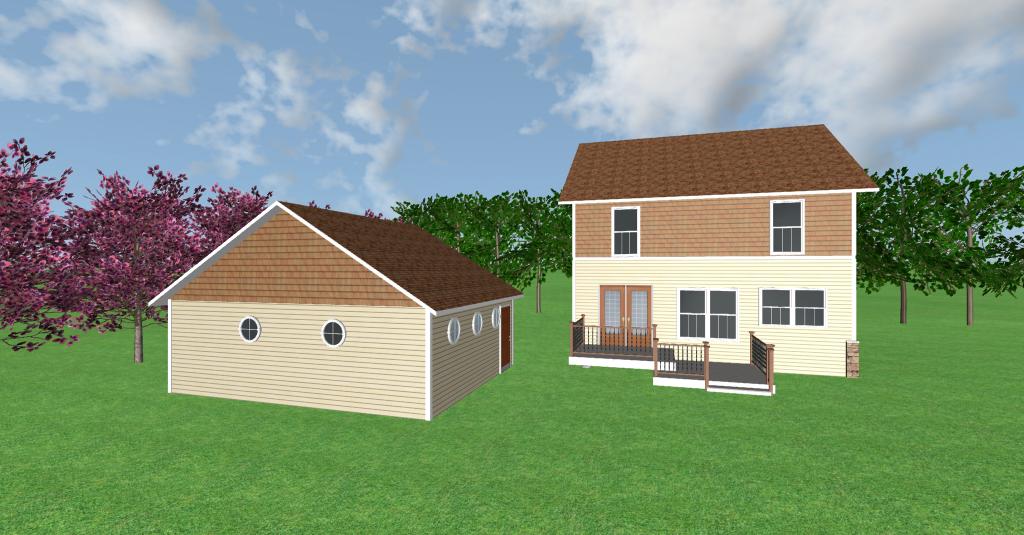

Supplement: Supplementary file 1 [file Data_Sheet_1.ZIP › interspersion low 2.png]

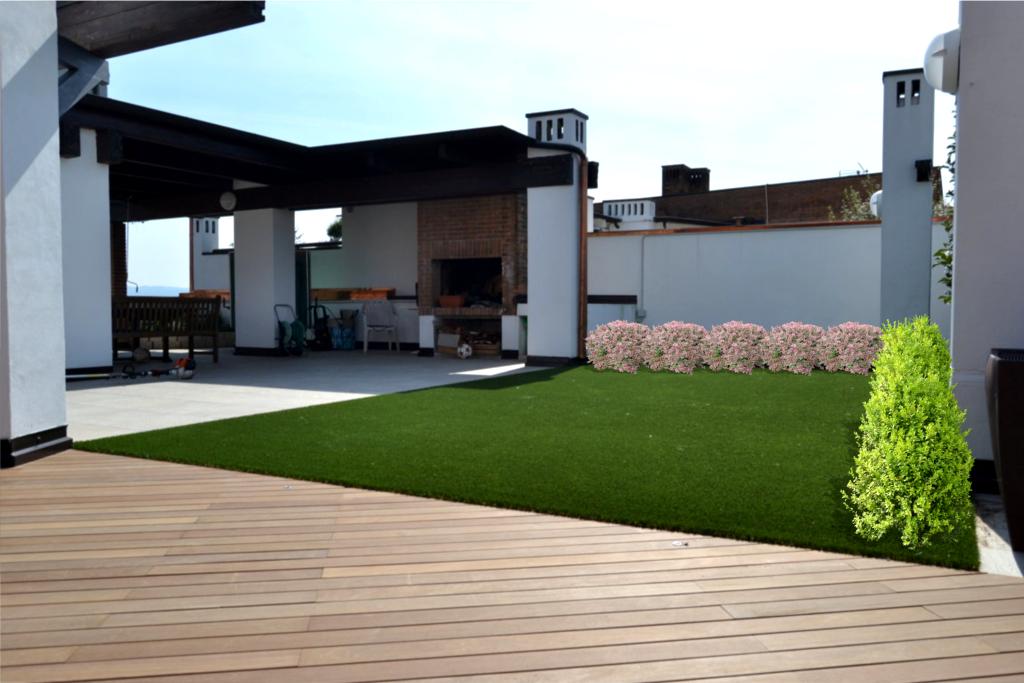

Supplement: Supplementary file 1 [file Data_Sheet_1.ZIP › interspersion low 20.png]

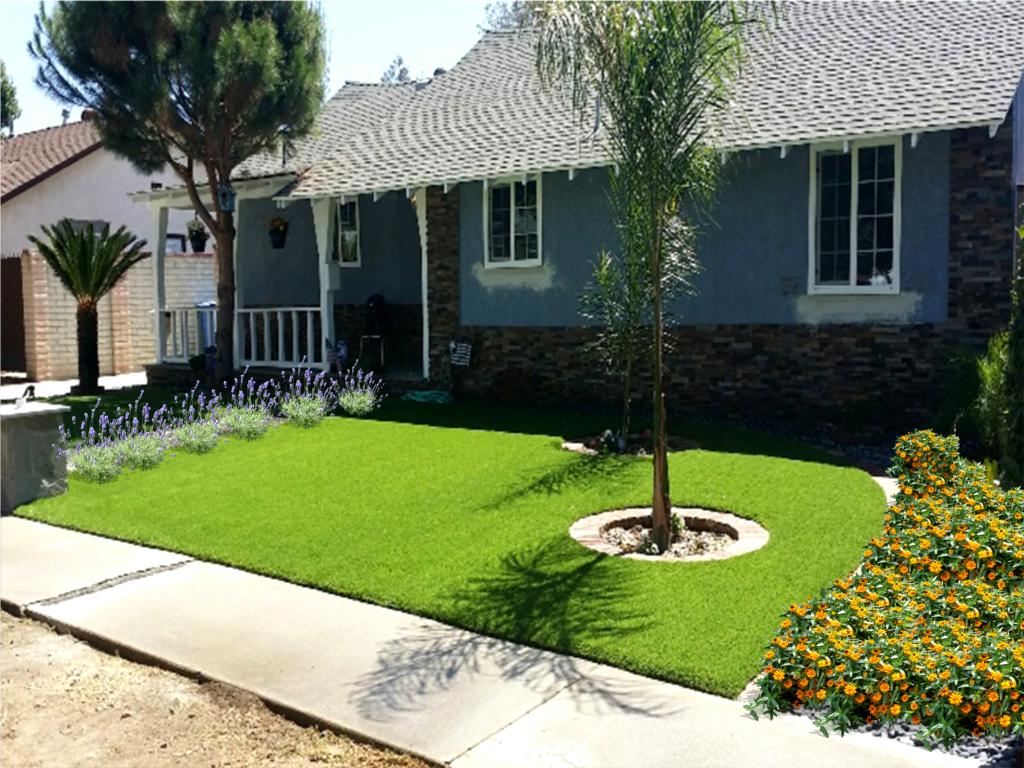

Supplement: Supplementary file 1 [file Data_Sheet_1.ZIP › interspersion low 5.png]

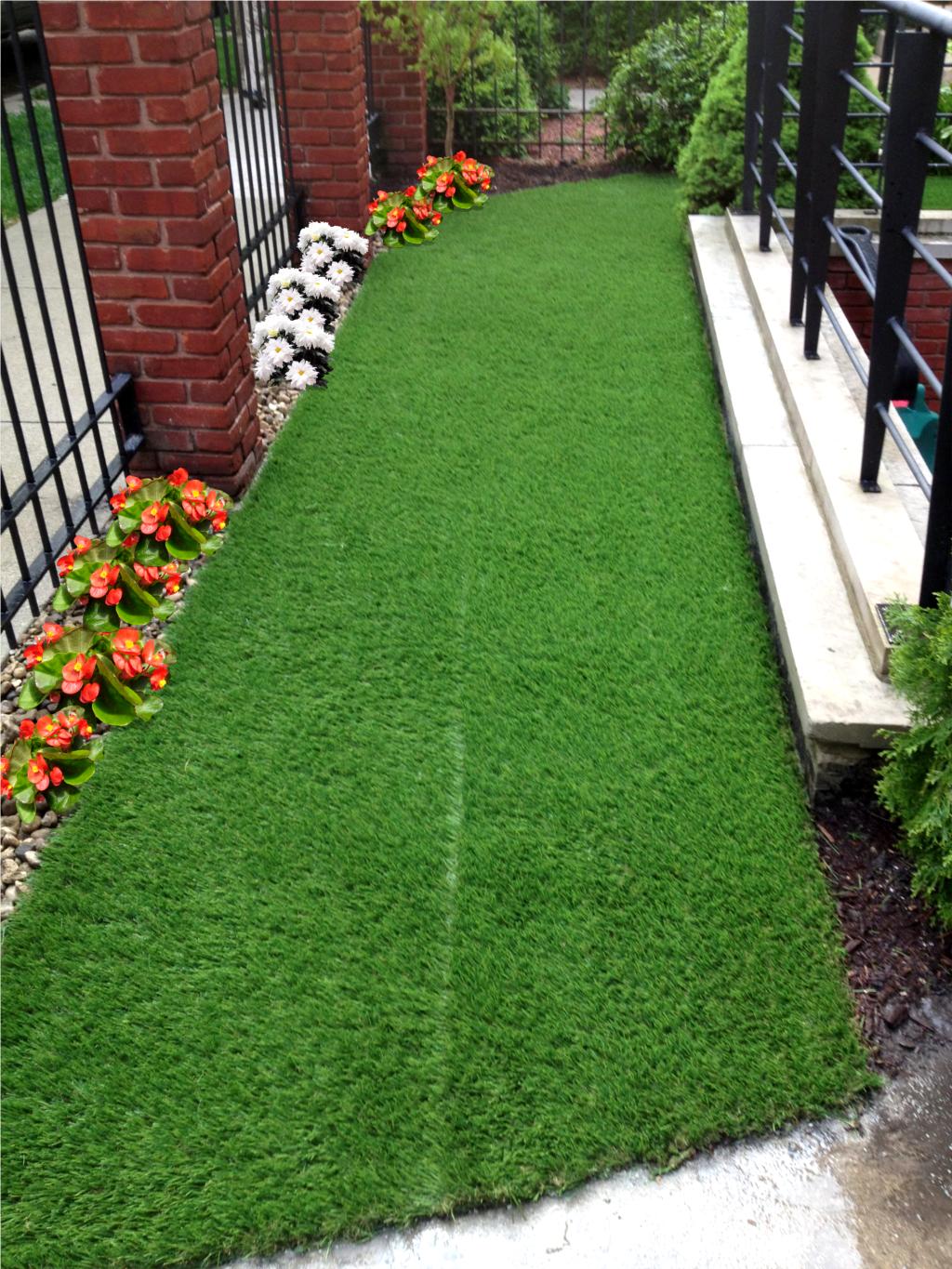

Supplement: Supplementary file 1 [file Data_Sheet_1.ZIP › interspersion low 6.png]

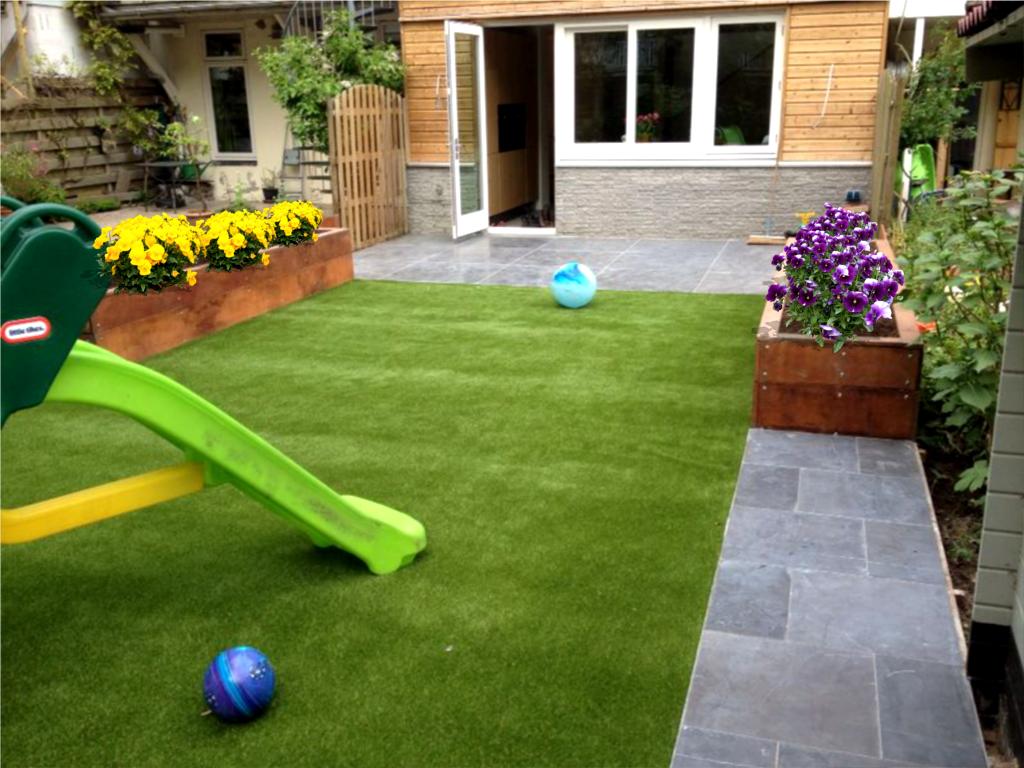

Supplement: Supplementary file 1 [file Data_Sheet_1.ZIP › interspersion low 7.png]

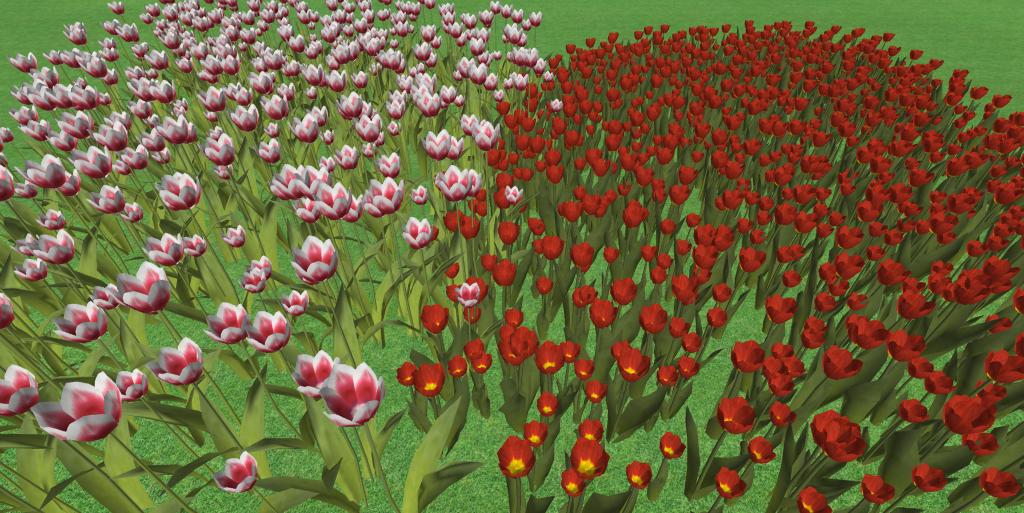

Supplement: Supplementary file 1 [file Data_Sheet_1.ZIP › interspersion low 8.png]

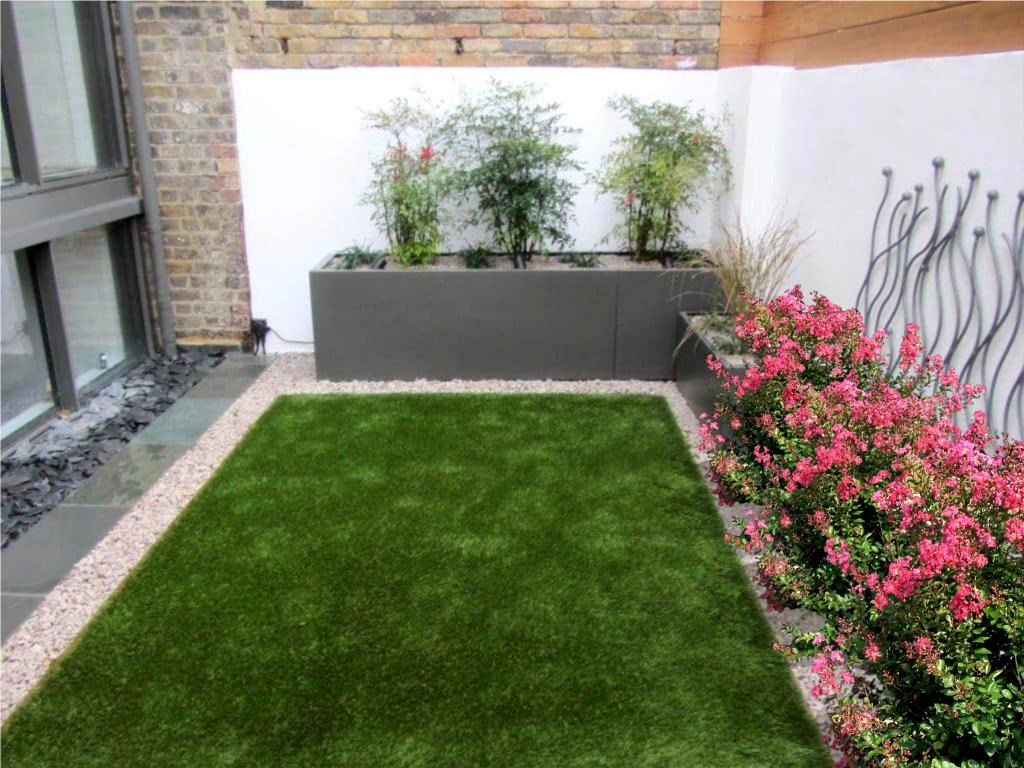

Supplement: Supplementary file 1 [file Data_Sheet_1.ZIP › interspersion low 9.png]
